# Oxadiazolone‐Triazole Derivatives as a New Scaffold for Modulation of Angiotensin II‐Induced Vascular Contraction

**DOI:** 10.1002/cmdc.70460

**Published:** 2026-08-31

**Authors:** Larissa F. L. Ferreira, Daiana G. de A. Vasconcelos, Ruth C. A. Santos, Jivaldo G. Ferreira, Alice V. Araújo, Janaína V. dos Anjos

**Affiliations:** ^1^ Department of Fundamental Chemistry Federal University of Pernambuco (UFPE) Recife Brazil; ^2^ Vitória Academic Centre (CAV) Federal University of Pernambuco (UFPE) Vitória de Santo Antão Pernambuco Brazil

**Keywords:** angiotensin receptor blockers (ARBs), click chemistry, heterocycles, hypertension

## Abstract

In this study, a series of novel angiotensin II type 1 receptor (AT_1_R) antagonist candidates containing the 1,2,4‐oxadiazol‐5‐one nucleus and a 1,2,3‐triazole moiety was designed, synthesized, and evaluated. Compounds **7a**–**j** were obtained via a semiconvergent synthetic route employing copper‐catalyzed azide–alkyne cycloaddition (click chemistry), yielding the target molecules in moderate to good yields. Biological activity was assessed through vascular reactivity assays using rat aortic rings in the presence of angiotensin II. Most compounds reduced angiotensin II‐induced contraction, consistent with a putative AT_1_ receptor antagonist profile. Among them, compound **7f**, bearing a methylenedioxyphenyl substituent, exhibited the most favorable biological profile within the series. The results indicate that electron‐donating groups favor activity, while strongly electron‐withdrawing substituents, such as nitro, are detrimental. Molecular docking studies predicted consistent interactions with key residues, particularly Arg167 and Tyr87, providing a plausible structural rationale for the observed biological activity. In silico ADME predictions indicated acceptable drug‐like properties, including compliance with drug‐likeness filters and a predicted bioavailability score, and no blood–brain barrier permeation.

## Graphical abstract

A novel series of candidate AT_1_ receptor antagonists based on oxadiazolone‐triazole scaffolds was designed and synthesized. Most compounds reduced angiotensin II‐induced vascular contraction in an initial biological screening, with compound **7f** emerging as the most active derivative. Molecular docking and in silico ADME studies provided a plausible structural rationale and identified this scaffold as a promising starting point for further optimization.

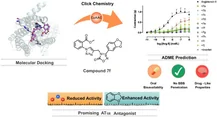

## 1 Introduction

Systemic arterial hypertension (SAH) is a multifactorial clinical condition characterized by elevated, sustained blood pressure. Population data indicate a high prevalence, affecting around 33% of the general adult population, with males being the most affected [1]. Treatment involves lifestyle modifications aimed at reducing cardiovascular risk factors, including a low‐salt, low‐fat diet, regular physical activity, and, often, pharmacological therapy [2, 3].

First‐line medications for the treatment of SAH include thiazide diuretics, angiotensin‐converting enzyme inhibitors (ACEIs), angiotensin receptor blockers (ARBs), calcium channel blockers (CCBs), and, in some cases, adrenergic beta‐blockers [4]. Angiotensin‐converting enzyme (ACE) inhibitors are effective drugs [5]; however, many patients require a second medication to achieve blood pressure targets [6], and they are not always well tolerated [7]. In the search for alternative ways to inhibit the renin–angiotensin system in patients with cardiovascular disease, angiotensin II receptor antagonists (ARBs) emerged as well‐tolerated and effective antihypertensive agents that provide cardiovascular protection [8]. These characteristics have led to the inclusion of ARBs among the first‐line medications recommended in international guidelines for the treatment of hypertension. These guidelines also emphasize evidence‐based indications for ARBs in specific subgroups of hypertensive patients with comorbidities [9].

**FIGURE 1:**
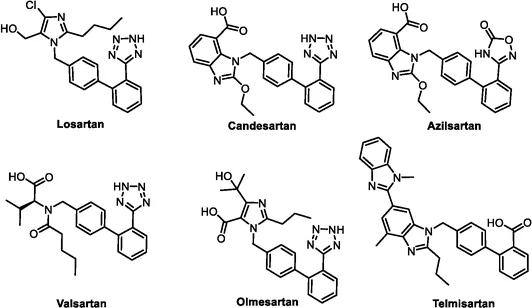
Some ARBs used in current antihypertensive therapy.

The prototype compound of the angiotensin II receptor (AT_1_R) antagonist class is losartan [10]. Other drugs have been developed, such as candesartan, azilsartan, valsartan, olmesartan, and telmisartan, among others (Figure 1). These structures share several similarities, including a bis‐aryl moiety bearing an acid group or equivalent in the ortho position, as well as heteroatoms throughout the structures that enable hydrogen‐bond interactions with the angiotensin II receptor.

Angiotensin II binds primarily to two distinct receptors that share approximately 30% sequence identity: type 1 (AT_1_R) and type 2 (AT_2_R) receptors. Both are part of the renin–angiotensin–aldosterone system and are G protein‐coupled receptors [11]. AT_1_R is located in the plasma membrane of angiotensin II target cells, including vascular smooth muscle cells, renal vasculature, and mesangial cells; adrenal glands; and the brain, whereas AT_2_R is more abundant in the fetus and in newborns [12]. Both receptors are activated by angiotensin II, leading to an increase in cytosolic Ca^2+^ concentrations, triggering protein kinase C stimulation, inhibiting adenylate cyclase, and activating several tyrosine kinases, ultimately resulting in potent vasoconstriction [13].

Oxadiazolones are heterocycles of interest in medicinal chemistry due to their diverse bioactive properties, including glutamate receptor modulation [14], antimicrobial activity [15], and phospholipase A_2_ inhibition [16]. Structurally, these compounds are described as bioisosteres of carboxylic acids, but they tend to be more lipophilic [17]. The 1,2,3‐triazole ring, in turn, consists of a synthetically accessible linker (via click chemistry) that also provides additional hydrogen‐bond acceptor sites relevant for receptor recognition. Based on these characteristics, the present study aimed to identify novel oxadiazolone‐triazole molecular scaffolds as starting points for the development of AT_1_R antagonist candidates. Molecular design was guided by the preservation of key pharmacophoric features commonly found in clinically used angiotensin II receptor blockers, while incorporating a 1,2,4‐oxadiazol‐5‐one containing a side chain to modulate properties and investigate its influence on receptor recognition and structure–activity relationships, providing a rational starting point for further medicinal chemistry optimization.

## 2 Results and Discussion

### 2.1 Chemistry

**FIGURE 2:**
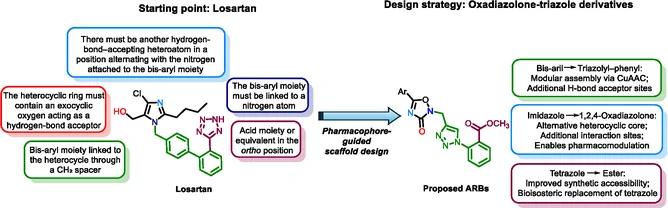
Design of oxadiazolone‐triazole derivatives as AT_1_R receptor antagonist candidates.

The design of the present series was based on the pharmacophoric features described for angiotensin II type 1 receptor (AT_1_R) antagonists established by Belvisi and coworkers [18] through structure–activity relationship (SAR) studies using losartan as the prototype compound. As illustrated in Figure 2, these studies identified the key structural elements associated with AT_1_R recognition, which served as the basis for the design of the new series of compounds.

**FIGURE 3:**
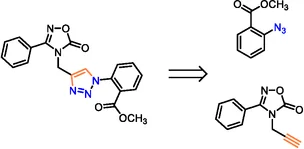
Building blocks for the synthesis of the proposed ARBs.

The objective of this work was not to structurally reproduce losartan, but rather to develop a novel molecular scaffold that preserves pharmacophoric features considered relevant for receptor recognition while providing synthetic flexibility for the preparation of a library of structurally related derivatives. The main structural modification consisted of replacing the biphenyl moiety found in classical AT_1_R antagonists with a triazolyl‐phenyl system. Besides maintaining an aromatic region potentially compatible with molecular recognition, this strategy enabled the use of copper‐catalyzed azide–alkyne cycloaddition (CuAAC) as the key step in the synthetic route [19] (Figure 3). Consequently, the target compounds could be assembled through a semiconvergent strategy, allowing the planned library to be prepared straightforwardly and efficiently with good overall yields. In addition, incorporation of the 1,2,3‐triazole ring introduces additional nitrogen atoms into the scaffold, which may act as hydrogen‐bond acceptors and potentially contribute to molecular recognition.

Another important structural modification was the replacement of losartan's imidazole ring with a 1,2,4‐oxadiazol‐5‐one moiety. The selection of this heterocycle was motivated by its recognized relevance in medicinal chemistry and by its potential as a structural alternative that remains relatively unexplored in AT_1_R antagonist design. The oxadiazolone ring incorporates oxygen and nitrogen atoms that may participate in intermolecular interactions while simultaneously modulating the physicochemical properties of the scaffold relative to those of classical AT_1_R antagonists. Furthermore, the C‐3 side chain provides a versatile site for structural diversification, enabling systematic exploration of structure–activity relationships.

Finally, the tetrazole moiety was replaced by an ester functionality. Although tetrazole is a well‐established bioisostere of carboxylic acids [20] and is widely employed in AT_1_R antagonists, the corresponding ester derivatives proved to be synthetically more accessible within the proposed strategy. Esters, in general, offer advantages over acids, as they can serve as prodrugs, as exemplified by olmesartan medoxomil, an ester prodrug that provides improved olmesartan absorption [21]. Therefore, this modification was introduced to investigate whether this region of the scaffold could tolerate ester substitution without substantially altering the predicted interaction profile of the series. This approach also enabled assessment of the influence of this structural modification on the properties of the new scaffold, providing useful information for future molecular optimization.

**SCHEME 1:**
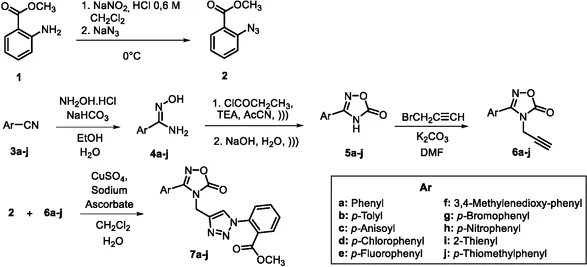
Synthesis of **7a**–**j**.

As mentioned before, our synthetic route is semiconvergent, in which the building blocks are synthesized in parallel. Azide (**2**) is prepared from the methyl ester of anthranilic acid (**1**) via a Sandmeyer reaction. The synthesis of alkynes (**6a**–**j**) begins with the preparation of arylamidoximes (**4a**–**j**) from the corresponding aromatic nitriles (**3a**–**j**) in a hydroalcoholic solution of hydroxylamine. With the amidoximes in hand, the synthesis of 1,2,4‐oxadiazol‐5‐ones (**5a**–**j**) is carried out, starting with the acylation of the amidoxime with ethyl chloroformate, followed by ring closure under basic conditions and sonication. Finally, the 1,2,4‐oxadiazol‐5‐ones are propargylated, affording alkynes **6a**–**j** (Scheme 1).

Once constructed, the building blocks were joined via click chemistry. The methodology described by Sharpless and coworkers was employed [22], in which copper(II) is reduced in situ to copper(I) using a mild reducing agent, sodium ascorbate, in a biphasic medium of dichloromethane and water [23]. The products were purified by column chromatography and obtained in good yields (Scheme 1).

### 2.2 Pharmacological Evaluation

The tests evaluating vascular reactivity were performed in the presence of angiotensin II, so that the effects of the substances designed and synthesized here could be assessed relative to angiotensin II on vasodilation. Typically, ARBs act as competitive inhibitors of AT_1_ receptors [24], so it is expected that compounds **7a**–**j** may displace angiotensin II from receptor binding sites. Angiotensin II activates AT_1_ receptors in vascular smooth muscle cells, activating the Gq protein that, in turn, activates phospholipase C, which breaks membrane phospholipids into the second messengers IP_3_ and diacylglycerol (DAG). These second messengers increase cytoplasmic calcium concentration, leading to vasoconstriction. The vasoconstriction augments vascular resistance and, thus, blood pressure [25]. What is expected, then, when introducing potential AT_1_ receptor inhibitors, is that this vasoconstriction is prevented, thereby reducing blood pressure.

**FIGURE 4:**
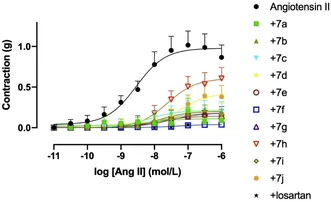
Cumulative concentration‐effect curves for angiotensin II in rat aortic rings in the absence or in the presence of **7a**–**j** (100 μmol/L) or losartan (1 μmol/L). The symbols represent the mean, and the bars represent the standard error of the mean.

It was observed that all synthesized molecules reduced the maximum contraction induced by angiotensin II, except for **7h** (Figure 4). The values of maximum effect and pCE_50_ (negative common logarithm of the half‐maximal effective concentration) are in Table 1. These findings are consistent with the modulation of AT_1_ receptor‐mediated responses, especially for compound **7f**, which is substituted with a methylenedioxyphenyl group.

**TABLE 1:** Maximum effect, pCE_50_, and number of experiments (*n*) for the contraction induced by angiotensin II in rat aortic rings in the absence or in the presence of **7a–j** (100 μmol/L) or losartan (1 μmol/L).

Substance	Maximum effect, g	**pEC**_**50**_	Number of experiments, *n*
**Angiotensin II**	1.041 ± 0.190	8.388 ± 0.192	10
** +7a**	0.086 ± 0.035^a^	7.183 ± 1.386	5
** +7b**	0.220 ± 0.059^a^	7.496 ± 0.312	5
** +7c**	0.218 ± 0.116^a^	8.314 ± 0.198	6
** +7d**	0. 227 ± 0.129^a^	8.466 ± 0.344	6
** +7e**	0.156 ± 0.058^a^	8.394 ± 0.483	5
** +7f**	**0.038 ± 0.017** a	**7.463 ± 0.490**	**5**
** +7g**	0.184 ± 0.075^a^	7.418 ± 0.506	7
** +7h**	0.643 ± 0.135	7.452 ± 0.326	6
** +7i**	0.183 ± 0.063^a^	7.447 ± 0.272	6
** +7j**	0.406 ± 0.148^a^	7.110 ± 0.188	7
**+ Losartan**	0.035 ± 0.016^a^	6.816 ± 1.166	6

*Note:* Data are expressed as mean ± standard error. Bold indicates the most favorable maximum effect among the synthesized compounds.

a *p* < 0.05 compared to the control group (ang II), ANOVA followed by Tukey's.

Regarding the overall results obtained for the series, it can be observed that the strongly deactivating group on the ring, the nitro group (**7h**), is detrimental to activity, as it was the only compound that did not show a vasodilatory effect. The canonical compound of the series, **7a**, showed the second‐best performance in terms of vasodilation. Next, compounds **7e** (fluorine‐substituted) and **7g** (bromine‐substituted) were also quite active, both presenting maximum effects below 0.2 g. This indicates that substitution with electronegative atoms does not improve biological activity. Furthermore, when comparing bromine and fluorine, both electronegative but with significantly different sizes, no substantial differences in activity were observed.

Additionally, the nonclassical bioisosteric replacement of phenyl with thiophene did not improve activity, as compound **7i** (thiophene) showed lower activity than canonical compound **7a**. Among the electron‐donating groups, besides **7f**, which exhibited good activity, compounds **7b** (*p*‐tolyl) and **7c** (*p*‐methoxyphenyl) showed moderate activity, whereas **7j**, containing the *p*‐thiomethylphenyl group, displayed the lowest activity among them. This suggests that replacing oxygen (**7c**) with sulfur (**7j**) is not beneficial.

The compounds developed herein do not contain the classical “sartan” moiety, which consists of a bis‐aryl group ortho‐substituted with an acidic group or a bioisosteric equivalent. Instead, compounds **7a**–**j** feature a 1,2,3‐triazole‐phenyl moiety. The introduction of a 1,2,3‐triazole ring may provide additional binding sites within the receptor due to the presence of three extra nitrogen atoms. From a pharmacological standpoint, the presence of this group appears to be beneficial. This effect may arise either from an increased number of interactions within the active site or simply from its role as an additional aromatic spacer. Therefore, molecular docking studies were performed to further investigate this hypothesis.

It should be noted that all synthesized compounds were evaluated at a single screening concentration (100 μM), whereas losartan was tested at 1 μM. This way, the present experiments were designed to identify compounds capable of modulating the Ang II‐induced vascular response under the experimental conditions employed. They should not be interpreted as providing information on relative potency or pharmacological equivalence with losartan. The vascular reactivity assay used herein should be regarded as an initial biological screening to identify compounds that modulate the vascular response to Ang II, and additional studies will be required to determine potency, receptor selectivity, and the mechanism of action.

### 2.3 Molecular Docking Simulations

Molecular docking was performed to investigate whether the proposed oxadiazolone‐triazole scaffold could be accommodated within the AT_1_ receptor binding pocket and whether the introduced structural modifications would preserve a molecular recognition pattern comparable to that observed for classical AT_1_ receptor antagonists. This way, it can help generate structural hypotheses to aid the interpretation of biological findings and observed structure–activity relationships.

The study was conducted using data reported by Zhang et al. [26], using the AT_1_R receptor structure (PDB code 4YAY) cocrystallized with the antagonist ligand ZD7155. The ligand ZD7155 is a precursor of candesartan and has high affinity for the receptor [26]. The molecular docking assay began with validation of the employed methodology via a redocking simulation. For this validation, some parameters were considered, such as the ligand's ability to reproduce the experimentally observed conformation, the preservation of hydrogen bond interactions with active site residues, and the attainment of RMSD values ≤2.0 Å.

**FIGURE 5:**
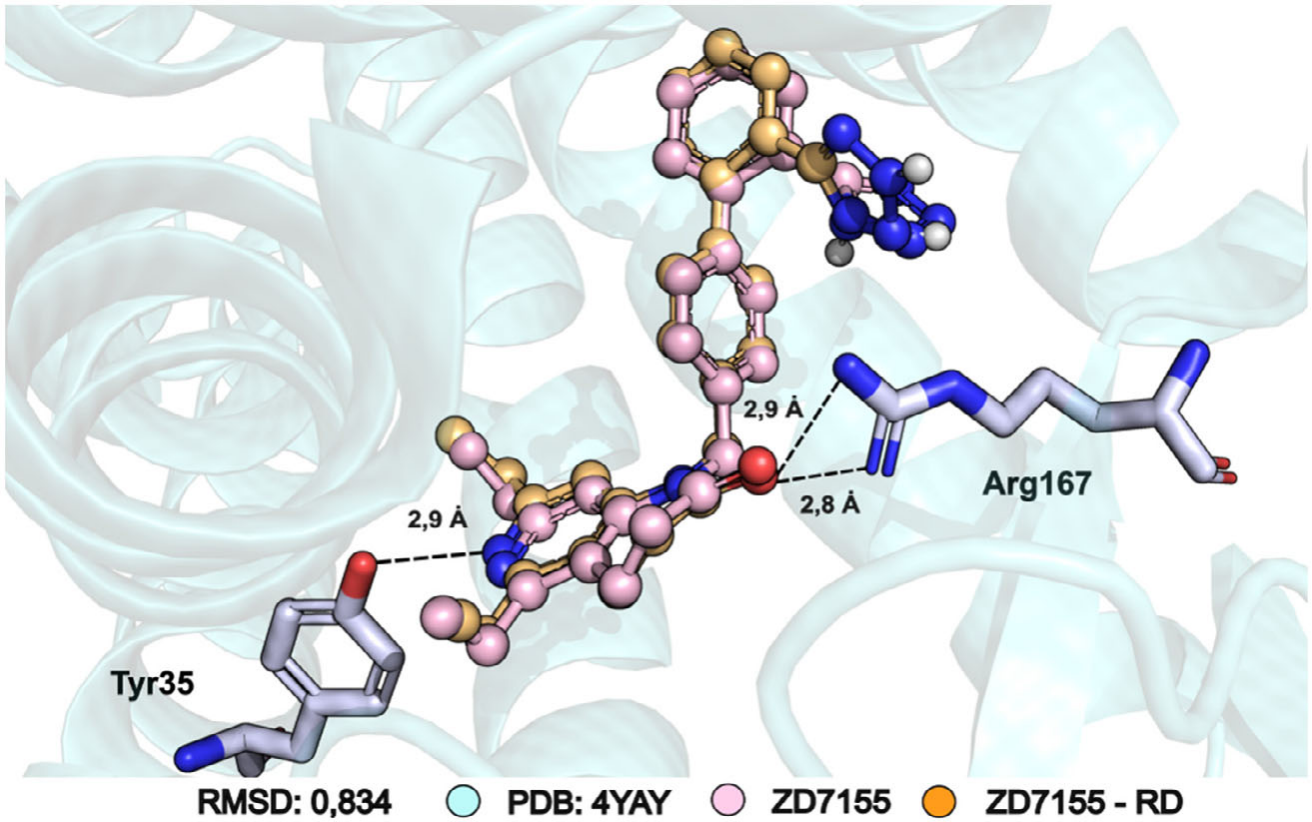
Conformation of the ZD7155 ligand (yellow) after redocking compared to the original conformation (pink). The residues Arg167 and Tyr35 are highlighted, showing the hydrogen bonds with the 4YAY crystal structure (green).

It was observed that ZD7155‐RD (yellow) established interactions with the residues Arg167 and Tyr35 (Figure 5), as also observed for the cocrystallized ligand ZD7155 (pink). Visual analysis revealed good conformational overlap between the compounds, suggesting that the proposed ligand reproduced a conformation similar to that of the cocrystallized ligand. Corroborating this observation, the RMSD of 0.834 Å falls within acceptable limits, reinforcing the reliability of the obtained binding pose.

After validation of the method, docking simulations were initiated for the compounds of the series (**7a**–**j**). One hundred runs were performed for each structure, and the three best conformations were selected for analysis. In general, the evaluation focused on the scores for each ligand and the hydrogen‐bond interactions established (number in parentheses), as shown in Table 2. The reference ligand ZD7155 had a score of 99.60, while the evaluated compounds ranged from 76 to 86. In addition to the reference ligand ZD7155 and the synthesized compounds, molecular docking simulations were also performed for losartan. For losartan, a docking score of 93.39 was obtained. The predicted binding poses of the synthesized compounds were compatible with the architecture of the AT_1_R binding pocket. Despite the structural modifications introduced relative to classical antagonists, the docking results suggest that the proposed oxadiazolone‐triazole scaffold can be accommodated within the receptor binding cavity while preserving a binding orientation compatible with that predicted for reference AT_1_R ligands.

**TABLE 2:** Molecular docking score and main interactions observed for **7a–j**.

Molecule	Score	**Hydrogen‐bonding interactions** a
**ZD7155**	99.60	(2) Arg167, (1) Tyr35
**7a**	80.22	(1) Tyr87, (3) Arg167
**7b**	81.18	(1) Arg167
**7c**	84.00	(1) Arg167, (1) Tyr35
**7d**	81.64	(1) Tyr87, (3) Arg167
**7e**	81.45	(1) Tyr87, (3) Arg167
**7f**	82.46	(1) Tyr87, (1) Arg167, (1) Tyr92
**7g**	82.09	(1) Arg167, (1) Tyr92
**7h**	82.40	(1) Tyr87, (3) Arg167, (1) Tyr35
**7i**	76.13	(1) Arg167
**7j**	86.37	(1) Tyr92, (3) Arg167
**Losartan**	93.39	(2) Arg167, (2) Tyr87

a Numbers in parentheses refer to the number of hydrogen‐bond interactions.

**FIGURE 6:**
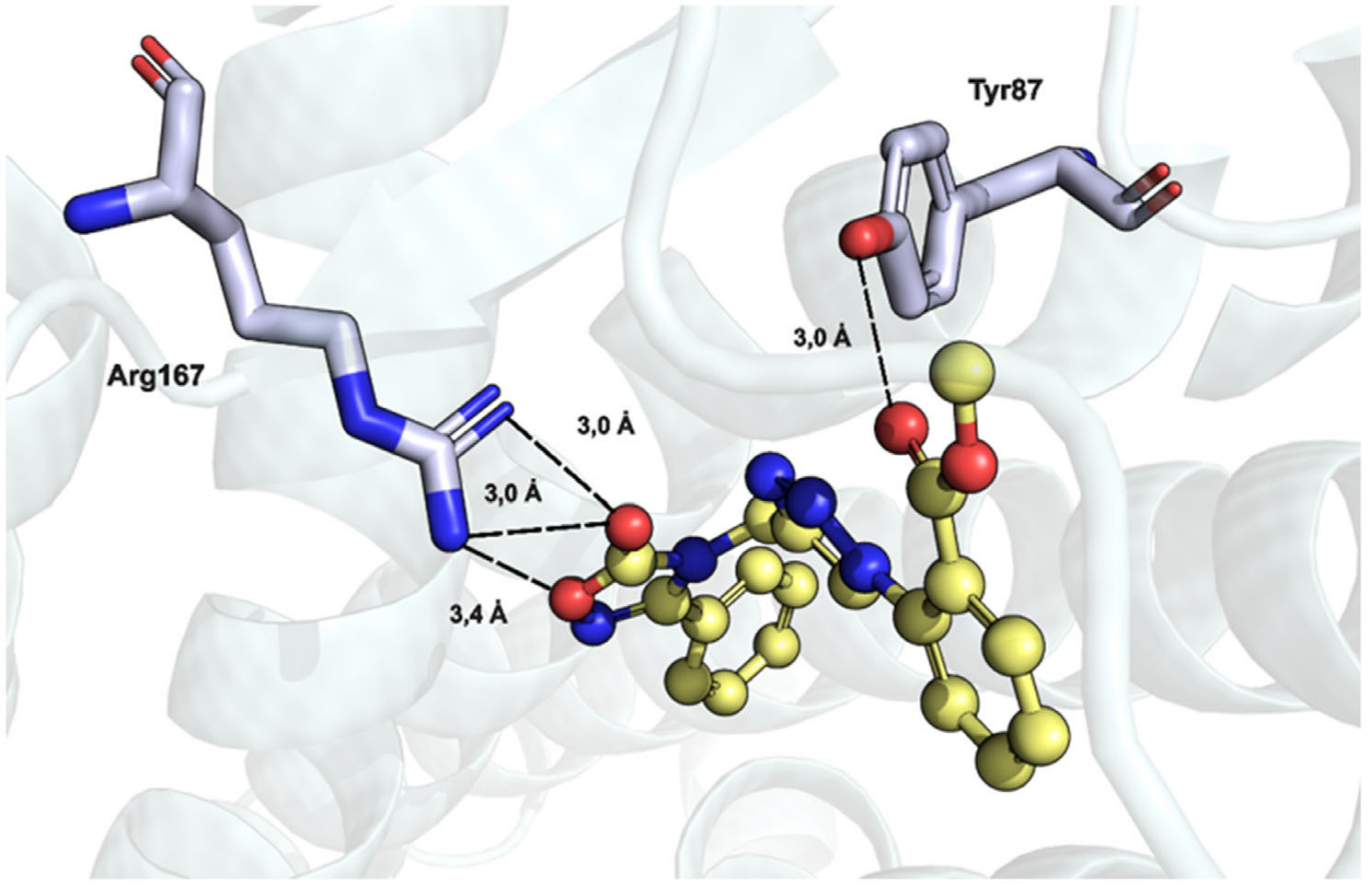
Predicted binding pose of compound **7a** (yellow) in the AT_1_R (PDB ID: 4YAY), highlighting the hydrogen bonds formed with residues Arg167 and Tyr87.

Analysis of the docking poses revealed a recurring interaction pattern across the entire series. All compounds established at least one predicted hydrogen bond with the Arg167 residue, whereas additional interactions with Tyr35, Tyr87, and Tyr92 were observed for selected derivatives, depending on the nature of the aromatic substituents. Compound **7a**, the canonical representative of the series (Figure 6), illustrates this general binding mode. Ligands ZD7155 and losartan were predicted to establish two hydrogen bonds with Arg167, whereas the synthesized compounds displayed only a single interaction with this residue.

For most derivatives, the interaction with Arg167 involved the carbonyl oxygen of the 1,2,4‐oxadiazol‐5‐one ring, whereas interactions with Tyr87 occurred predominantly through the carbonyl oxygen of the ester functionality. In contrast, interactions with Tyr35 and Tyr92 were not observed for all compounds, suggesting that pharmacomodulation can influence the scaffold's orientation within the binding pocket.

Comparison of the experimental results with the predicted docking poses revealed an interesting trend: although several interactions are predicted, the altered orientation mainly enables the triazole‐Tyr87 interaction for compounds **7a, 7d**, **7e**, and **7f.** This observation does not establish a causal relationship between the predicted interaction and the observed biological activity; it suggests that this residue may be a relevant site for future structural optimization studies.

**FIGURE 7:**
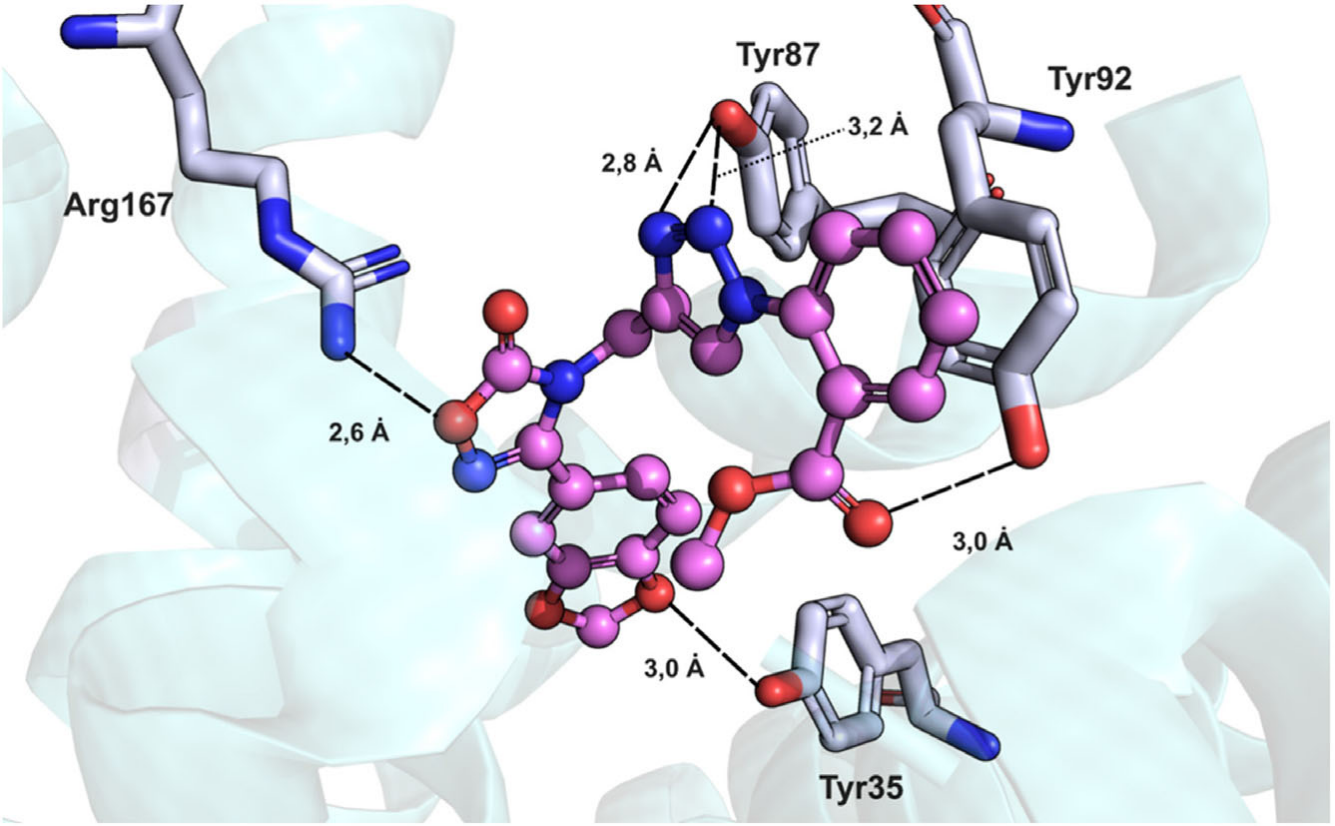
Interaction of compound **7f** (lilac) with AT_1_R (PDB ID: 4YAY), highlighting the hydrogen bonds formed with residues Tyr87, Tyr35, Arg167, and Tyr92.

Among the evaluated compounds, derivative **7f** deserves particular attention because it exhibited the highest activity in the biological assays while simultaneously adopting a slightly different predicted binding mode compared with the other members of the series (Figure 7). In this derivative, the introduction of the methylenedioxy group altered the scaffold's orientation, enabling a predicted interaction between the 1,2,3‐triazole ring and the Tyr87 residue. In the remaining compounds, by contrast, interaction with Arg167 occurred predominantly through the 1,2,4‐oxadiazol‐5‐one ring.

An additional aspect investigated in this study was the possible role of the 1,2,3‐triazole ring in receptor recognition. The results suggest that this heterocycle primarily functions as a structural component of the scaffold, since no direct interactions between its nitrogen atoms and the receptor were predicted for most compounds. Once again, compound **7f** stood out from the remaining derivatives, as the triazole ring was predicted to interact directly with Tyr87. These findings suggest that the contribution of the triazole moiety to molecular recognition may depend on the conformation adopted by each derivative within the binding pocket.

Docking studies indicate that the proposed oxadiazolone‐triazole scaffold can be accommodated within the AT_1_ receptor binding pocket while preserving a molecular recognition pattern comparable to that of the reference ligands. Thus, computational results provide a plausible structural rationale for interpreting the experimental findings and may assist future molecular optimization efforts. However, the proposed interactions should be regarded as hypotheses generated by molecular modeling rather than as experimental evidence of receptor binding or of the compounds’ mechanism of action.

### 2.4 In Silico Pharmacokinetic Evaluation

As compound **7f** exhibited the most promising biological profile in the vascular reactivity assays, its physicochemical and pharmacokinetic properties were evaluated in silico using the SwissADME server [27, 28]. SwissADME is a free online tool that calculates physicochemical properties and predicts pharmacokinetic parameters (Absorption, Distribution, Metabolism, and Excretion) for small molecules [28]. In addition to **7f**, the parameters of compound **7a**, the canonical molecule of this series, were also calculated. These analyses were intended to provide a preliminary assessment of properties relevant to the future development of this scaffold. They should be interpreted as computational predictions rather than experimentally determined pharmacokinetic parameters (Table 3).

**TABLE 3:** Some calculated physicochemical and pharmacokinetic parameters for **7a** and **7f**.

Molecule	**log *P***_**(o/w)**_ a	**Solubility, mg/mL** b	GI absorption	BBB permeant	**Drug‐likeness** c	Bioavailability score
**7a**	2.42	0.0049	High	No	Yes	0.55
**7f**	2.23	0.0043	High	No	Yes	0.55

a Measured as XlogP3.

b Calculated from Log*S* (ESOL).

c Measured from Lipinski's rule of five filter. No violations of this rule were encountered for the tested compounds.

The predicted log *P* values fell within the range commonly observed for small bioactive molecules. As expected, compound **7f**, which contains a greater number of heteroatoms, exhibited a lower predicted log *P* value than **7a**, reflecting its higher estimated polarity.

Regarding aqueous solubility, SwissADME predicted low solubility for both compounds, particularly for **7f**. At first glance, this result may appear inconsistent with the concentrations employed in the pharmacological assays. However, it should be emphasized that the predicted solubility is derived solely from the molecular structure and does not necessarily reflect the experimental conditions used in biological assays, where factors such as solvents, formulation vehicles, and sample preparation may substantially influence compound availability. This apparent discrepancy should be interpreted with caution and clarified only through experimental solubility measurements.

Despite the predicted low aqueous solubility, SwissADME classified both compounds as having high gastrointestinal absorption. Nevertheless, this classification represents a computational estimate based on molecular descriptors and should not be interpreted as evidence of high in vivo absorption [29, 30]. Likewise, the calculated bioavailability score (0.55) is an empirical indicator commonly used in early‐stage drug discovery and does not reflect the true oral bioavailability. The simulations also predicted that neither compound is likely to cross the blood–brain barrier. Given that the AT_1_R is predominantly a peripheral therapeutic target, this characteristic may be considered favorable from a pharmacological perspective [31].

The drug‐likeness assessment demonstrated that both compounds **7a** and **7f** comply with Lipinski's Rule of Five and other medicinal chemistry filters implemented in SwissADME. These findings suggest that both compounds possess structural features frequently associated with orally active bioactive molecules [32], although compliance with these empirical rules does not guarantee successful pharmaceutical development. Similarly, no PAINS alerts were identified, indicating the absence of substructures commonly associated with assay interference [33].

In general, all parameters discussed in this section are based on computational predictions and should be regarded solely as preliminary indicators. Additional experimental studies are required to confirm the aqueous solubility, intestinal absorption, metabolic stability, and other pharmacokinetic properties of compounds **7a** and **7f**.

## 3 Conclusion

In this study, a novel series of 1,2,4‐oxadiazol‐5‐one derivatives incorporating a 1,2,3‐triazole‐phenyl moiety was designed and synthesized via a semiconvergent strategy using click chemistry. Despite lacking the classical sartan pharmacophore, the compounds showed biological activity in vascular reactivity assays, with most derivatives effectively reducing angiotensin II‐induced contraction, consistent with modulation of AT_1_ receptor‐mediated responses. Compound **7f** emerged as the most promising hit within the synthesized series and represents a suitable starting point for further medicinal chemistry optimization and detailed pharmacological characterization.

The results also indicated that electron‐donating substituents improve biological activity, whereas strongly electron‐withdrawing groups, such as nitro, reduce the inhibitory effect. Molecular docking analyses provided a plausible structural rationale for interpreting the experimental findings, suggesting that the compounds establish interactions with key residues, including Arg167 and Tyr87, which have been implicated in ligand recognition by the AT_1_R. The docking analyses suggest that the 1,2,3‐triazole ring may contribute to the predicted molecular recognition, either by enabling additional interactions within the binding site, as observed for compound **7f**, or by acting as an aromatic spacer in the remaining derivatives.

In silico ADME analysis predicted physicochemical and pharmacokinetic properties compatible with those of early‐stage drug candidates, including compliance with established drug‐likeness criteria. Taken together, these findings identify the oxadiazolone‐triazole scaffold as a useful starting point for further medicinal chemistry optimization toward candidate AT_1_ receptor antagonists.

## 4 Experimental Section

### 4.1 Chemistry

The commercial reagents and solvents used in this work were not subjected to any additional purification steps, but triethylamine, acetonitrile, and dimethylformamide were distilled utilizing the method of Armarego and Chai [34]. In general, all reagents and solvents used in the study were obtained from Sigma‐Aldrich (USA) and Dinâmica (Brazil). The reactions were monitored by thin‐layer chromatography (TLC) using Sigma‐Aldrich (USA) silica plates impregnated with the fluorescent indicator F_254_, which is sensitive to ultraviolet light at 254 nm. The final substances were purified by column chromatography using silica gel from Sigma‐Aldrich (USA) with particle sizes ranging from 0.063 to 0.200 mm (70‐230 mesh). The melting points of the substances were determined using the Electro‐thermal Mel‐Temp equipment (Bibby Scientific, UK) and were not corrected.

The ^1^H and ^13^C NMR spectra were carried out at the Analytical Centre (UFPE) using the Varian (USA) URMNS 400 MHz spectrometer. NMR data processing was performed using the MestReNova program, version 12, owned by Mestrelab Research S.L. (Spain). For each analysis, 30 mg of each substance was used, and the solvents employed varied according to the solubility of the samples. High‐resolution mass spectra were acquired by direct infusion of the samples using a Q‐Exactive mass spectrometer (Thermo Scientific, USA) with an H‐ESI source.

The aryl‐amidoximes (**4a**–**j**) were synthesized based on the methodology of Srivastava et al. [35]. 1,2,4‐Oxadiazole‐5‐ones (**5a**–**j**) and their respective propargyl derivatives (**6a**–**j**) were synthesized following the procedure described by Ferreira and coworkers [36].

#### 4.1.1 General Procedure for the Alkyne‐Azide “Click” Reaction Between 3‐Aryl‐4‐(propynyl)‐1,2,4‐oxadiazol‐5‐ones and 2‐Azidobenzoic Acid Methyl Ester (7a–j)

In a 50‐mL round‐bottom flask, 0.60 mmol of the alkyne derivative of the corresponding 1,2,4‐oxadiazol‐5‐one and 0.66 mmol of methyl 2‐azidobenzoate (117 mg, 1.1 eq) were dissolved in 10 mL of dichloromethane. To this solution, a mixture of copper sulfate pentahydrate (0.09 mmol, 0.15 eq, 23 mg) and sodium ascorbate (0.27 mmol, 0.45 eq, 54 mg), suspended in 10 mL of water, was added. The biphasic system reacted for 24 h. Upon completion of the reaction, the mixture was acidified with concentrated HCl and extracted with dichloromethane (2 × 20 mL). The organic phases were dried, the solvent was evaporated, and the residue was submitted to column chromatography using hexane and ethyl acetate as the mobile phase (7:3, then 1:1, v/v).

Methyl 2‐(4‐{[5‐oxo‐3‐phenyl‐1,2,4‐oxadiazol‐4(5*H*)‐yl]methyl}‐1*H*‐1,2,3‐triazol‐1‐yl)benzoate (**7a**). Yield: 54%; m.p.: 86–89 °C; Rf: 0.39 (1:1 hexane/ethyl acetate, v/v). ^1^H NMR (CDCl_3_) δ (ppm): 3.72 (s, 3H, CH_3_); 5.03 (s, 2H, CH_2_); 7.51 (d, 1H, *J* = 8.0 Hz, H_Ar_); 7.74–7.58 (m, 5H, H_Ar_); 7.95 (d, 1H, *J* = 8.0 Hz, H_Ar_); 8.05 (d, 1H, *J* = 8.0 Hz, H_Ar_); 8.09 (s, 1H, H_triazole_). ^13^C RMN (CDCl_3_) δ (ppm): 38.4; 52.7; 122;8; 126.2; 127.0; 127.3; 129.0; 129.5; 130.4; 131.5; 132.4; 133.0; 135.9; 140.7; 158.8; 159.5; 165.2. HRMS calcd for C_19_H_16_N_5_O_4_ [M + H]^+^ : 378.1202. Found: 378.1194.

Methyl 2‐(4‐{[5‐oxo‐3‐*p*‐tolyl‐1,2,4‐oxadiazol‐4(5*H*)‐yl]methyl}‐1*H*‐1,2,3‐triazol‐1‐yl)benzoate (**7b**). Yield: 83%; m.p.: 90–93 °C; Rf: 0.50 (1:1 hexane/ethyl acetate, v/v). ^1^H NMR (CDCl_3_) δ (ppm): 3.47 (s, 3H, CH_3_); 3.72 (s, 3H, CH_3_); 5.01 (s, 2H, CH_2_); 7.41 (d, 1H, *J* = 8.0 Hz; H_Ar_); 7.50 (d,1H, *J* = 8.0 Hz, H_Ar_); 7.73–7.62 (m, 4H, H_Ar_); 7.81 (d, 1H, *J* = 8.0 Hz; H_Ar_); 8.04 (d, 1H, *J* = 8.0 Hz; H_Ar_); 8.09 (s, 1H, H_triazole_). ^13^C RMN (CDCl_3_) δ (ppm): 21.6; 38.9; 52.6; 119.8; 126.2; 126.9; 127.3; 128.8; 130.3; 131.5; 132.9; 135.8; 140.8; 142.9; 158.8; 159.6; 165.3. HRMS calcd for C_20_H_18_N_5_O_4_ [M + H]^+^: 392.1359. Found: 392.1354.

Methyl 2‐(4‐{[5‐oxo‐3‐*p*‐anisoyl‐1,2,4‐oxadiazol‐4(5*H*)‐yl]methyl}‐1*H*‐1,2,3‐triazol‐1‐yl)benzoate (**7c**). Yield: 81%; m.p.: 92–95 °C; Rf: 0.39 (1:1 hexane/ethyl acetate, v/v). ^1^H NMR (CDCl_3_) δ (ppm): 3.72 (s, 3H, CH_3_); 3.90 (s, 3H, CH_3_); 5.02 (s, 2H, CH_2_); 7.11 (d, 2H, *J* = 8.0 Hz, H_Ar_); 7.51 (d, 1H, *J* = 8.0 Hz, H_Ar_); 7.76–7.60 (m, 2H, H_Ar_); 7.90 (d, 2H, *J* = 8.0 Hz, H_Ar_); 8.05 (d, 1H, *J* = 8.0 Hz, H_Ar_); 8.10 (s. 1H, H_triazole_). ^13^C RMN (CDCl_3_) δ (ppm): 38.4; 52.6; 55.5; 114.7; 114.9; 126.3; 126.9; 127.3; 130.3; 130.6; 131.5; 132.9; 135.8; 140.8; 158.6; 159.6; 162.7; 165.2. HRMS calcd for C_20_H_18_N_5_O_5_ [M + H]^+^ : 408.1308. Found: 408.1305.

Methyl 2‐(4‐{[5‐oxo‐3‐*p*‐chlorophenyl‐1,2,4‐oxadiazol‐4(5*H*)‐yl]methyl}‐1*H*‐1,2,3‐triazol‐1‐yl)benzoate (**7d**). Yield: 46%; m.p.: 85–87 °C; Rf: 0.53 (1:1 hexane/ethyl acetate, v/v). ^1^H NMR (CDCl_3_) δ (ppm): 3.75 (s,3H,CH_3_); 5.01 (s,2H,CH_2_); 7.52 (d,1H, *J* = 8.0 Hz, H_Ar_); 7.75–7.59 (m, 4H, H_Ar_); 7.96 (d, 2H, *J* = 8.0 Hz, H_ar_); 8.07 (d, 1H, *J* = 8.0 Hz, H_ar_); 8.10 (s, 1H, H_triazole_). ^13^C RMN (CDCl_3_) δ (ppm): 38.4; 52.7; 121.2; 126.3; 127.0; 127.2; 129.9; 130.4; 130.4; 131.5; 133.0; 135.8; 138.9; 140.5; 158.0; 159.3; 165.1. HRMS calcd for C_20_H_16_ClN_5_O_6_ [M + H]^+^ : 456.0711. Found: 456.0721.

Methyl 2‐(4‐{[5‐oxo‐3‐*p*‐fluorophenyl‐1,2,4‐oxadiazol‐4(5*H*)‐yl]methyl}‐1*H*‐1,2,3‐triazol‐1‐yl)benzoate (**7e**). Yield: 82%; m.p.: 85–87 °C; Rf: 0.53 (1:1 hexane/ethyl acetate, v/v). ^1^H NMR (CDCl_3_) δ (ppm): 3.75 (s, 3H, CH_3_); 5.01 (s, 2H, CH_2_); 7.36–7.28 (m, 2H, H_Ar_); 7.52 (d, 1H, *J* = 8.0 Hz, H_Ar_); 7.76–7.63 (m, 2H, H_Ar_); 8.10–7.99 (m, 3H, H_Ar_); 8.11 (s, 1H, H_triazole_). ^13^C RMN (CDCl_3_) δ (ppm): 30.9; 38.3; 52.7; 116.8; 117.0; 118.9; 118.9; 126.3; 127.0; 127.2; 130.4; 131.5; 131.6; 133.0; 135.9; 140.5; 158.0; 159.4; 163.8; 166.3. HRMS calcd for C_19_H_15_N_5_O_4_ F [M + H]^+^ : 396.1108. Found: 396.1105.

Methyl 2‐(4‐{[5‐oxo‐3‐(3,4‐methylenedioxyphenyl)‐1,2,4‐oxadiazol‐4(5*H*)‐yl]methyl}‐1*H*‐1,2,3‐triazol‐1‐yl)benzoate (**7f**). Yield: 69%; m.p.: 130–133 °C; Rf: 0.39 (1:1 hexane/ethyl acetate, v/v). ^1^H NMR (CDCl_3_) δ (ppm): 3.73 (s, 3H, CH_3_); 5.02 (s, 2H, CH_2_); 6.10(s, 2H, CH_2_); 7.01 (d, 1H, *J* = 8.0 Hz, H_Ar_); 7.40 (s, 1H, H_Ar_); 7.50 (d, 2H, *J* = 8.0 Hz, H_Ar_); 7.76–7.50 (m, 2H, H_Ar_); 8.05 (d, 1H, *J* = 8.0 Hz, H_Ar_); 8.08 (s, 1H, H_triazole_).^13^C RMN (CDCl_3_) δ (ppm): 38.4; 52.6; 102.0; 108.9; 109.3; 116.0; 124.1; 126.2; 126.9; 127.3; 130.3; 131.4; 132.9; 135.8; 140.7; 148.6; 151.1; 158.4; 159.5; 165.2. HRMS calcd for C_20_H_16_N_5_O_6_ [M + H]^+^ : 422.1101. Found: 422.1096.

Methyl 2‐(4‐{[5‐oxo‐3‐*p*‐bromophenyl‐1,2,4‐oxadiazol‐4(5*H*)‐yl]methyl}‐1*H*‐1,2,3‐triazol‐1‐yl)benzoate (**7g**). Yield: 47%; m.p.: 90–93 °C; Rf: 0.57 (1:1 hexane/ethyl acetate, v/v). ^1^H NMR (CDCl_3_) δ (ppm): 3.74 (s, 3H, CH_3_); 5.01 (s, 2H, CH_2_); 7.51 (d, 1H, *J* = 7.8 Hz, H_Ar_); 7.81–7.61 (m, 4H, H_Ar_); 7.88 (d, 2H, 7.8 Hz, H_Ar_); 8.07 (d, 1H, *J* = 8.0 Hz, H_Ar_); 8.09 (s,1H, H_triazole_). ^13^C RMN (CDCl_3_) δ (ppm): 38.4; 52.7; 121.7; 126.3; 127.0; 127.2; 127.4; 130.4; 130.5; 130.7; 131.5; 132,9; 132,9; 135,8; 140,4; 158,1; 159,4; 165,1. HRMS calcd for C_19_H_15_N_5_O_4_Br [M + H]^+^ : 456.0307. Found: 456.0308.

Methyl 2‐(4‐{[5‐oxo‐3‐*p*‐nitrophenyl‐1,2,4‐oxadiazol‐4(5*H*)‐yl]methyl}‐1*H*‐1,2,3‐triazol‐1‐yl)benzoate (**7h**). Yield: 70%; m.p.: 129–131 °C; Rf: 0.46 (1:1 hexane/ethyl acetate, v/v). ^1^H NMR (CDCl_3_) δ (ppm): 3.74 (s, 3H, CH_3_); 5.01 (s, 2H, CH_2_); 7.51 (d, 1H, *J* = 8.0 Hz, H_Ar_); 7.81–7,61 (m, 4H, H_Ar_); 7.88 (d, 2H, *J* = 8.0 Hz, H_Ar_); 8.07 (d, 1H, *J* = 8.0 Hz, H_Ar_); 8.09 (d, 1H, H_triazole_).^13^C RMN (CDCl_3_) δ (ppm): 38.6; 52.7; 124.6; 126.3; 127.1; 127.2; 128.7; 130.5; 130.5; 131.5; 133.1; 135.8; 140.1; 150.1; 157.2; 159.06; 165.0. HRMS calcd for C_19_H_15_N_6_O_6_ [M + H]^+^: 423.1053. Found: 423.1046.

Methyl 2‐(4‐{[5‐oxo‐3‐(2‐thienyl)‐1,2,4‐oxadiazol‐4(5*H*)‐yl]methyl}‐1*H*‐1,2,3‐triazol‐1‐yl)benzoate (**7i**). Yield: 75%; m.p.: 120–122 °C; Rf: 0.39 (1:1 hexane/ethyl acetate, v/v). ^1^H NMR (CDCl_3_) δ (ppm): 3.71 (s, 3H, CH_3_); 5.17 (s, 2H, CH_2_) 7.34–7.28 (m, 1H, H_Ar_); 7.51 (d, 2H, *J* = 8.1 Hz, H_Ar_); 7.74–7.60 (m, 3H, H_Ar_); 8.06 (d, 1H, *J* = 8.1 Hz, H_Ar_); 8.08 (s, 1H, H_triazole_); 8.23 (d, 1H, *J* = 8.0 Hz, H_Ar_). ^13^C RMN (CDCl_3_) δ (ppm): 52.7; 122.4; 125.9; 126.9; 127.3; 128.8; 130.3; 130.9; 131.5; 132.0; 132.9; 135.8; 140.82; 153.8; 159.3; 165.2. HRMS calcd for C_17_H_13_N_5_O_4_SNa [M + Na]^+^: 406.0586. Found: 406.0580.

Methyl 2‐(4‐{[5‐oxo‐3‐*p*‐thiomethylphenyl‐1,2,4‐oxadiazol‐4(5*H*)‐yl]methyl}‐1*H*‐1,2,3‐triazol‐1‐yl)benzoate (**7j**). Yield: 79%; m.p.: 123–125 °C; Rf: 0.46 (1:1 hexane/ethyl acetate, v/v). ^1^H NMR (CDCl_3_) δ (ppm): 2.56 (s, 3H, CH_3_); 3.73 (s, 3H, CH_3_); 5.02 (s, 2H, CH_2_); 7.44 (d, 2H, *J* = 8.1 Hz, H_Ar_); 7.52 (d, 1H, *J* = 8.0 Hz, H_Ar_); 7.70–7.61 (m, 1H, H_Ar_); 7.77–7.69 (m, 1H, H_Ar_); 7.95–7.84 (m, 2H, H_Ar_); 8.07 (d, 1H, *J* = 8.1 Hz, H_Ar_); 8.10 (s, 1H, H_triazole_). ^13^C RMN (CDCl_3_) δ (ppm): 14.8; 38.4; 52.6; 118.5; 124.7; 126.1; 126.3; 126.9; 127.3; 129.1; 130.1; 130.3; 130.5; 132.5; 135.8; 140.7; 146.0; 158.4; 158.5; 165.2. HRMS calcd for C_20_H_18_N_5_O_4_S [M + H]^+^: 424.1079. Found: 424.1066.

### 4.2 Pharmacological Evaluation

#### 4.2.1 Animals

Male Wistar rats (*Rattus novergicus* var albinus), 2–3 months old (180–250 g), were obtained from the Bioterium of the Department of Physiology and Pharmacology of UFPE and maintained in the Bioterium of the Academic Center of Vitória of UFPE. The animals were maintained in a controlled light (12/12‐h cycle) and temperature (22 ± 2 °C), fed ad libitum with standard rat chow (Nuvilab), and had free access to tap water. The protocols were approved by the Ethics Committee for Animal Use of UFPE (CEUA/UFPE 0044/2023).

#### 4.2.2 Vascular Reactivity Studies

The animals were anesthetized with isoflurane and euthanized by decapitation. Their thoracic aortas were isolated, cleaned of connective tissue and fat, and cut into 3–5 mm long rings. The rings were mechanically rubbed to remove endothelium and mounted between two metal hooks, with one connected to a force transducer for recording isometric tension and the other fixed to the chamber. Responses were recorded on an acquisition system (AVS Projetos, Brazil, or AD Instruments, Australia). The organ chambers contained 10 mL of Krebs solution (in mmol/L: NaCl 130; KCl 4.7; KH_2_PO_4_ 1.2; CaCl_2_ 1.6; MgSO_4_ 1.2; NaHCO_3_ 14.9 and glucose 5.5) at pH 7.4, bubbled with a carbogenic mixture (95% O_2_ and 5% CO_2_), at 37 °C. The rings were maintained at 1.5 g basal tension for 1 h, with washes every 15 min to stabilize the response. After stabilization, the viability of the rings was assessed by the contraction induced by phenylephrine (0.1 μmol/L). To assess endothelial removal, acetylcholine (1 µmol/L) was added to the chamber when the phenylephrine‐induced contraction reached a plateau. The percentage of relaxation induced by acetylcholine was calculated relative to the phenylephrine contraction. Rings were considered endothelium‐denuded if the acetylcholine‐induced relaxation was less than 10%. The rings were then washed.

Cumulative concentration‐effect curves for angiotensin II were then constructed in the absence or in the presence of **7a**–**j** (100 μmol/L) or losartan (angiotensin II AT_1_ receptor antagonist, positive control, 1 μmol/L). Two pharmacological parameters were calculated from the curves: Maximum effect, the maximal effect induced by angiotensin II in each condition, and pEC50, the concentration of angiotensin II that induced 50% of the maximum effect.

#### 4.2.3 Analysis

The pEC_50_ (negative logarithm of the half‐maximal effective concentration) was calculated by using a nonlinear regression (curve fit) on concentration–effect curve data. Data are expressed as mean ± standard error (S.E.M.). The statistical differences among the groups were analyzed using one‐way analysis of variance (ANOVA), followed by Tukey's post hoc test. The significance level in all cases was *p* < 0.05. All analyses were performed using GraphPad Prism (version 9.0).

### 4.3 Computational Methods

#### 4.3.1 Molecular Docking Simulations

To evaluate the binding mode of the compounds in the series (**7a**–**j**) toward the AT_1_R receptor, whose crystallographic structure was obtained from the Protein Data Bank (PDB) under the code 4YAY, in silico simulations were carried out through molecular docking studies. These simulations aimed not only to guide future structural modifications of the series compounds but also to analyze, at the molecular level, the main interactions involved in ligand–receptor recognition, which may be associated with the mechanism of action of the candidate angiotensin II type 1 receptor blockers (ARBs). The GOLD Suite software (version 5.3), developed by the Cambridge Crystallographic Data Centre [37], was used to perform docking simulations.

The selection of the 4YAY structure was based on the work of Zhang et al. [26], who investigated the binding mode of different ARBs to AT_1_R. In this structure, the cocrystallized ligand is ZD7155, a high‐affinity AT_1_ receptor antagonist and a structural precursor of the antihypertensive drug candesartan, making it particularly suitable for comparative studies and validation of the docking protocol.

Before starting the molecular docking simulations, the crystallographic structure of the AT_1_R receptor (4YAY) and the ligands of the series (**7a–j**) were prepared. The macromolecule was processed using the Protein Preparation Wizard tool in the Hermes software (GOLD Suite, version 5.3), following the program's recommended parameters. The procedure included adding hydrogen atoms, removing nonstructural water molecules, and excluding the cocrystallized ligand (ZD7155), keeping only the protein for subsequent simulations. The final file was saved in .pdb format. The binding site was defined based on the selection of the oxygen atom (OH) of the Tyr35 residue, ensuring proper delimitation of the interaction cavity. The coordinates are −19.77199935913086 (*x*), 16.652000427246094 (*y*), and 43.04199981689453 (*z*). The CHEMPLP scoring function was selected for the study.

The three‐dimensional structures of the compounds were built using Chem3D Pro (version 12.0) and exported in .mol2 format. Subsequently, energy minimization was performed using Avogadro software (version 1.2.0) [38], employing the Universal Force Field (UFF) to obtain energetically stable geometries prior to the simulations.

To validate the docking protocol, a redocking procedure of the cocrystallized ligand (ZD7155) into the active site of AT_1_R was carried out. The quality of the reproduced experimental binding mode was evaluated by calculating the root mean square deviation (RMSD) between the experimental and predicted poses; values below 2.0 Å were considered satisfactory. After completing this step, molecular docking (MD) simulations were initiated, with a maximum of 100 runs per designed structure. The simulations lasted an average of 24 h, allowing extraction of the three best‐ranked solutions for each ligand. Finally, ligand–receptor interactions were visualized and analyzed using PyMOL (v.2.5.3) [39] and BIOVIA Discovery Studio Visualizer [40].

#### 4.3.2 Theoretical ADME and Drug‐Likeness Prediction

The in silico ADME screening and drug‐likeness evaluation were performed using the free web tool SwissADME, developed by the Swiss Institute of Bioinformatics and freely available at www.swissadme.ch [27, 28]. 2D structures were imported into the site's interface in canonical Simplified Molecular Input Line Entry System (SMILES) format, and ADME properties were generated.

## Funding

This work was supported by the Fundação de Amparo à Ciência e Tecnologia do Estado de Pernambuco (APQ‐1003.06/22) and Universidade Federal de Pernambuco (Editais Propesqi 5/2024 and 6/2024).

## Conflicts of Interest

The authors declare no conflicts of interest.

## Supporting information

Supplementary Material

## Data Availability

The data that support the findings of this study are available from the corresponding author upon reasonable request.

## References

[1] WHO , “Global Report on Hypertension 2025: High Stakes ‐ Turning Evidence Into Action,” World Health Organization 01 (2025): 1–325.

[2] M. Almutairi , A. A. Almutairi , and A. M. Alodhialah , “The Influence of Lifestyle Modifications on Cardiovascular Outcomes in Older Adults: Findings From a Cross‐Sectional Study,” Life 15 (2025): 87, 10.3390/life15010087. 39860027 PMC11767055

[3] A. V. Chobanian , G. L. Bakris , H. R. Black , et al., “The Seventh Report of the Joint National Committee on Prevention, Detection, Evaluation, and Treatment of High Blood Pressure: The JNC 7 Report,” The Journal of the American Medical Association 289 (2003): 2560–2571. 12748199 10.1001/jama.289.19.2560

[4] C. W. Ives and S. Oparil , “What Is the First Choice for Blood Pressure Treatment?,” The Lancet 394 (2019): 1782–1784. 10.1016/S0140-6736(19)32461-4 31668725

[5] S. P. Shah , G. P. Borah , K. Bhatti , R. Das , and D. K. Mehta , “Swotting Effective Hypertension Therapy: Ramipril Versus Telmisartan,” Journal of Medical Pharmaceutical and Allied Sciences 12 (2023): 5532–5539.

[6] D. K. Smith , R. P. Lennon , and P. B. Carlsgaard , “Managing Hypertension Using Combination Therapy,” American Family Physician 101 (2020): 341–349. 32163253

[7] C. P. Alderman , “Adverse Effects of the Angiotensin‐Converting Enzyme Inhibitors,” Annals of Pharmacotherapy 30 (1996): 55–61. 8773167 10.1177/106002809603000110

[8] L. M. Ruilope , E. A. Rosei , G. L. Bakris , et al., “Angiotensin Receptor Blockers: Therapeutic Targets and Cardiovascular Protection,” Blood Pressure 14 (2005): 196–209. 16126553 10.1080/08037050500230227

[9] 2023 ESH Hypertension Guideline Update: Bringing Us Closer Together Across the Pond ‐ American College of Cardiology, n.d., accessed January 23, 2025, https://www.acc.org/Latest‐in‐Cardiology/Articles/2024/02/05/11/43/2023‐ESH‐Hypertension‐Guideline‐Update.

[10] K. L. Schaefer and J. A. Porter , “Angiotensin II Receptor Antagonists: The Prototype Losartan,” Annals of Pharmacotherapy 30 (1996): 625–636. 8792950 10.1177/106002809603000611

[11] C. Dasgupta and L. Zhang , “Angiotensin II Receptors and Drug Discovery in Cardiovascular Disease,” Drug Discovery Today 16 (2010): 22. 21147255 10.1016/j.drudis.2010.11.016 PMC3022115

[12] J. Gao , J. Chao , K. J. K. Parbhu , et al., “Ontogeny of Angiotensin Type 2 and Type 1 Receptor Expression in Mice,” Journal of the Renin‐Angiotensin‐Aldosterone System 13 (2012): 341. 22526820 10.1177/1470320312443720 PMC3463952

[13] H. Tang , K. Li , Z. Shi , and J. Wu , “G‐Protein‐Coupled Receptors in Chronic Kidney Disease Induced by Hypertension and Diabetes,” Cells 14 (2025): 729, 10.3390/cells14100729. 40422232 PMC12109938

[14] J. Valgeirsson , E. Nielsen , D. Peters , C. Mathiesen , A. S. Kristensen , and U. Madsen , “Bioisosteric Modifications of 2‐Arylureidobenzoic Acids: Selective Noncompetitive Antagonists for the Homomeric Kainate Receptor Subtype GluR5,” Journal of Medicinal Chemistry 47 (2004): 6948–6957. 15615543 10.1021/jm030638w

[15] K. Indrasena Reddy , C. Aruna , M. Manisha , et al., “Synthesis, DNA Binding and In‐Vitro Cytotoxicity Studies on Novel Bis‐Pyrazoles,” Journal of Photochemistry and Photobiology B 168 (2017): 89–97. 10.1016/j.jphotobiol.2017.02.003 28189845

[16] N. Meddad‐Belhabich , D. Aoun , A. Djimdé , et al., “Design of New Potent and Selective Secretory Phospholipase A2 Inhibitors. 6‐Synthesis, Structure‐Activity Relationships and Molecular Modelling of 1‐Substituted‐4‐[4,5‐Dihydro‐1,2,4‐(4H)‐Oxadiazol‐5‐One‐3‐Yl(methyl)]‐Functionalized Aryl Piperazin/One/Dione Derivatives,” Bioorganic Medicinal Chemistry 18 (2010): 3588–3600. 20417107 10.1016/j.bmc.2010.03.049

[17] N. S. Soldatova , A. V. Semenov , K. K. Geyl , et al., “Copper‐Catalyzed Selective N‐Arylation of Oxadiazolones by Diaryliodonium Salts,” Advanced Synthesis and Catalysis 363 (2021): 3566–3576.

[18] L. Belvisi , “A 3D QSAR CoMFA Study of Non‐Peptide Angiotensin II Receptor Antagonists,” Journal of Computer‐Aided Molecular Design 10 (1996): 567–582. 9007690 10.1007/BF00134180

[19] J. V. Dos Anjos , D. Sinou , S. J. De Melo , and R. M. Srivastava , “Synthesis of Triazole‐Linked Manno‐ and Glucopyranosyl Amino Acids,” Synthesis 17 (2007): 2647–2652.

[20] P. Lassalas , B. Gay , C. Lasfargeas , et al., “Structure Property Relationships of Carboxylic Acid Isosteres,” Journal of Medicinal Chemistry 59 (2016): 3183–3203. 26967507 10.1021/acs.jmedchem.5b01963 PMC4833640

[21] N. Fukazawa , T. Nishimura , K. Orii , S. Noguchi , and M. Tomi , “Conversion of Olmesartan to Olmesartan Medoxomil, a Prodrug that Improves Intestinal Absorption, Confers Substrate Recognition by OATP2B1,” Pharmaceutical Research 41 (2024): 849–861. 38485855 10.1007/s11095-024-03687-1

[22] V. V. Rostovtsev , L. G. Green , V. V. Fokin , and K. B. Sharpless , “A Stepwise Huisgen Cycloaddition Process: Copper(I)‐Catalyzed Regioselective Ligation of Azides and Terminal Alkynes,” Angewandte Chemie International Edition 41 (2002): 2596–2599. 12203546 10.1002/1521-3773(20020715)41:14<2596::AID-ANIE2596>3.0.CO;2-4

[23] J. V. dos Anjos , D. Sinou , S. J. de Melo , and R. M. Srivastava , “Synthesis of Glycosyl‐Triazole Linked 1,2,4‐Oxadiazoles,” Carbohydrate Research 342 (2007): 2440–2449. 17689508 10.1016/j.carres.2007.07.011

[24] A. Barreras and C. Gurk‐Turner , Angiotensin II Receptor Blockers (Baylor University Medical Center Proceedings, 2003). 123–126. 10.1080/08998280.2003.11927893 PMC1200815 16278727

[25] E. Kaschina and T. Unger , “Angiotensin AT1/AT2 Receptors: Regulation, Signalling and Function,” Blood Pressure 12 (2003): 70–88. 12797627 10.1080/08037050310001057

[26] H. Zhang , H. Unal , C. Gati , et al., “Structure of the Angiotensin Receptor Revealed by Serial Femtosecond Crystallography,” Cell 161 (2015): 833–844. 25913193 10.1016/j.cell.2015.04.011 PMC4427029

[27] “SwissADME,” n.d., accessed February 11, 2026, http://www.swissadme.ch/index.php.

[28] A. Daina , O. Michielin , and V. Zoete , “SwissADME: A Free Web Tool to Evaluate Pharmacokinetics, Drug‐Likeness and Medicinal Chemistry Friendliness of Small Molecules,” Scientific Reports 7 (2017): 1–13. 28256516 10.1038/srep42717 PMC5335600

[29] M. Stielow , A. Witczyńska , N. Kubryń , Ł. Fijałkowski , J. Nowaczyk , and A. Nowaczyk , “The Bioavailability of Drugs—the Current State of Knowledge,” Molecules 28 (2023): 8038, 10.3390/MOLECULES28248038. 38138529 PMC10745386

[30] Y. C. Martin , “A Bioavailability Score,” Journal of Medicinal Chemistry 48 (2005): 3164–3170. 15857122 10.1021/jm0492002

[31] D. Wu , Q. Chen , X. Chen , F. Han , Z. Chen , and Y. Wang , “The Blood–brain Barrier: Structure, Regulation, and Drug Delivery,” Signal Transduction and Targeted Therapy 8 (2023): 1–27. 37231000 10.1038/s41392-023-01481-w PMC10212980

[32] W. Zhu , Y. Wang , Y. Niu , L. Zhang , and Z. Liu , “Current Trends and Challenges in Drug‐Likeness Prediction: Are They Generalizable and Interpretable?,” Health Data Science 3 (2023): 98, 10.34133/HDS.0098. PMC10880170 38487200

[33] J. B. Baell and J. W. M. Nissink , “7 Year Itch: Pan‐Assay Interference Compounds (PAINS) in 2017—Utility and Limitations,” ACS Chemical Biology 13 (2018): 36. 29202222 10.1021/acschembio.7b00903 PMC5778390

[34] W. L. Armarego , and C. Chai , Purification of Laboratory Chemicals (Butterworth‐Heinemann, Elsevier, 2009). 6th.

[35] R. M. Srivastava , M. C. Pereira , W. W. M. Faustino , K. Coutinho , J. V. dos Anjos , and S. J. De Melo , “Synthesis, Mechanism of Formation, and Molecular Orbital Calculations of Arylamidoximes,” Monatshefte für Chemie ‐ Chemical Monthly 140 (2009): 1319–1324.

[36] L. F. L. Ferreira , I. M. S. Lins , S. G. D. Feitosa , et al., “New Oxadiazol‐5‐Ones Derivatives and Their Performance as Angiotensin‐Converting Enzyme (ACE) Inhibitors,” Journal of Heterocyclic Chemistry 61 (2024): 1125–1140.

[37] G. Jones , P. Willett , and R. C. Glen , “Molecular Recognition of Receptor Sites Using a Genetic Algorithm With a Description of Desolvation,” Journal of Molecular Biology 245 (1995): 43–53. 7823319 10.1016/s0022-2836(95)80037-9

[38] M. D. Hanwell , D. E. Curtis , D. C. Lonie , T. Vandermeerschd , E. Zurek , and G. R. Hutchison , “Avogadro: An Advanced Semantic Chemical Editor, Visualization, and Analysis Platform,” Journal of Cheminformatics 4 (2012): 1–17. 22889332 10.1186/1758-2946-4-17 PMC3542060

[39] “PyMOL | pymol.org,” n.d., accessed February 12, 2026, https://www.pymol.org/.

[40] “BIOVIA Discovery Studio | Dassault Systèmes,” n.d., accessed February 12, 2026, https://www.3ds.com/products/biovia/discovery‐studio.

