# Supplementary material for: Oxadiazolone‐Triazole Derivatives as a New Scaffold for Modulation of Angiotensin II‐Induced Vascular Contraction
Source: ChemMedChem. 2026 Aug 31;21(17):e70460. doi: 10.1002/cmdc.70460 (PMC13530322; doi:10.1002/cmdc.70460)
Supplement: Supplementary file 1 [file CMDC-21-e70460-s001.pdf]

SUPPORTING INFORMATION

Oxadiazolone-triazole derivatives as a new scaffold for modulation of angiotensin II-induced vascular contraction

Larissa F. L. Ferreira,[a§] Daiana G. de A. Vasconcelos,[a§] Ruth C. A. Santos[b], Jivaldo G. Ferreira[b], Alice V. Araújo[b]* and Janaína V. dos Anjos[a*]

[a] Department of Fundamental Chemistry, Federal University of Pernambuco (UFPE), Recife, 50740-560, Brazil

[b] Vitória Academic Centre (CAV), Federal University of Pernambuco (UFPE), Vitória de Santo Antão, PE, 50608-680, Brazil.

*E-mail: alice.araujo@ufpe.br; janaina.anjos@ufpe.br

Hydrogen (^1H) and Carbon(^{13}C) Nuclear Magnetic Resonance (NMR) spectra for compounds 7a–j.

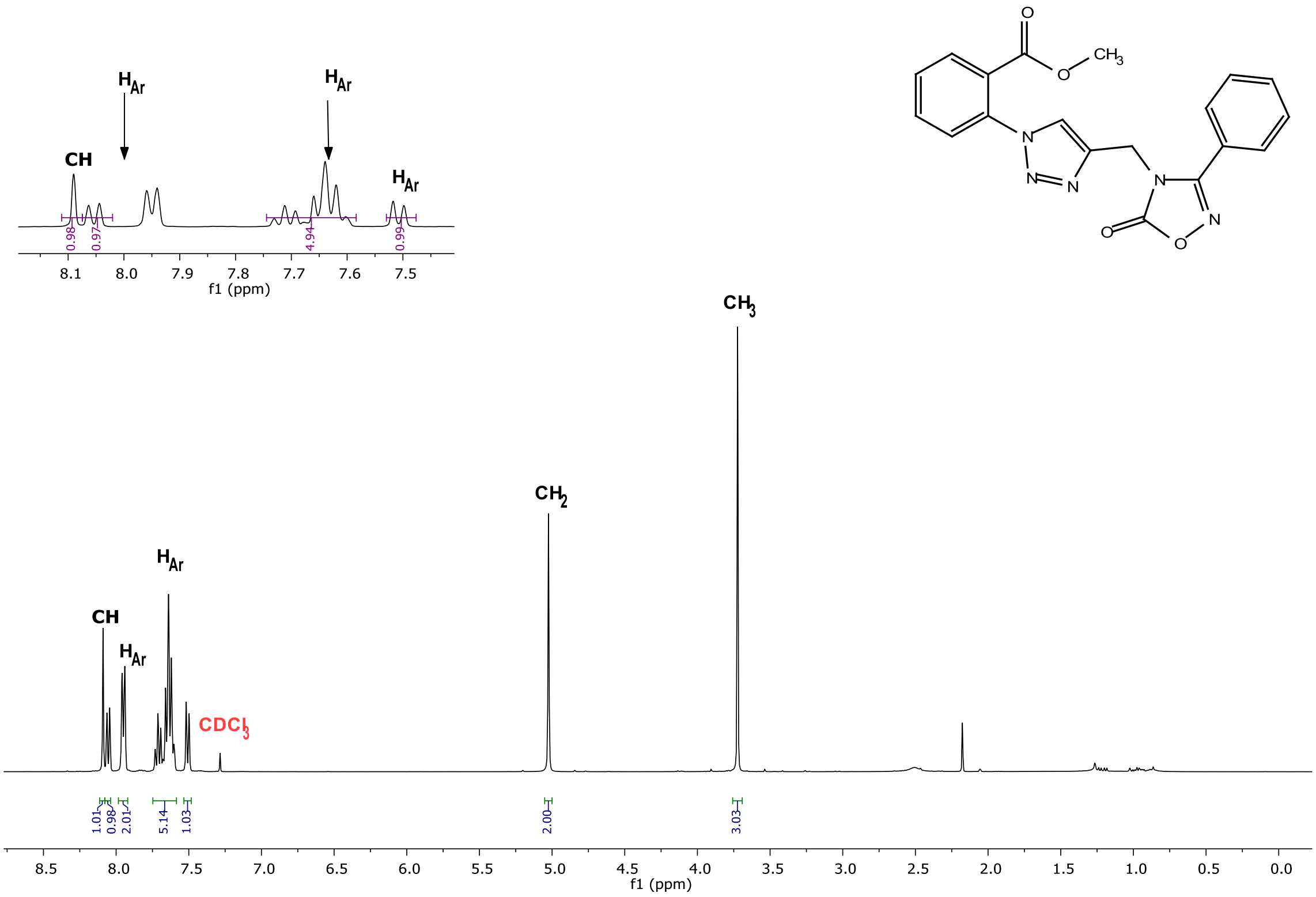

Figure S1. ^1H NMR spectrum ($CDCl_3$, 400 MHz) of compound **7a**.

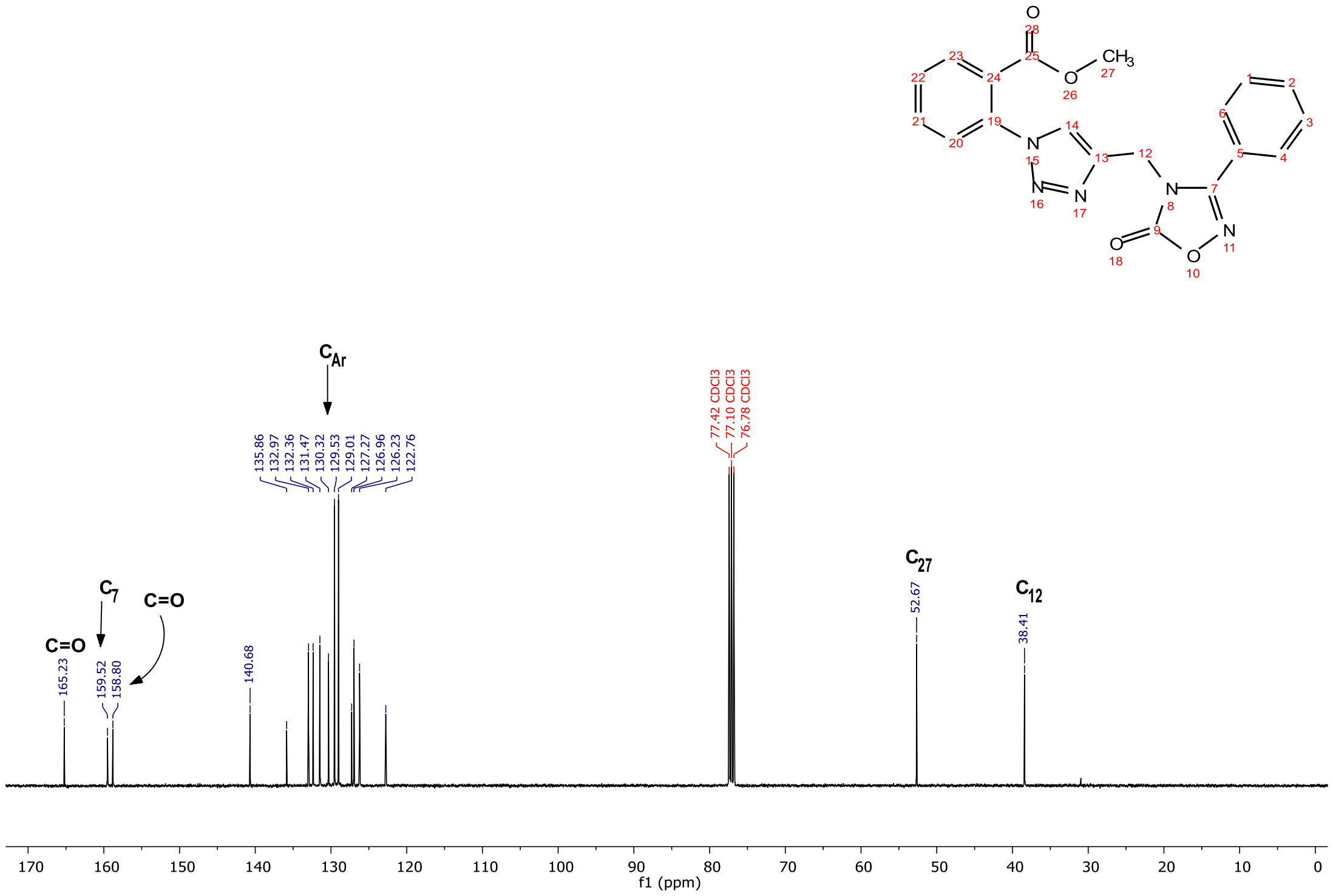

Figure S2. ^{13}C NMR spectrum ($CDCl_3$, 101 MHz) of compound **7a**.

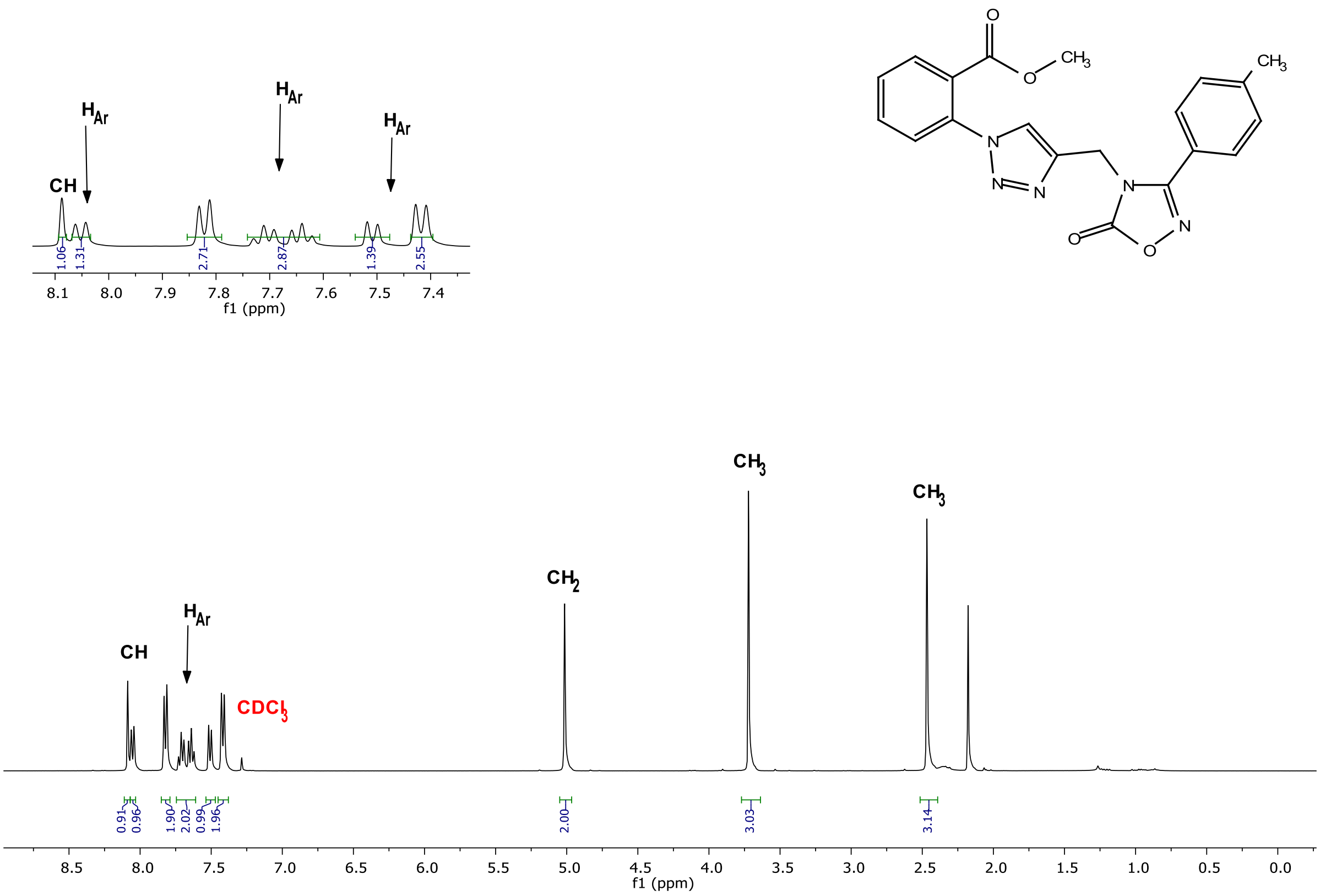

Figure S3. ^{1}H NMR spectrum ($CDCl_3$, 400 MHz) of compound **7b**.

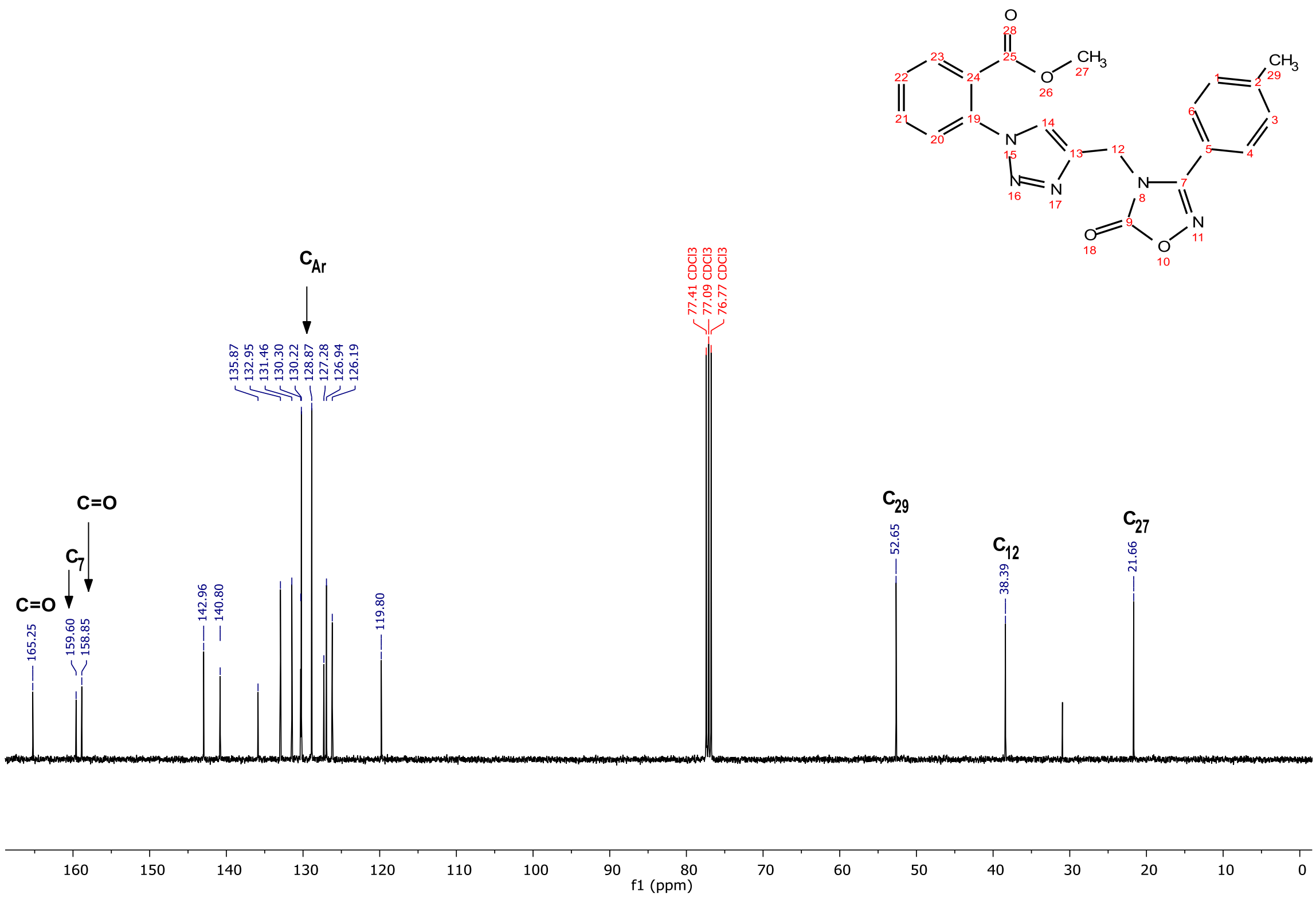

Figure S4. ^{13}C NMR spectrum ($CDCl_3$, 101 MHz) of compound **7b**.

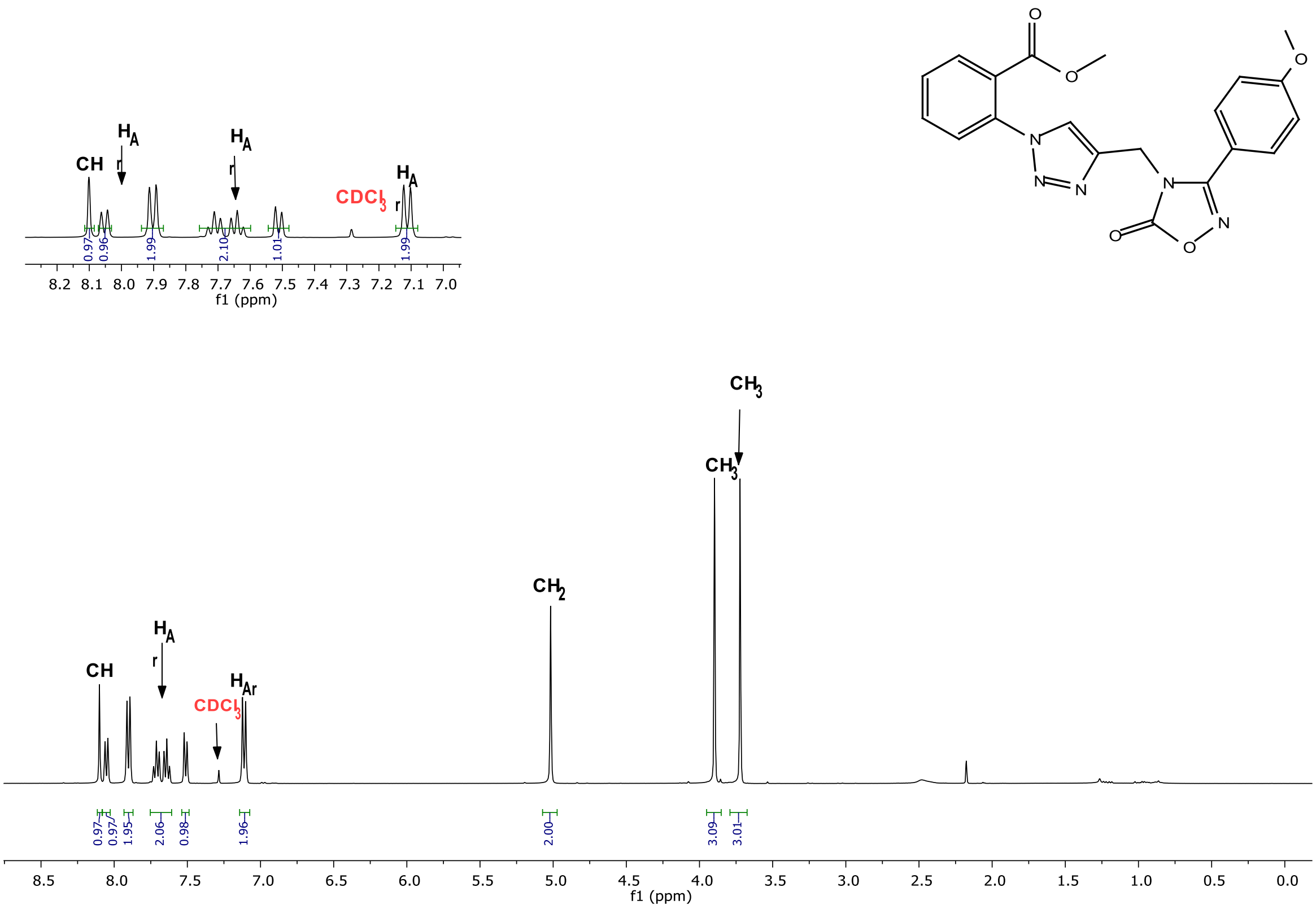

Figure S5. ^{1}H NMR spectrum ($CDCl_3$, 400 MHz) of compound **7c**.

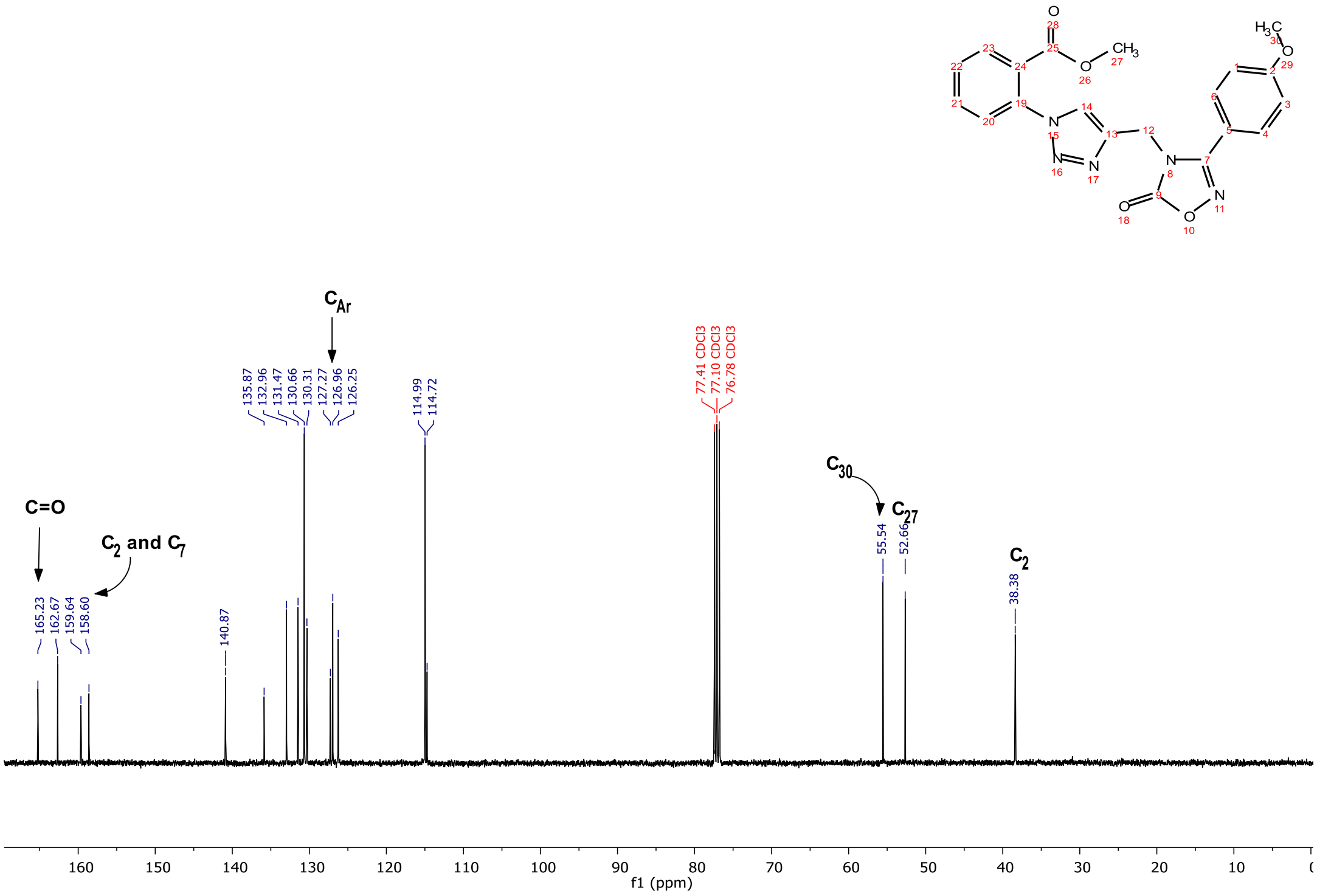

Figure S6. ^{13}C NMR spectrum ($CDCl_3$, 101 MHz) of compound **7c**.

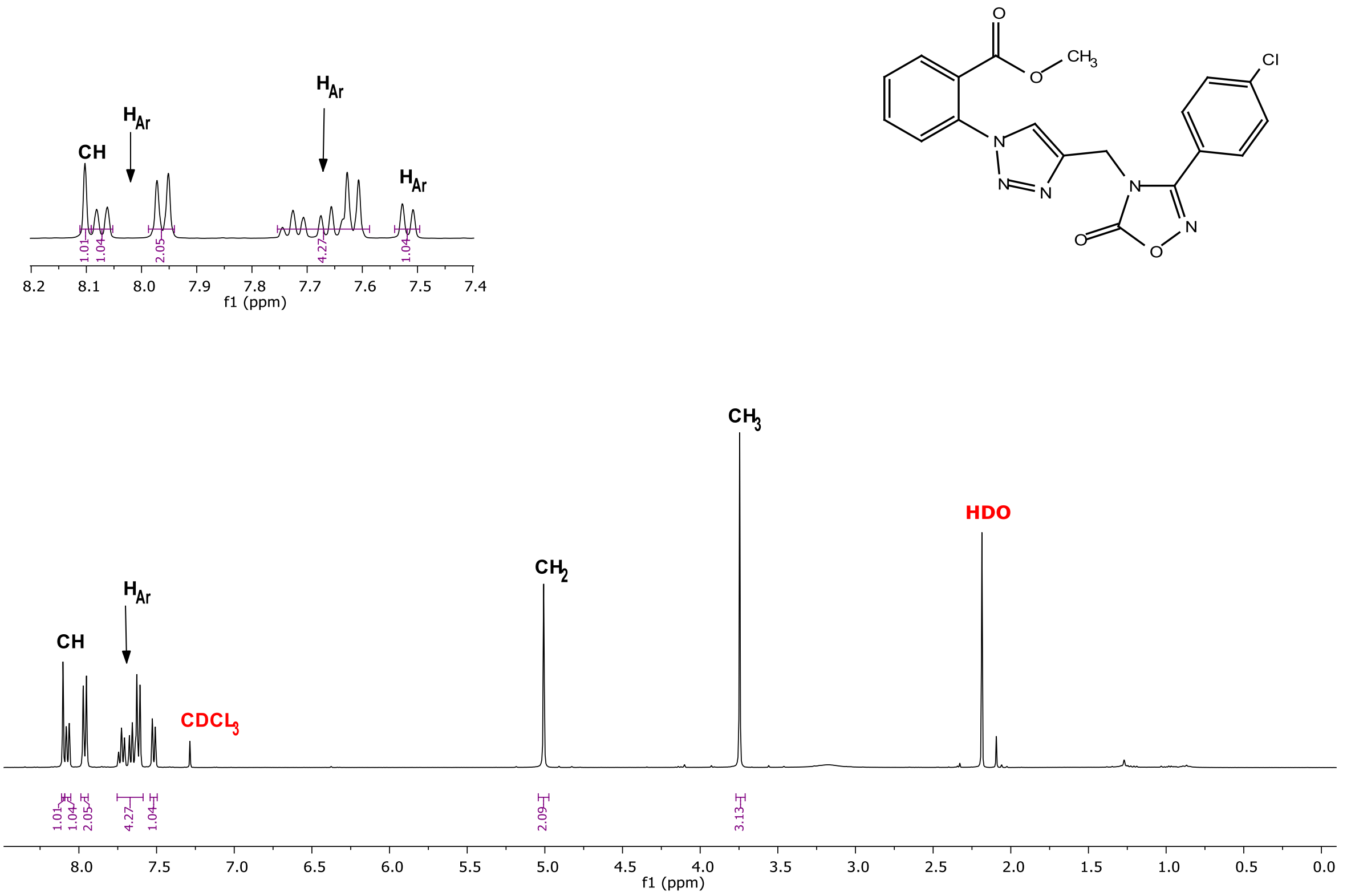

Figure S7. ^{1}H NMR spectrum ($CDCl_3$, 400 MHz) of compound **7d**.

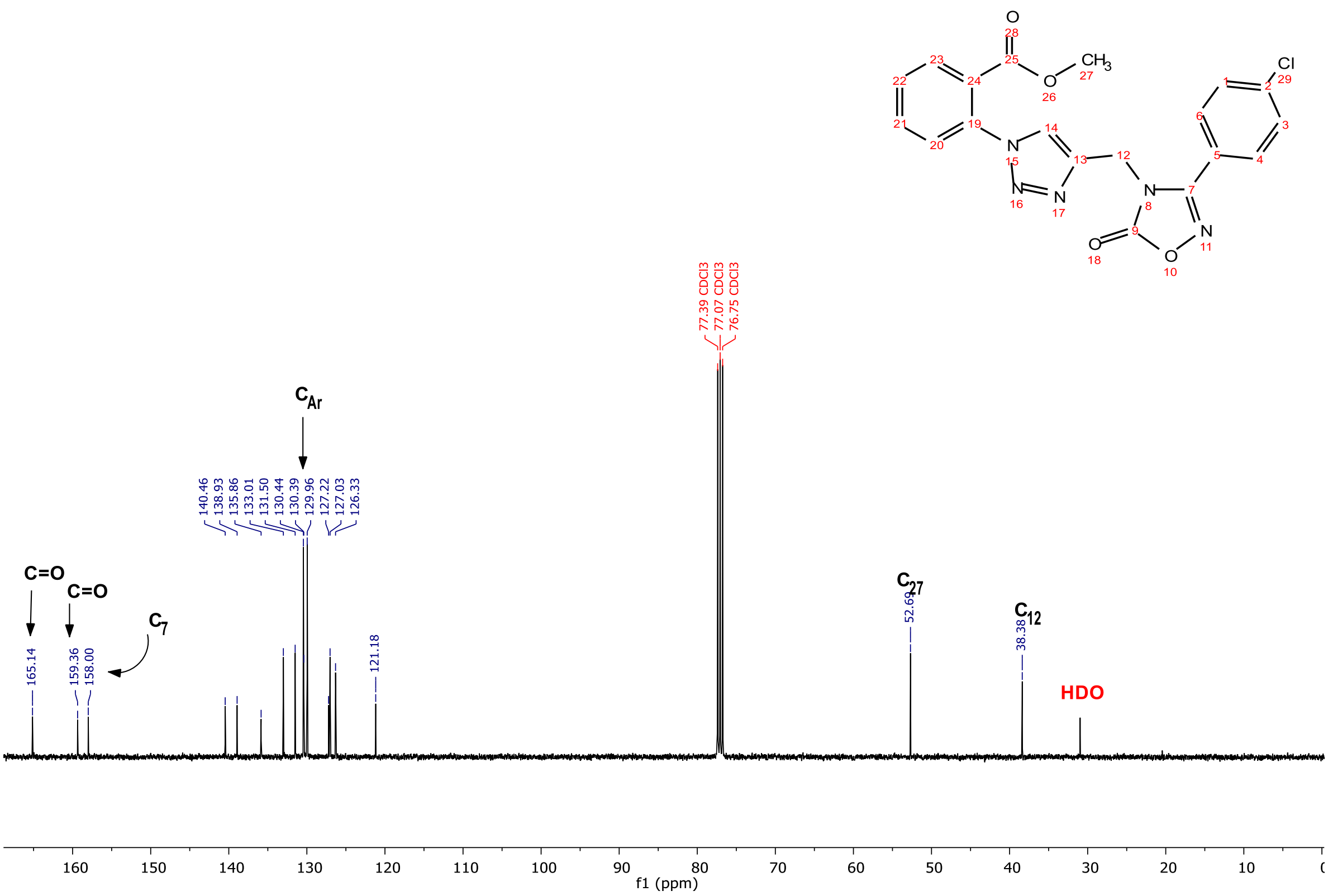

Figure S8. ^{13}C NMR spectrum ($CDCl_3$, 101 MHz) of compound **7d**.

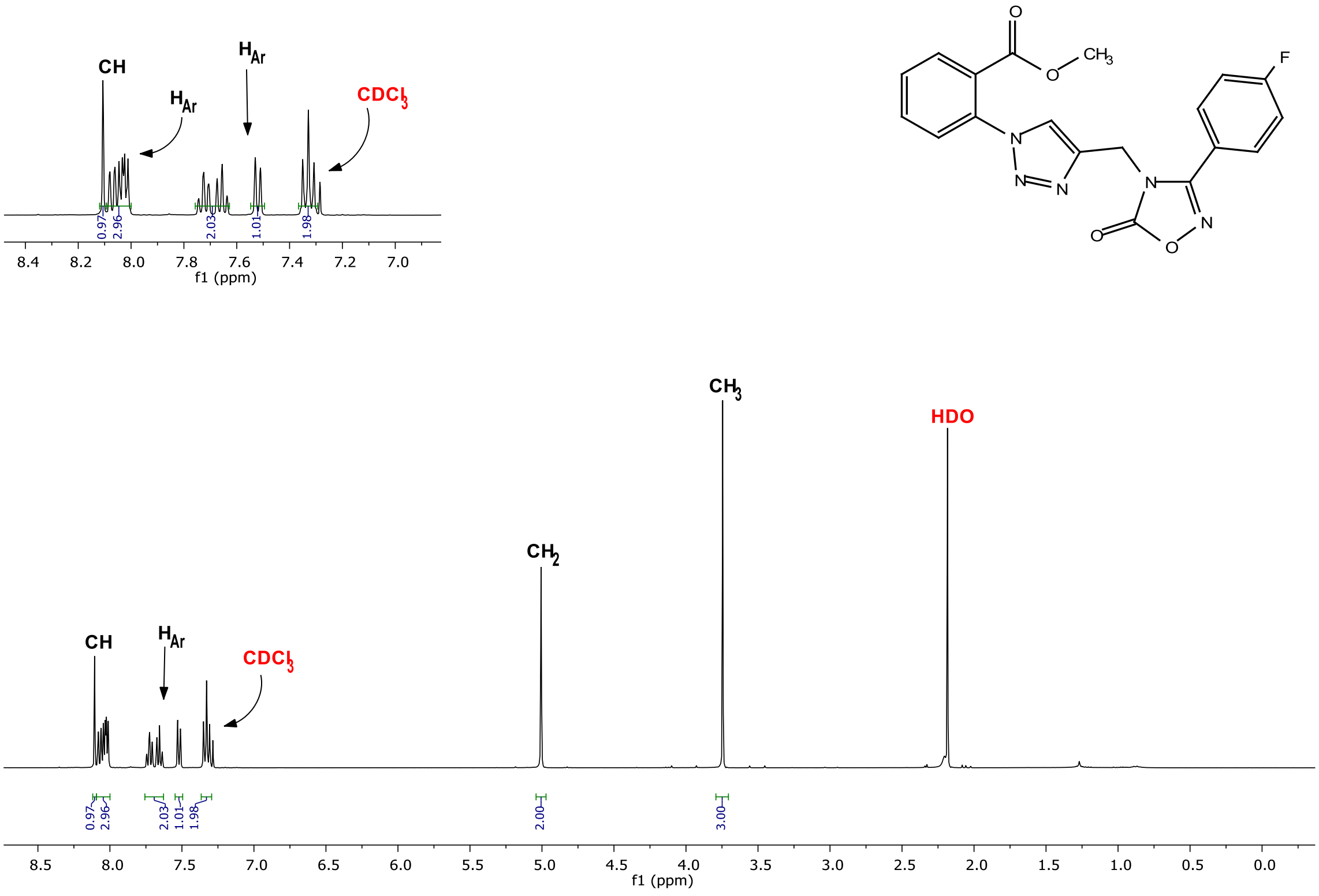

Figure S9. ^{1}H NMR spectrum ($CDCl_3$, 400 MHz) of compound **7e**.

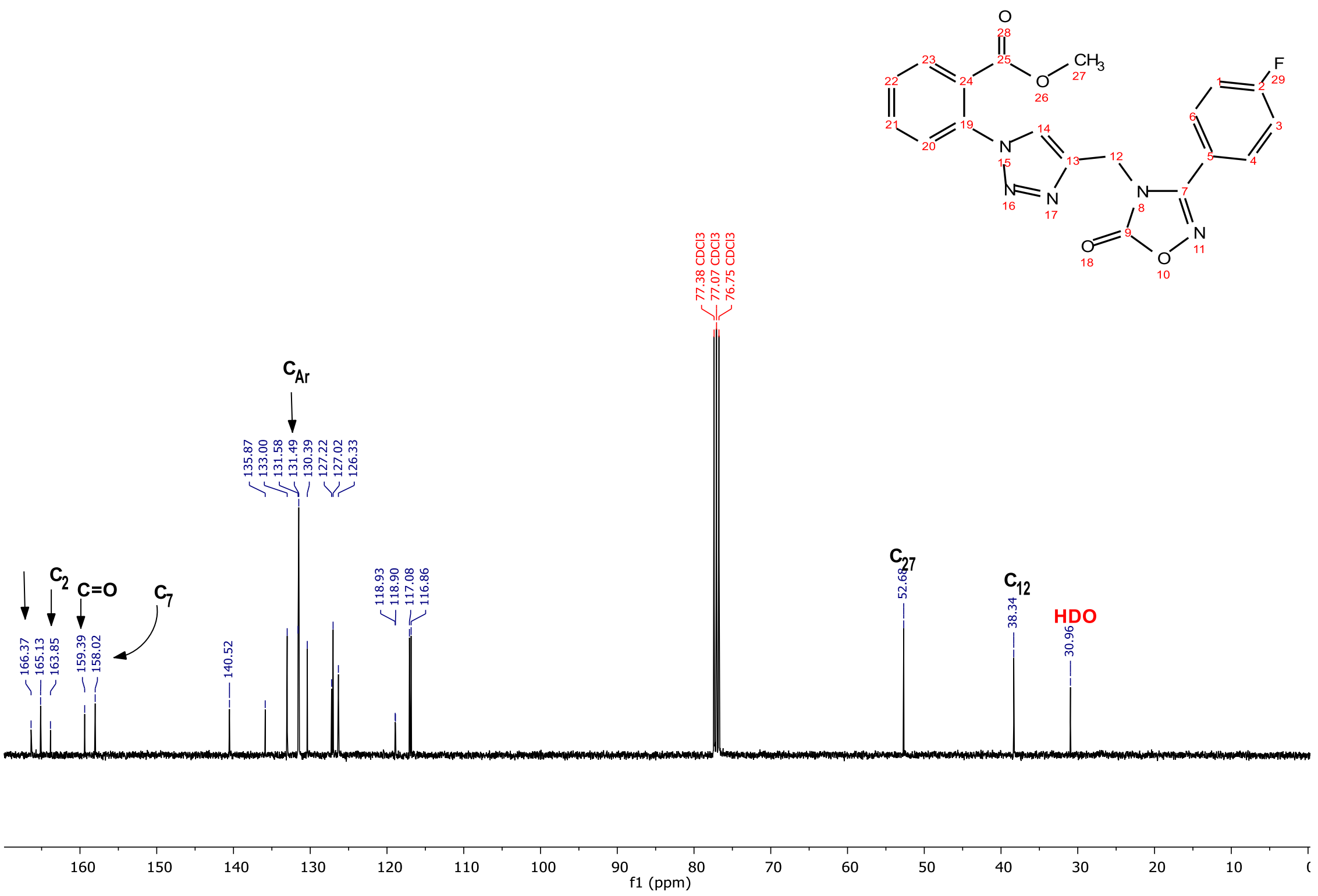

Figure S10. ^{13}C NMR spectrum ($CDCl_3$, 101 MHz) of compound **7e**.

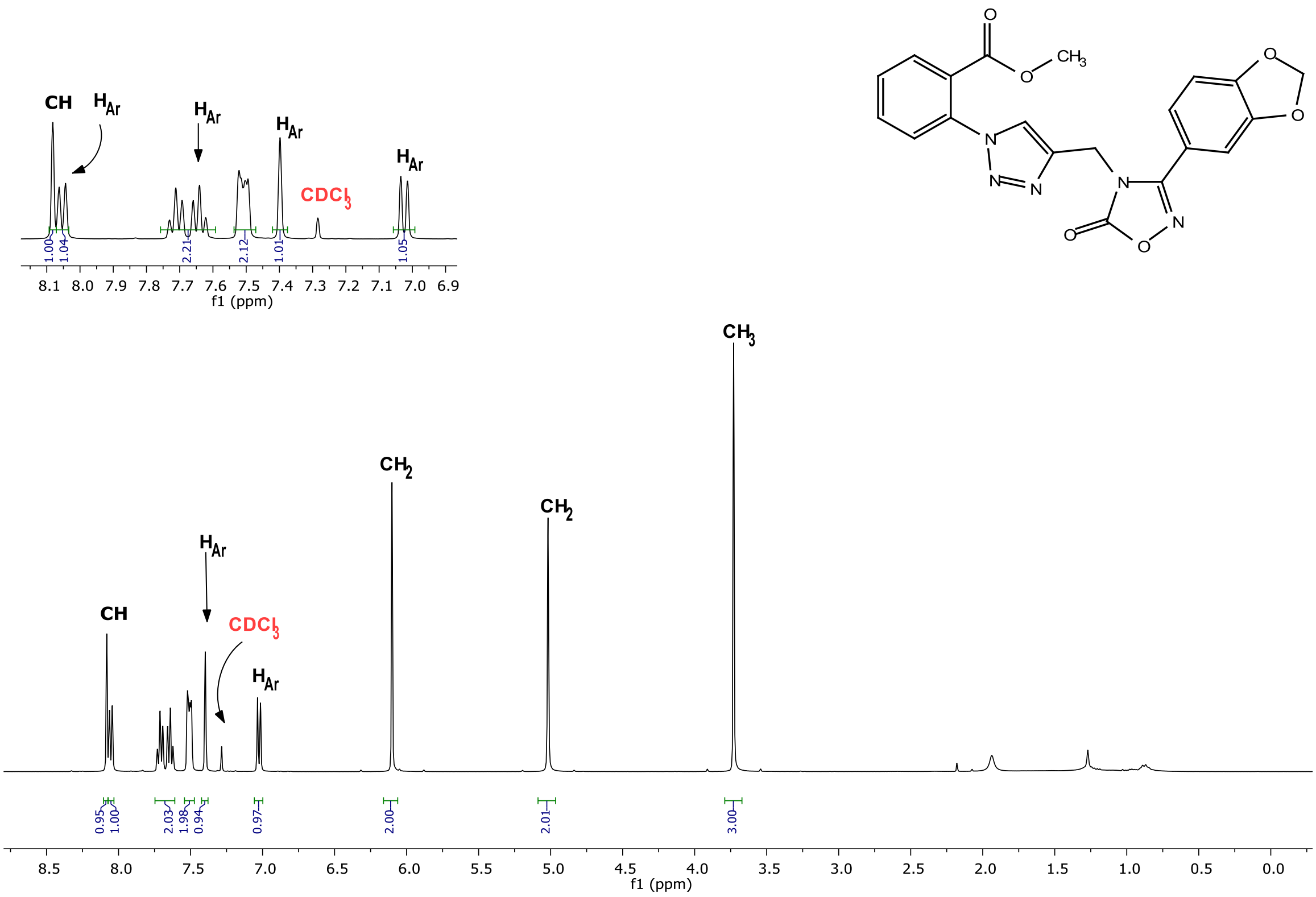

Figure S11. ^{1}H NMR spectrum ($CDCl_3$, 400 MHz) of compound **7f**.

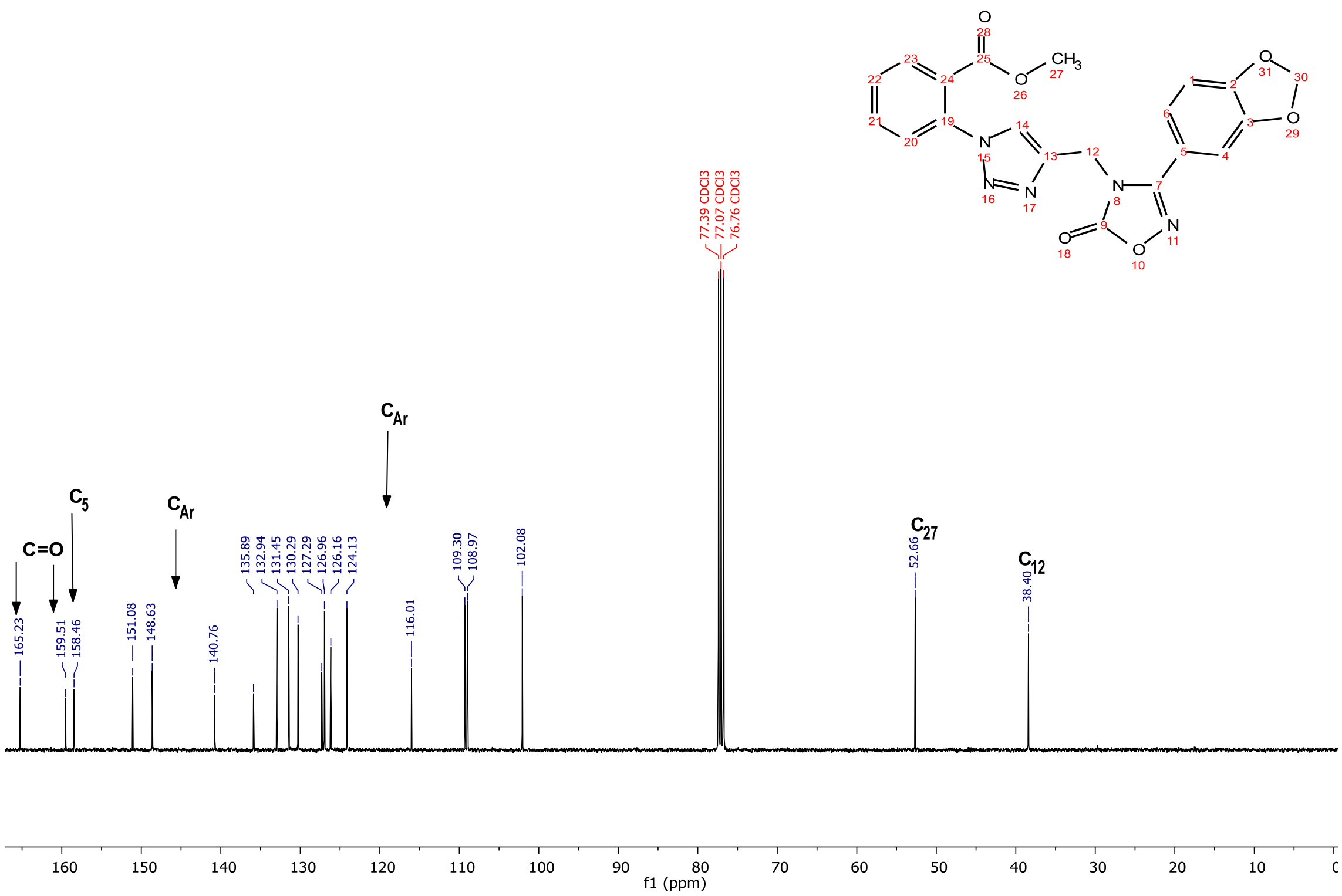

Figure S12. ^{13}C NMR spectrum ($CDCl_3$, 101 MHz) of compound **7f**.

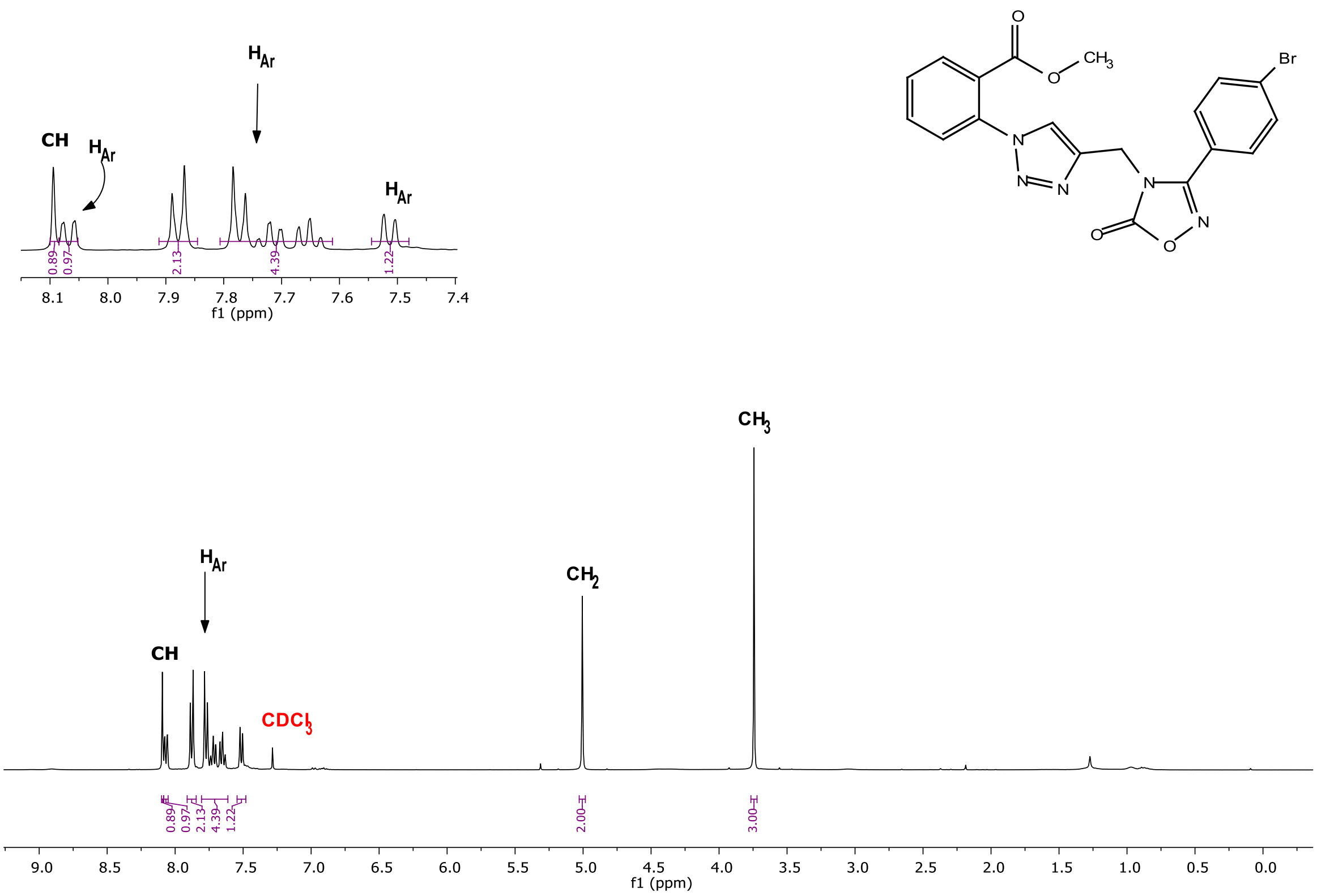

Figure S13. ^{1}H NMR spectrum ($CDCl_3$, 400 MHz) of compound **7g**.

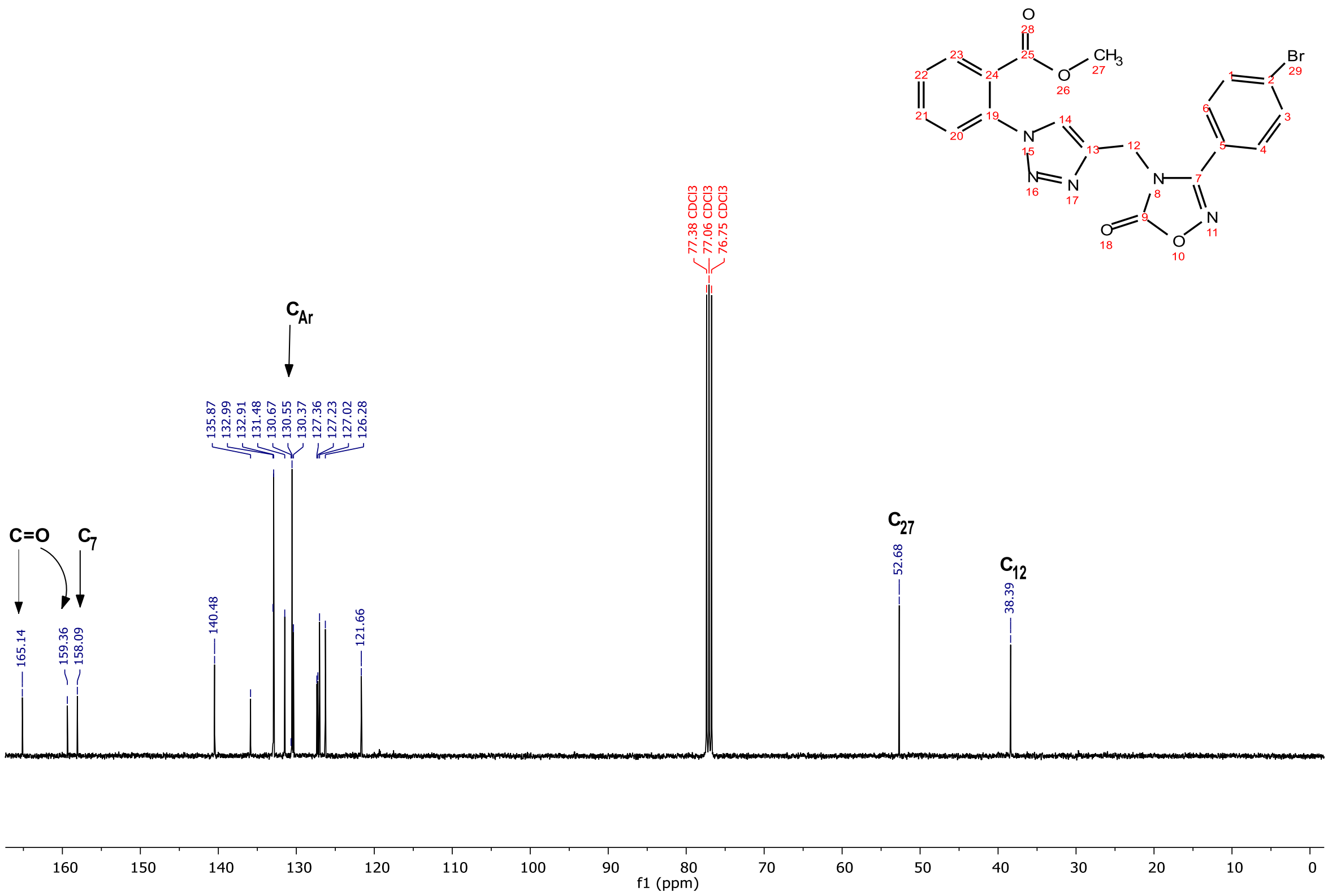

Figure S14. ^{13}C NMR spectrum ($CDCl_3$, 101 MHz) of compound **7g**.

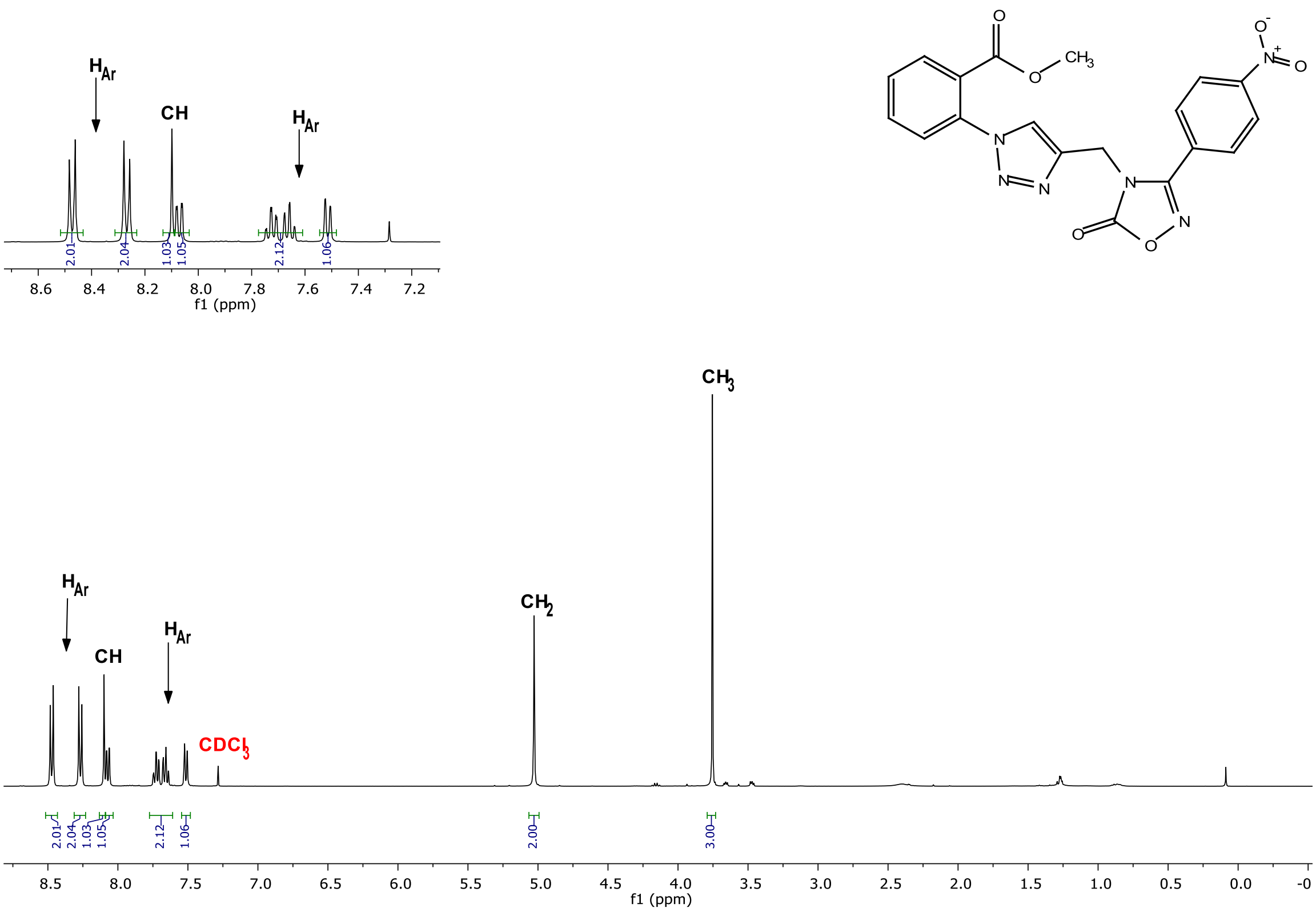

Figure S15. ^{1}H NMR spectrum ($CDCl_3$, 400 MHz) of compound **7h**.

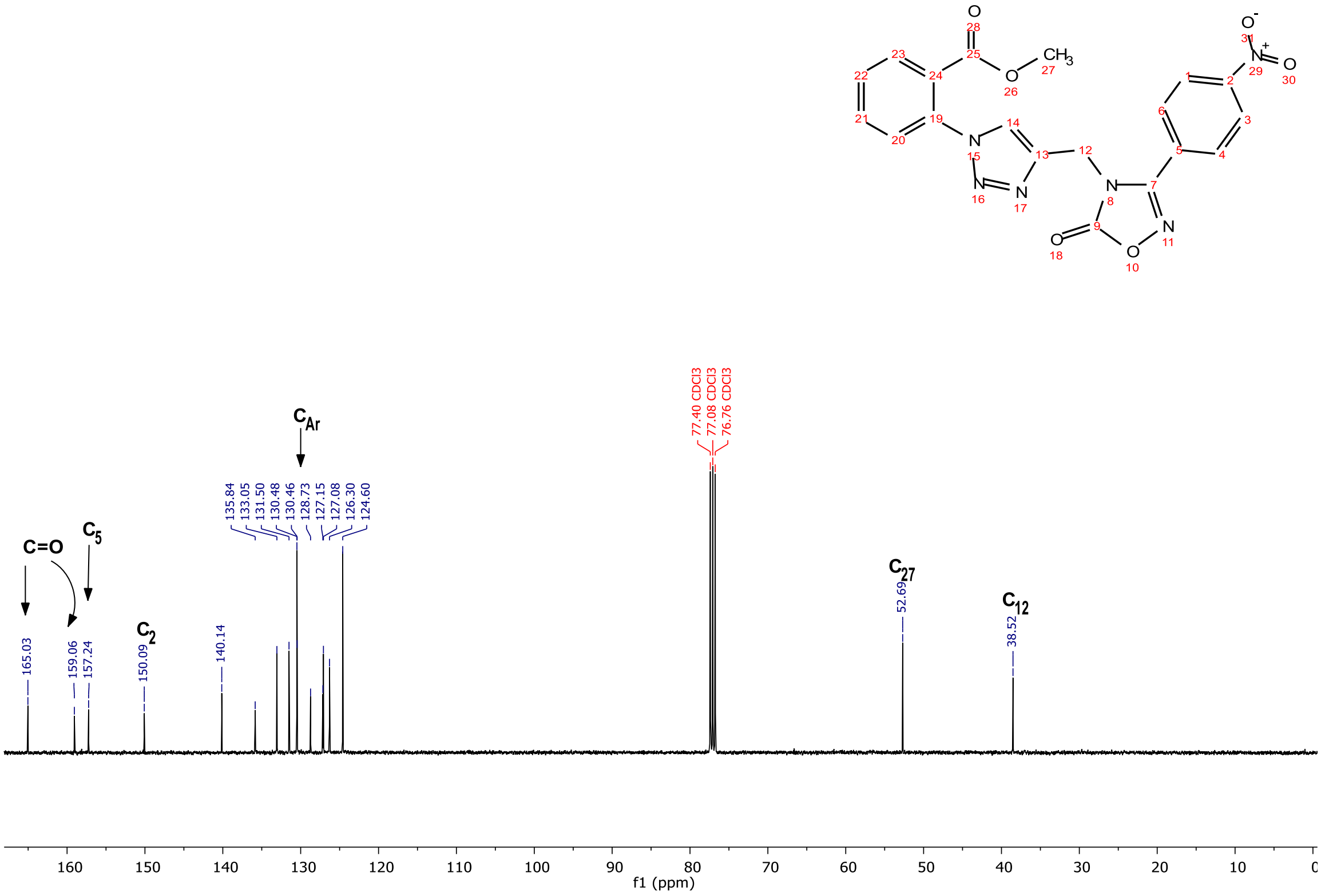

Figure S16. ^{13}C NMR spectrum ($CDCl_3$, 101 MHz) of compound **7h**.

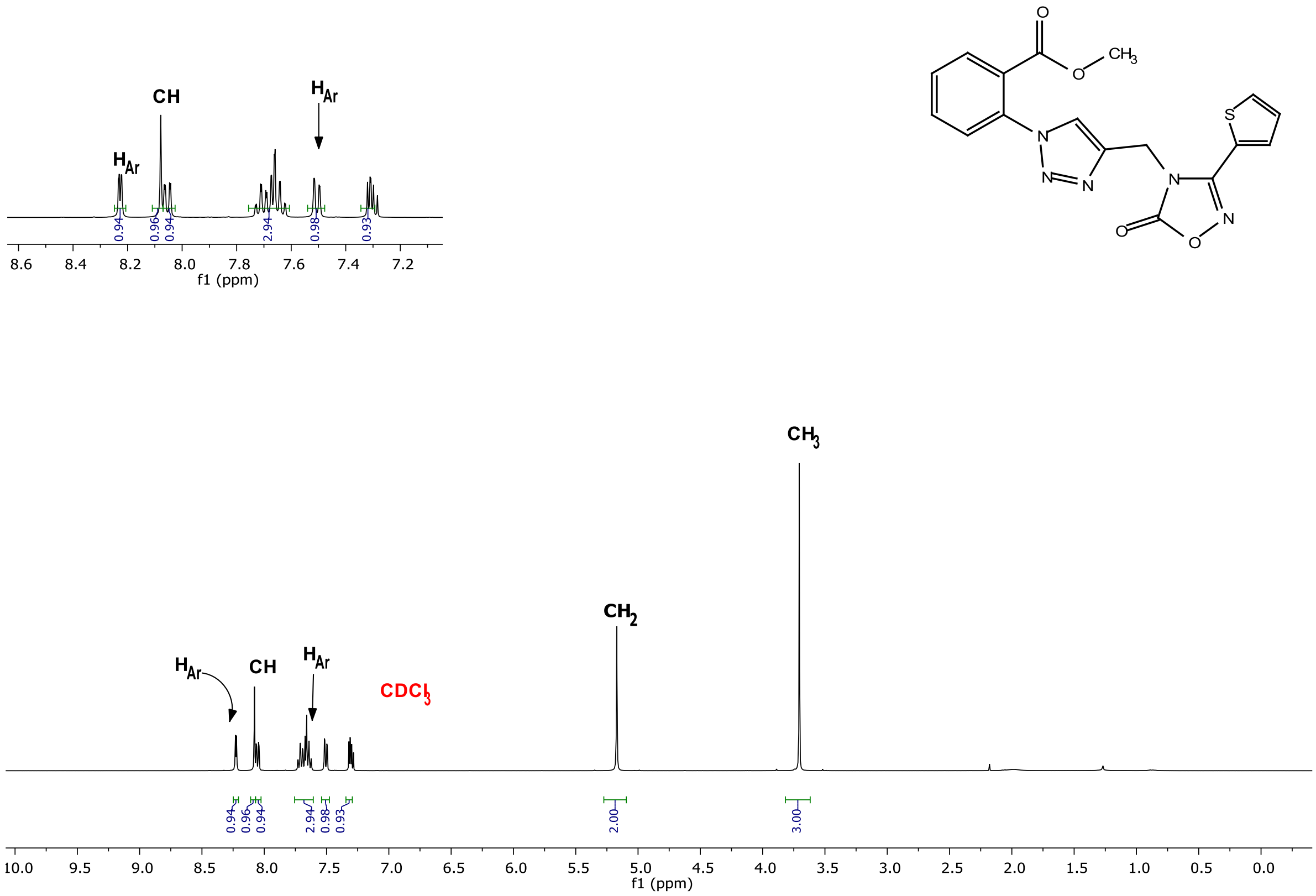

Figure S17. ^{1}H NMR spectrum ($CDCl_3$, 400 MHz) of compound **7i**.

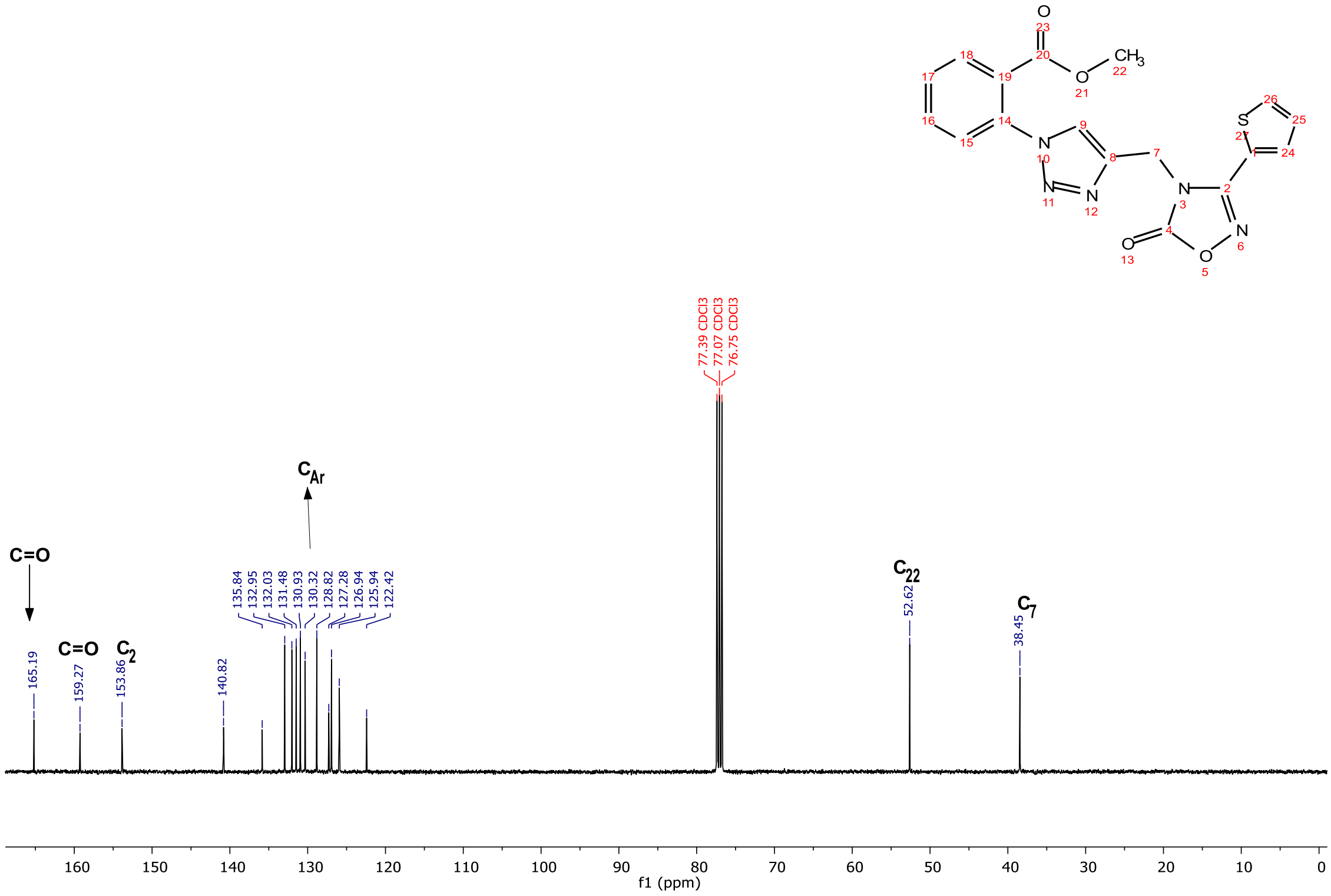

Figure S18. ^{13}C NMR spectrum ($CDCl_3$, 101 MHz) of compound **7i**.

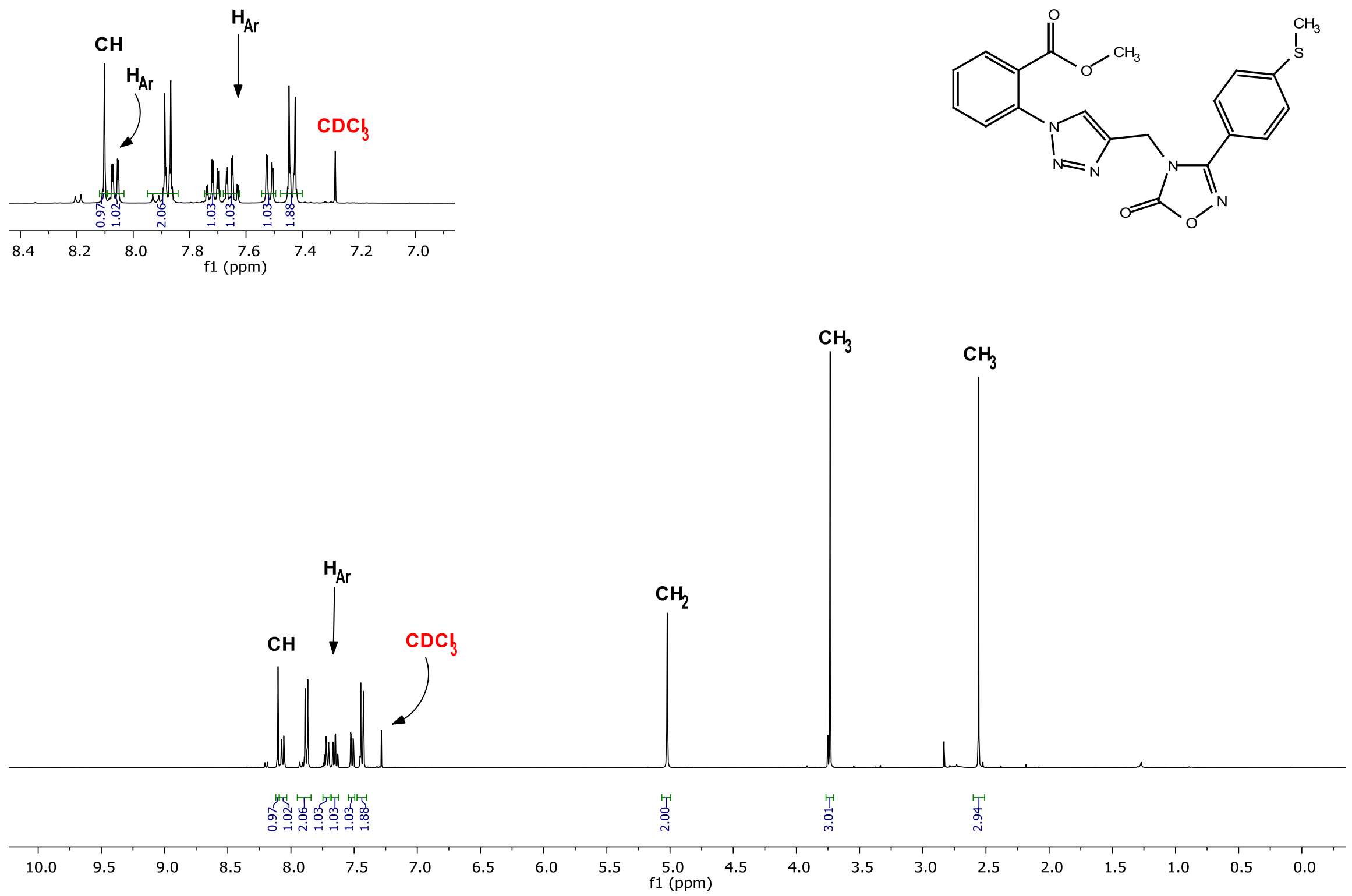

Figure S19. ^{1}H NMR spectrum ($CDCl_3$, 400 MHz) of compound **7j**.

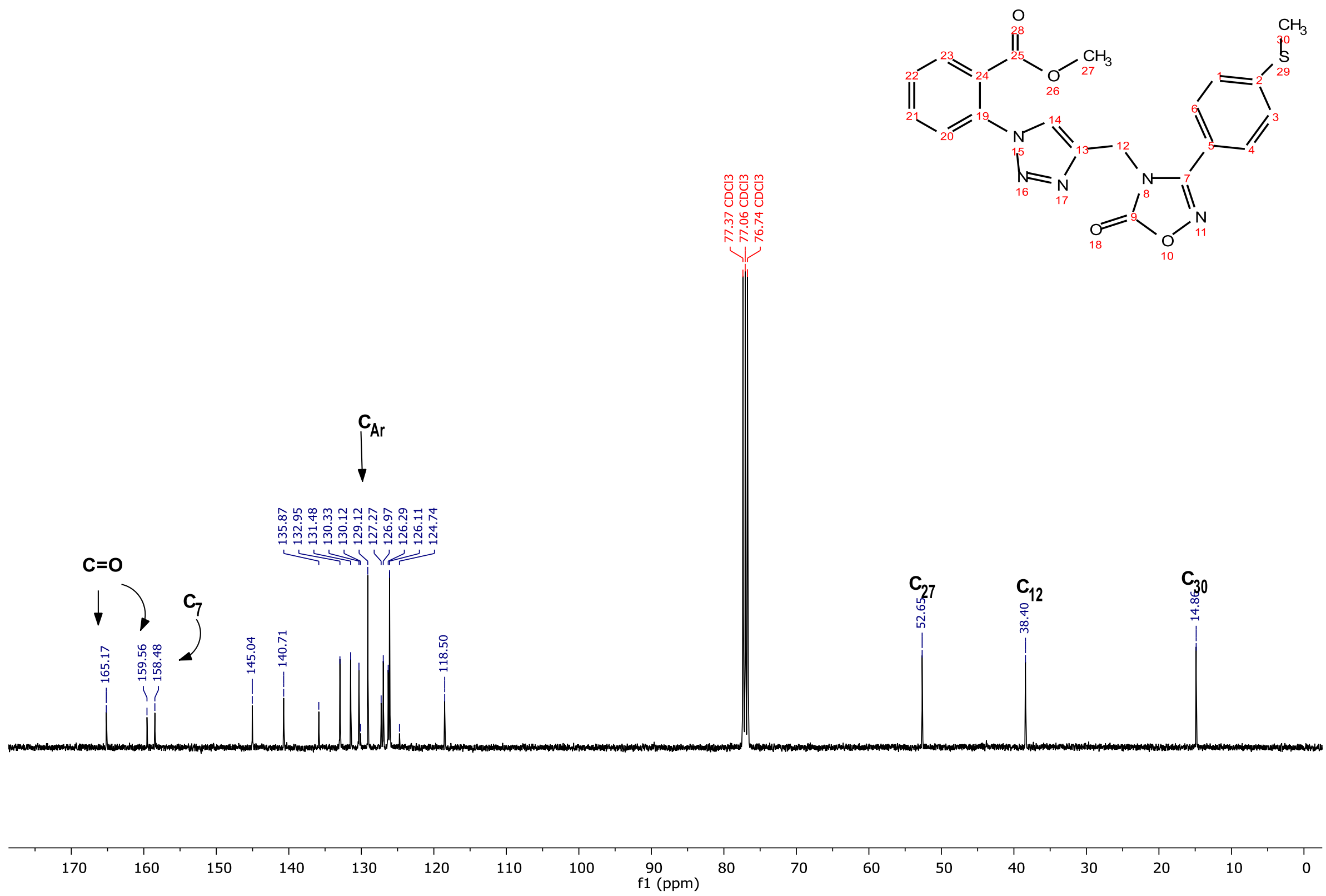

Figure S20. ^{13}C NMR spectrum ($CDCl_3$, 101 MHz) of compound **7j**.

High-resolution mass spectrometry (HRMS) data for compounds 7a–j

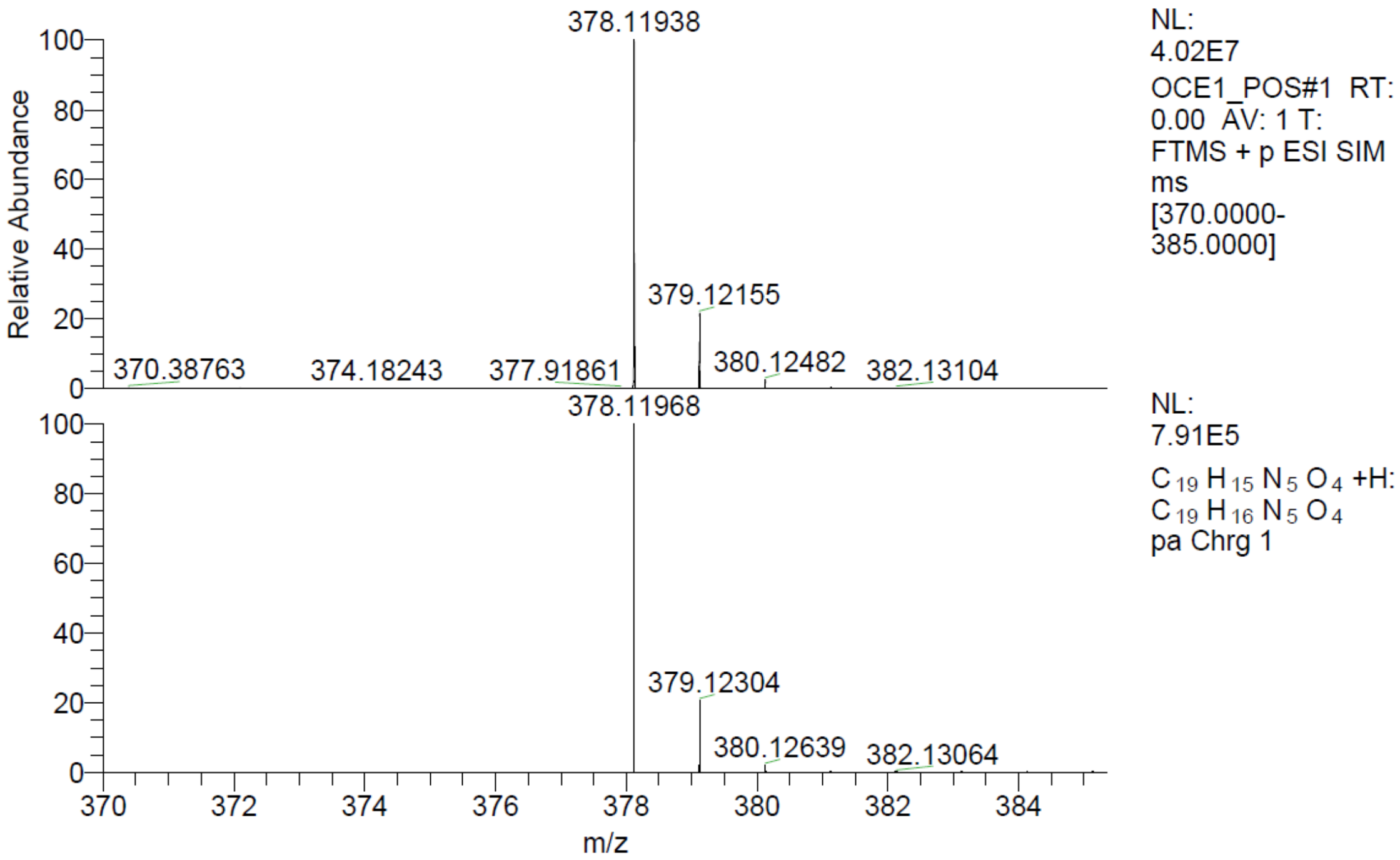

Figure S21. HRMS spectrum of compound **7a**.

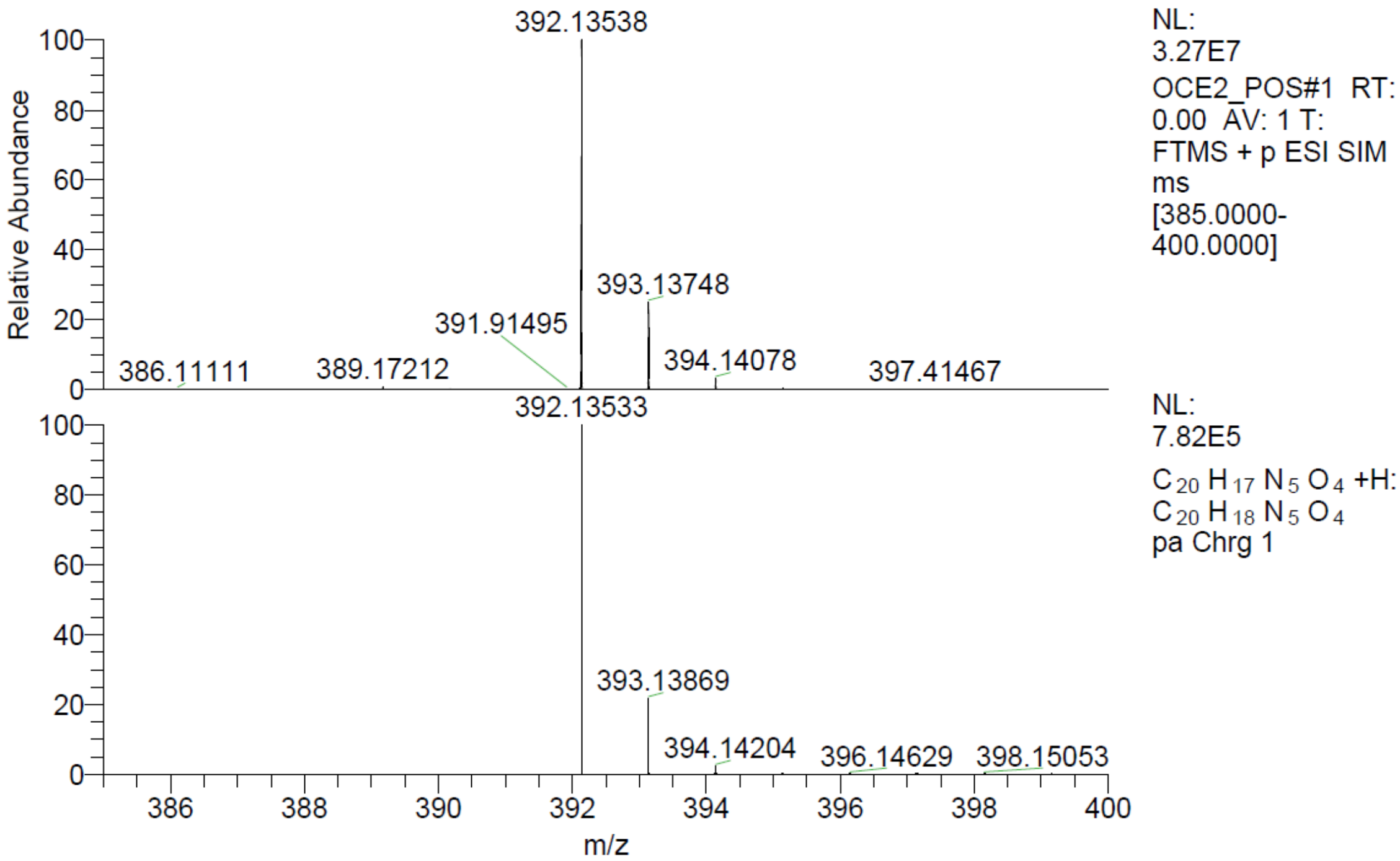

Figure S22. HRMS spectrum of compound **7b**.

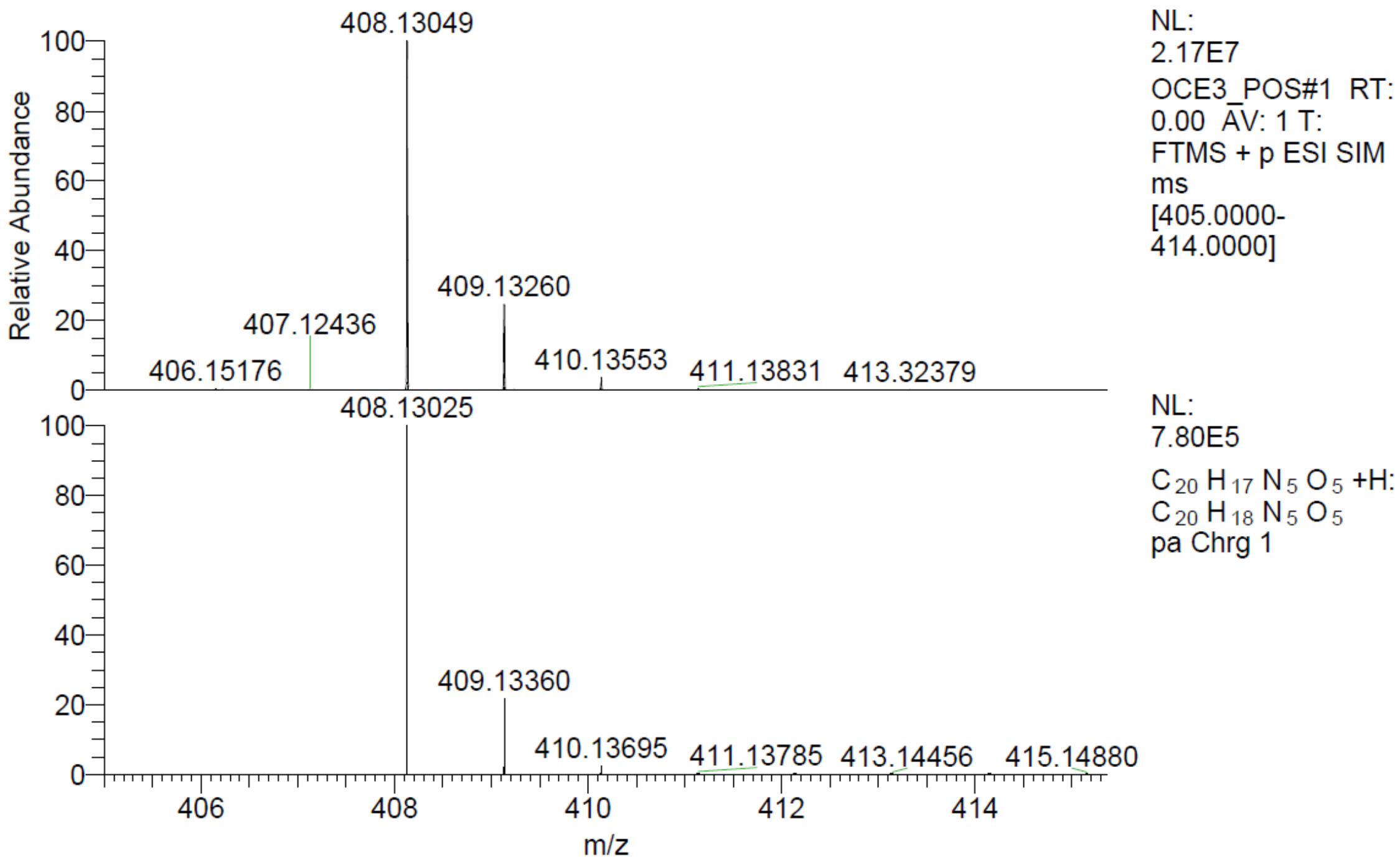

Figure S23. HRMS spectrum of compound **7c**.

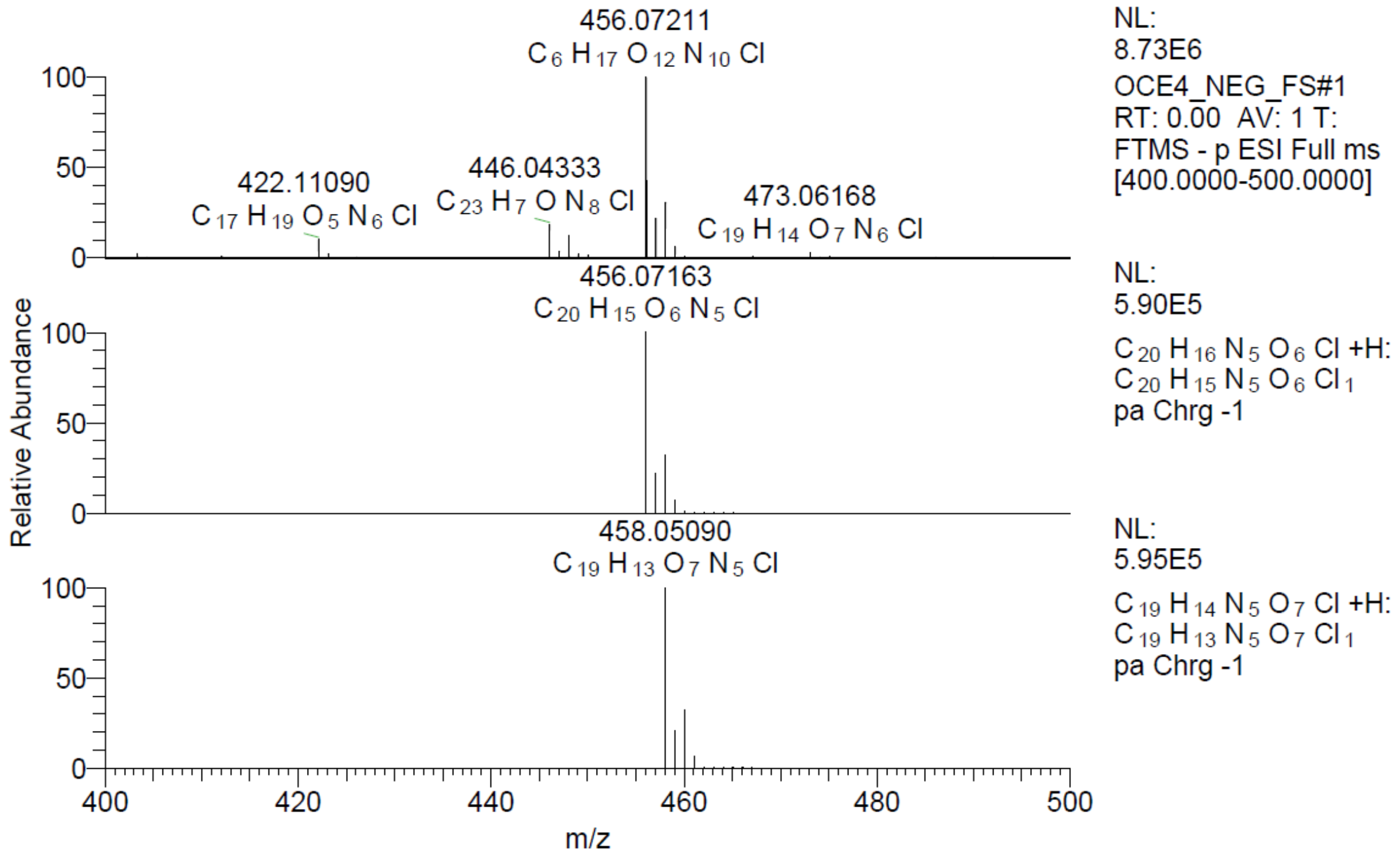

Figure S24. HRMS spectrum of compound **7d**.

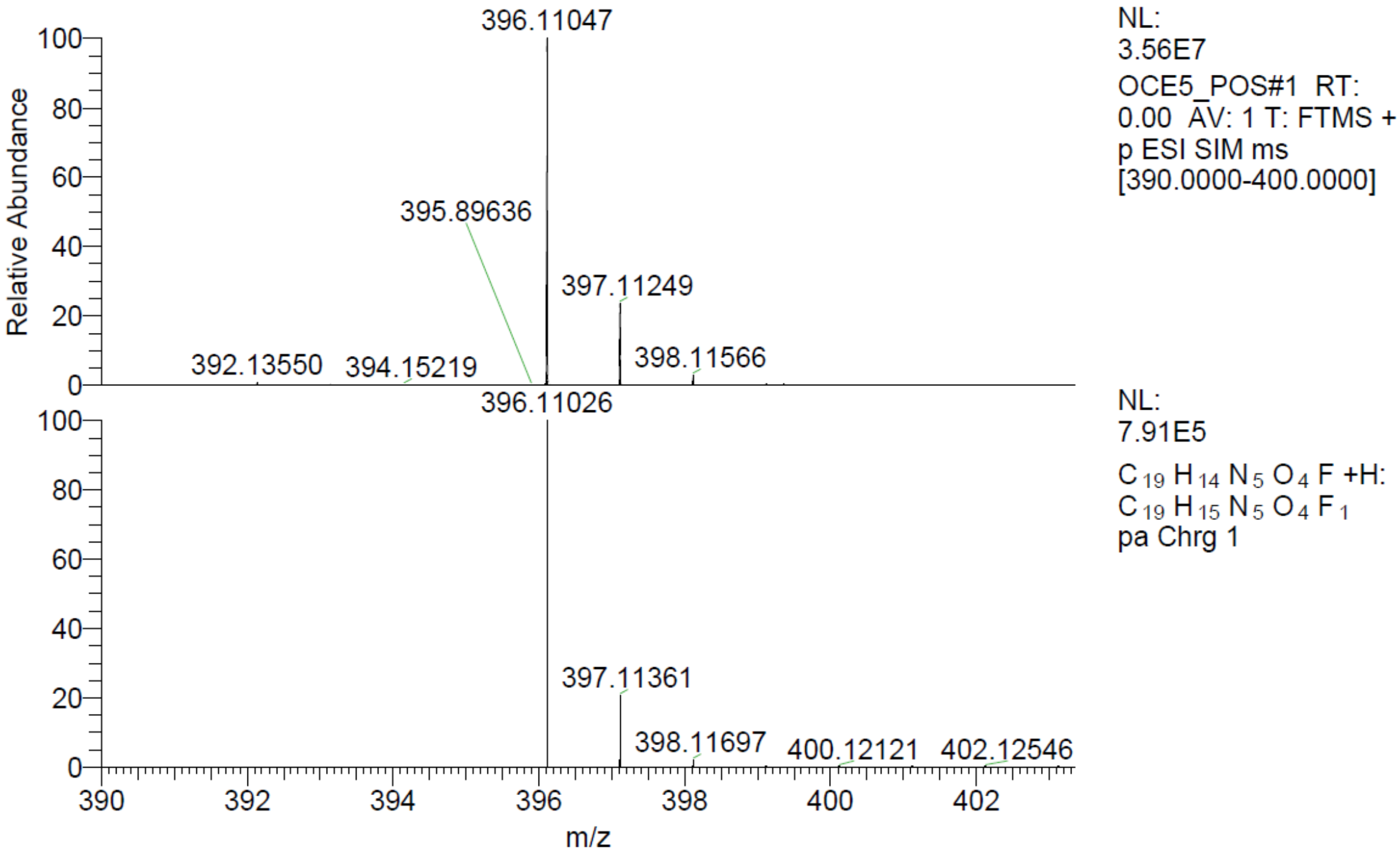

Figure S25. HRMS spectrum of compound **7e**.

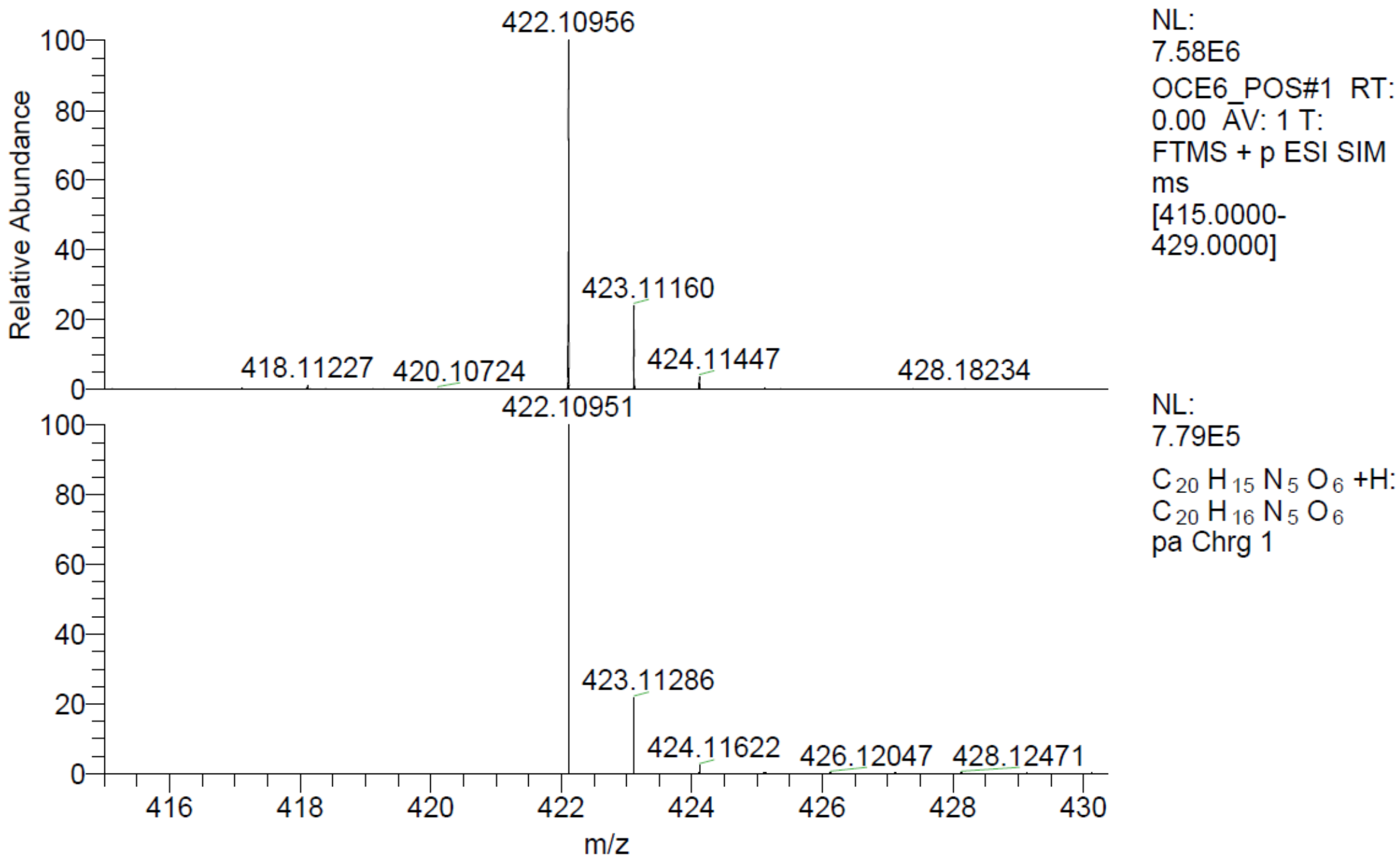

Figure S26. HRMS spectrum of compound **7f**.

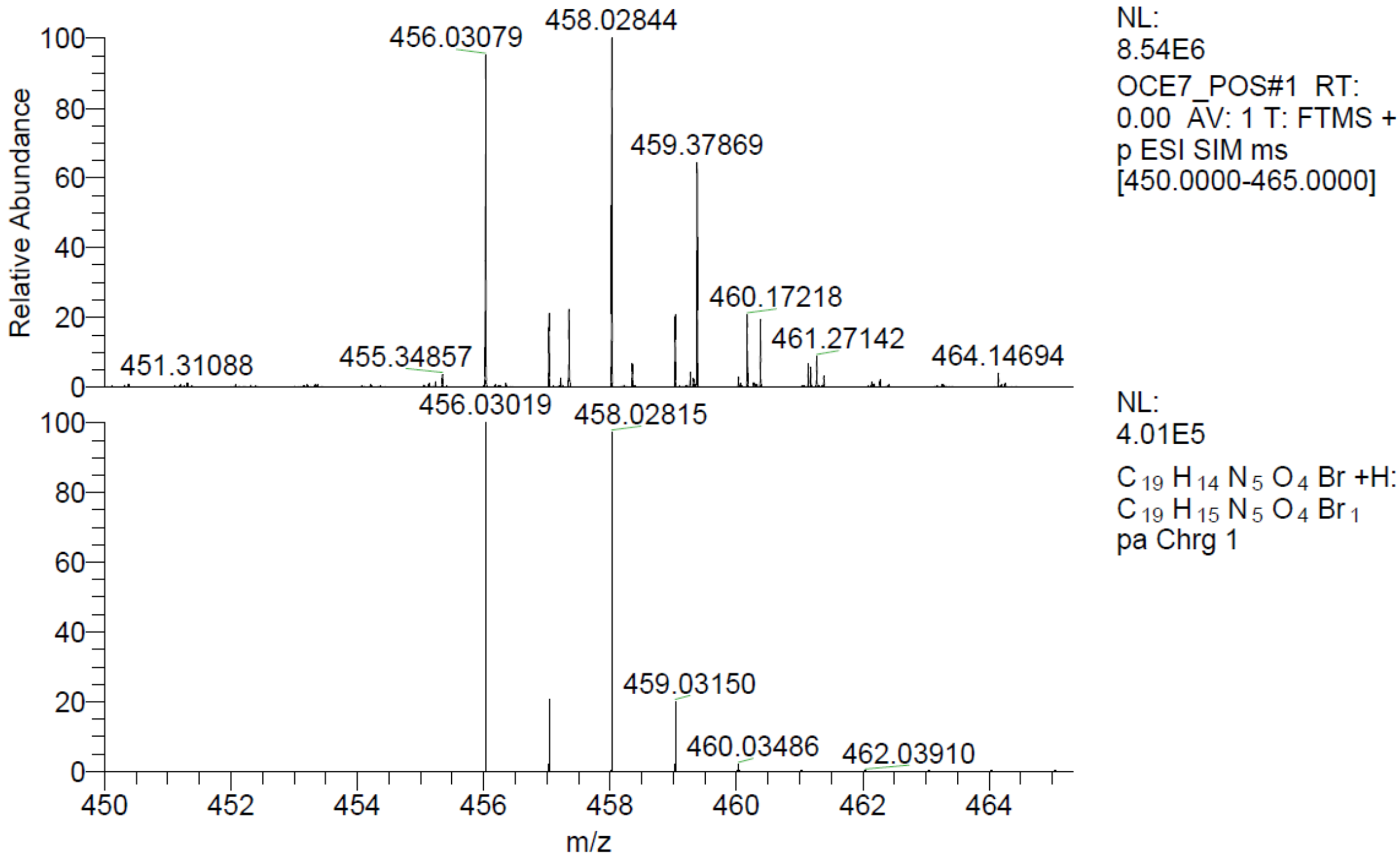

Figure S27. HRMS spectrum of compound **7g**.

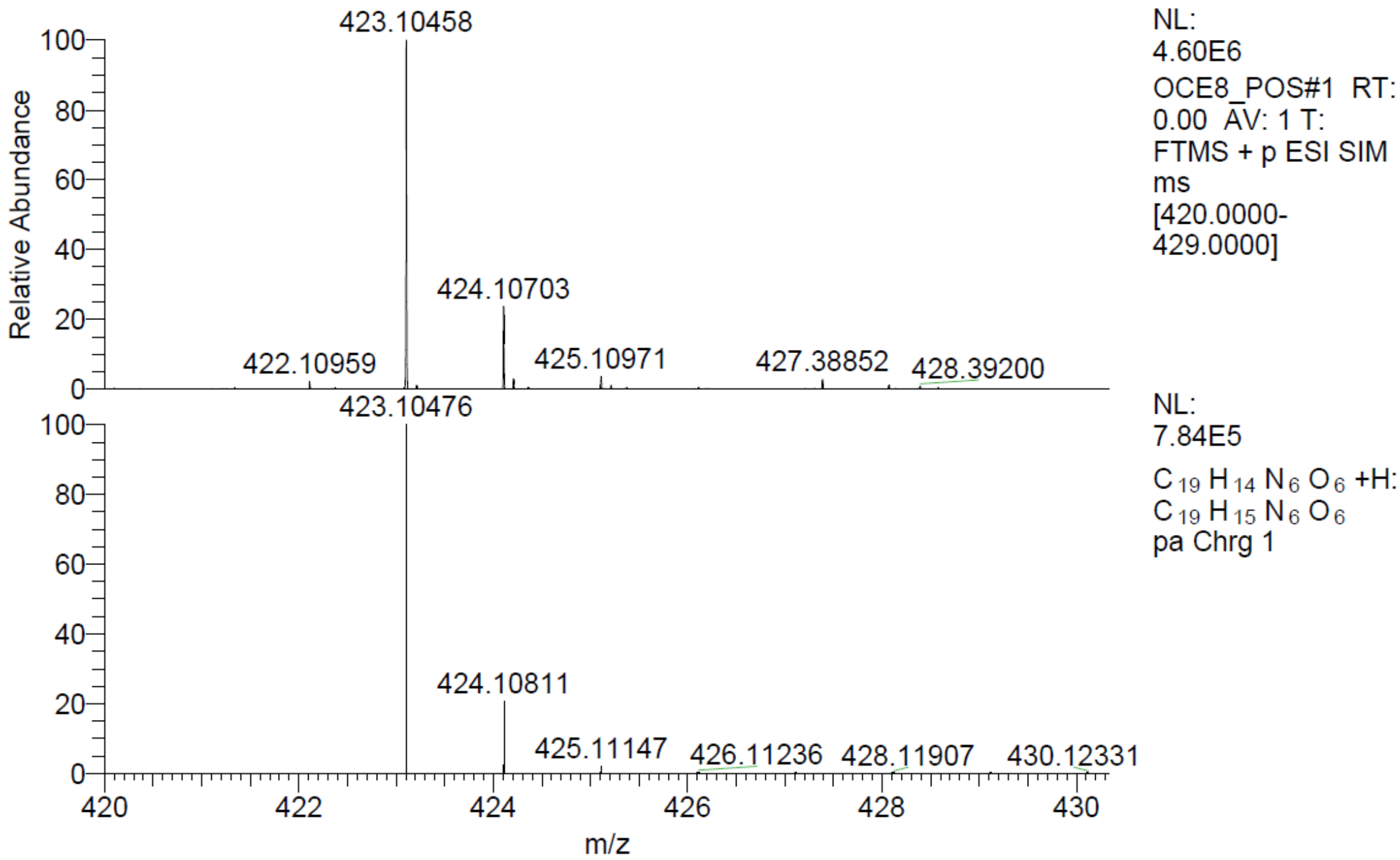

Figure S28. HRMS spectrum of compound **7h**.

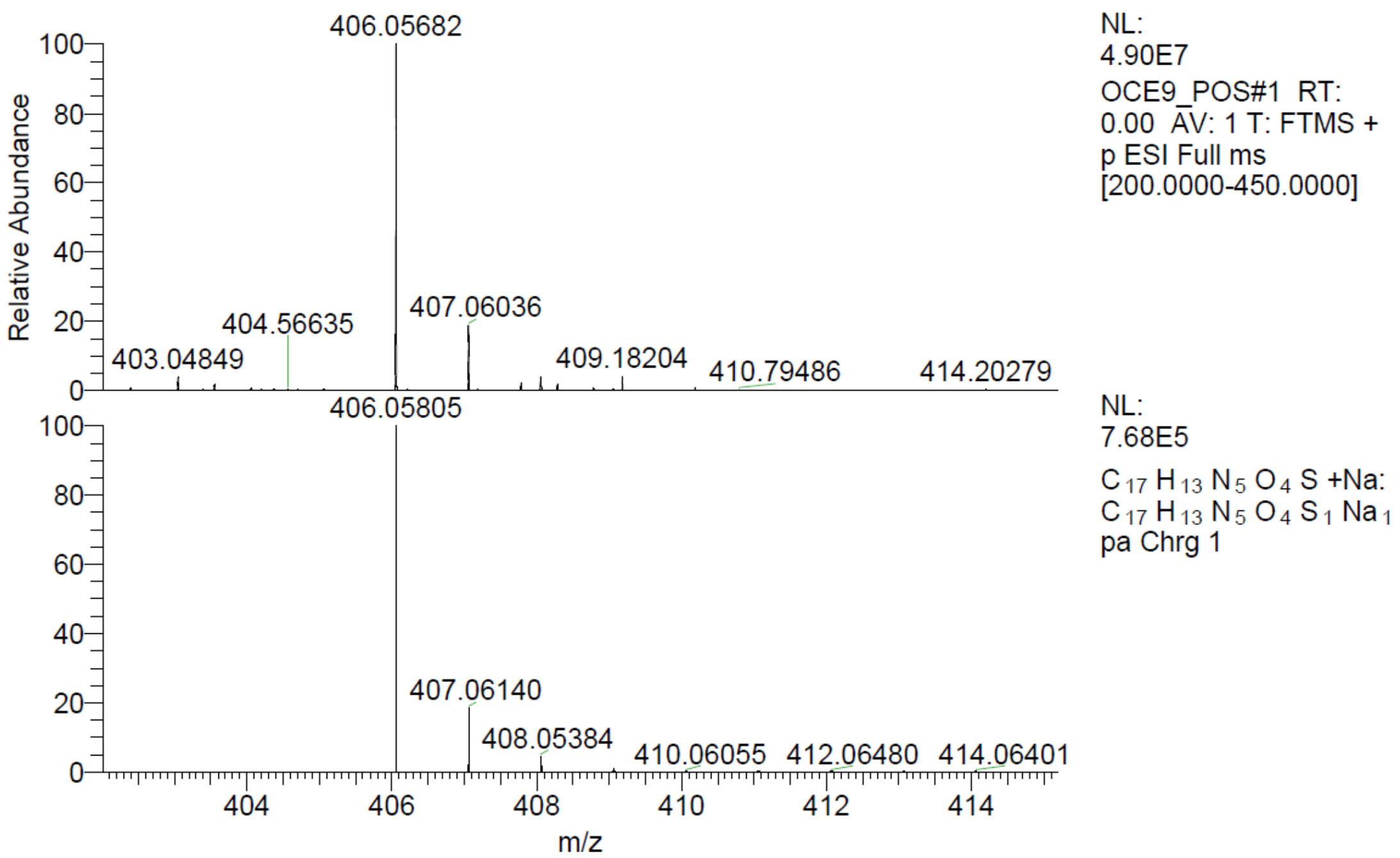

Figure S29. HRMS spectrum of compound **7i**.

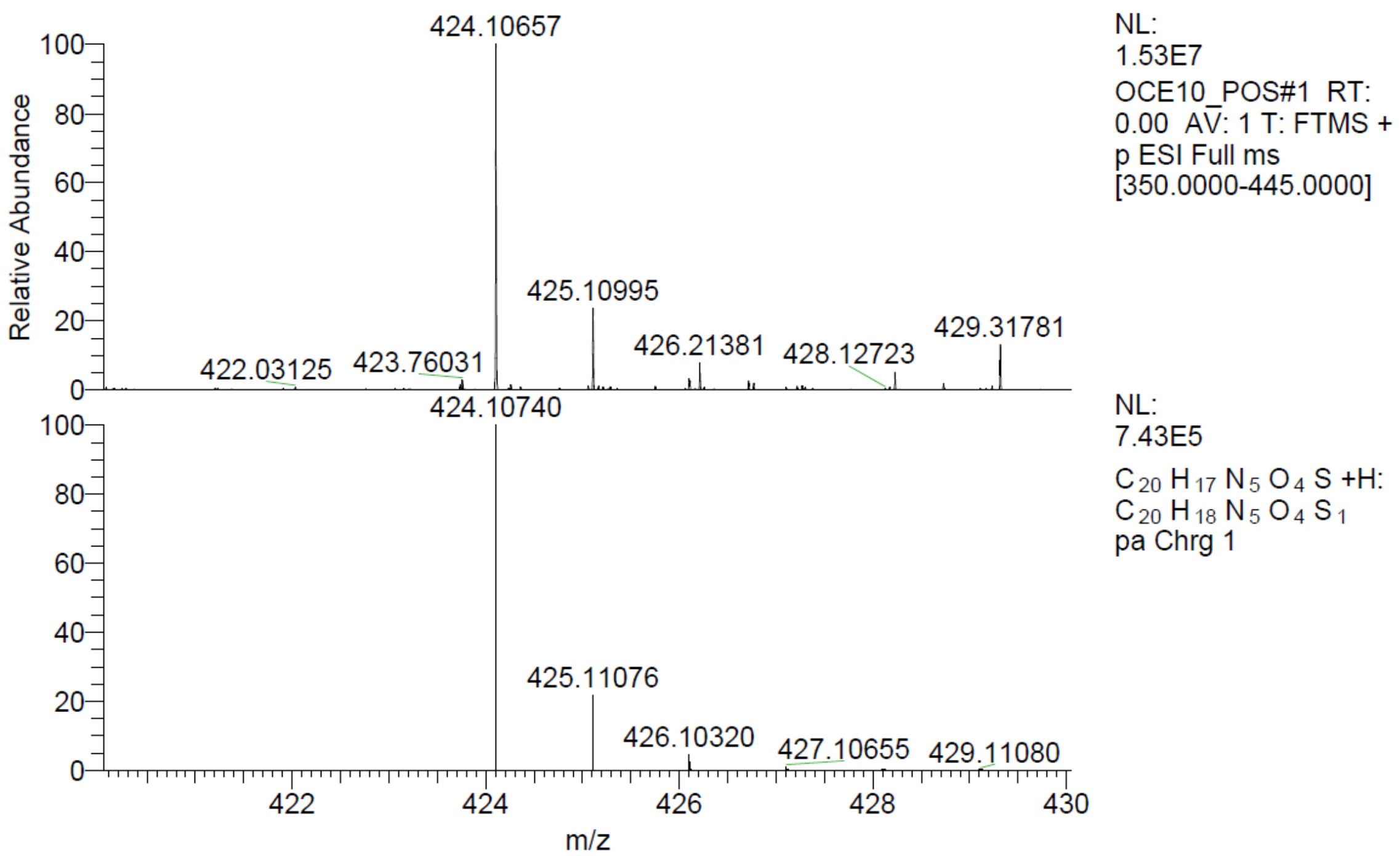

Figure S30. HRMS spectrum of compound **7j**.

Compounds' Main Interactions with the AT_1R (PDB ID: 4YAY).

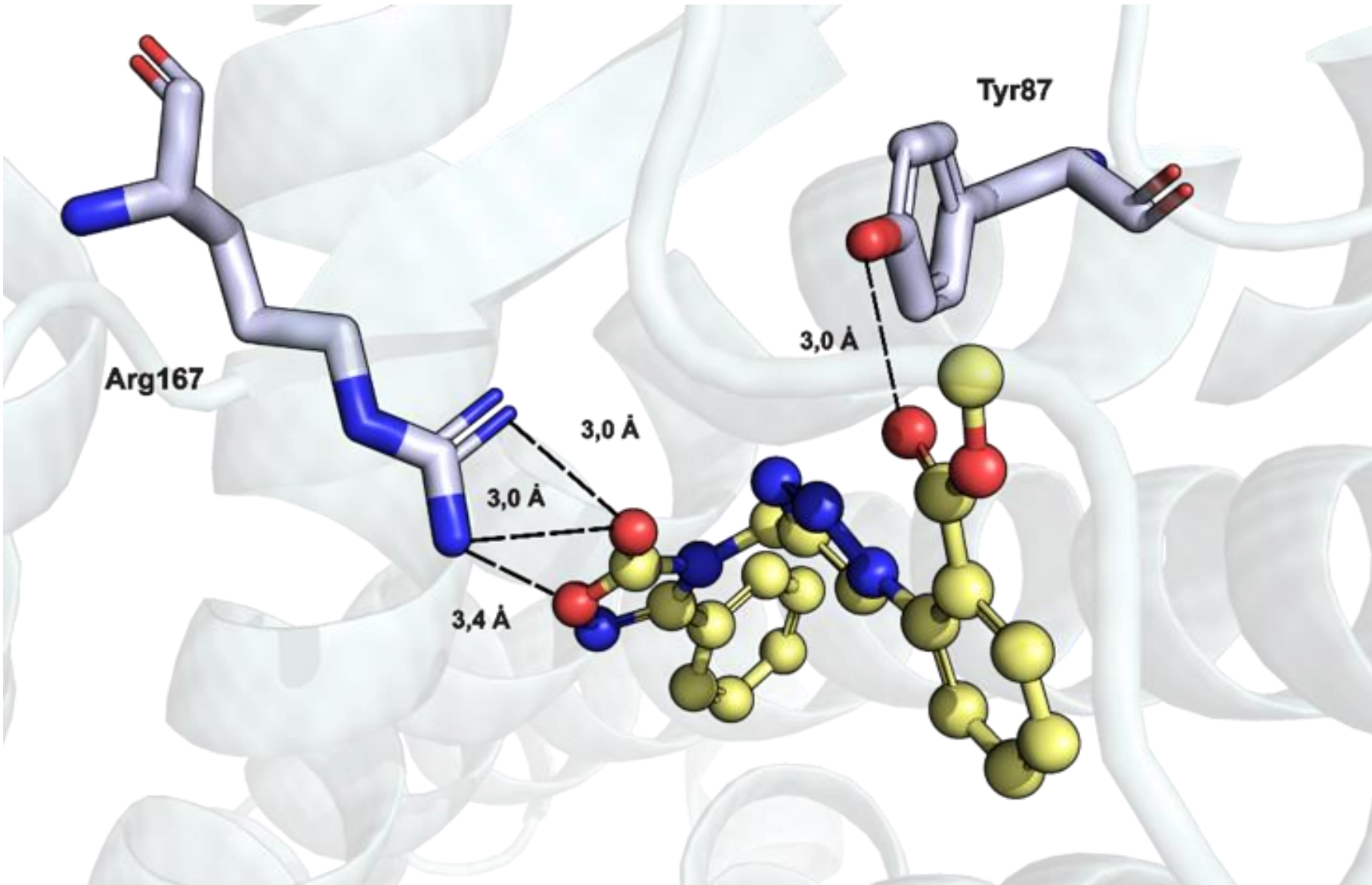

Figure S31. Interaction of compound **7a** with the AT_1R (PDB ID: 4YAY).

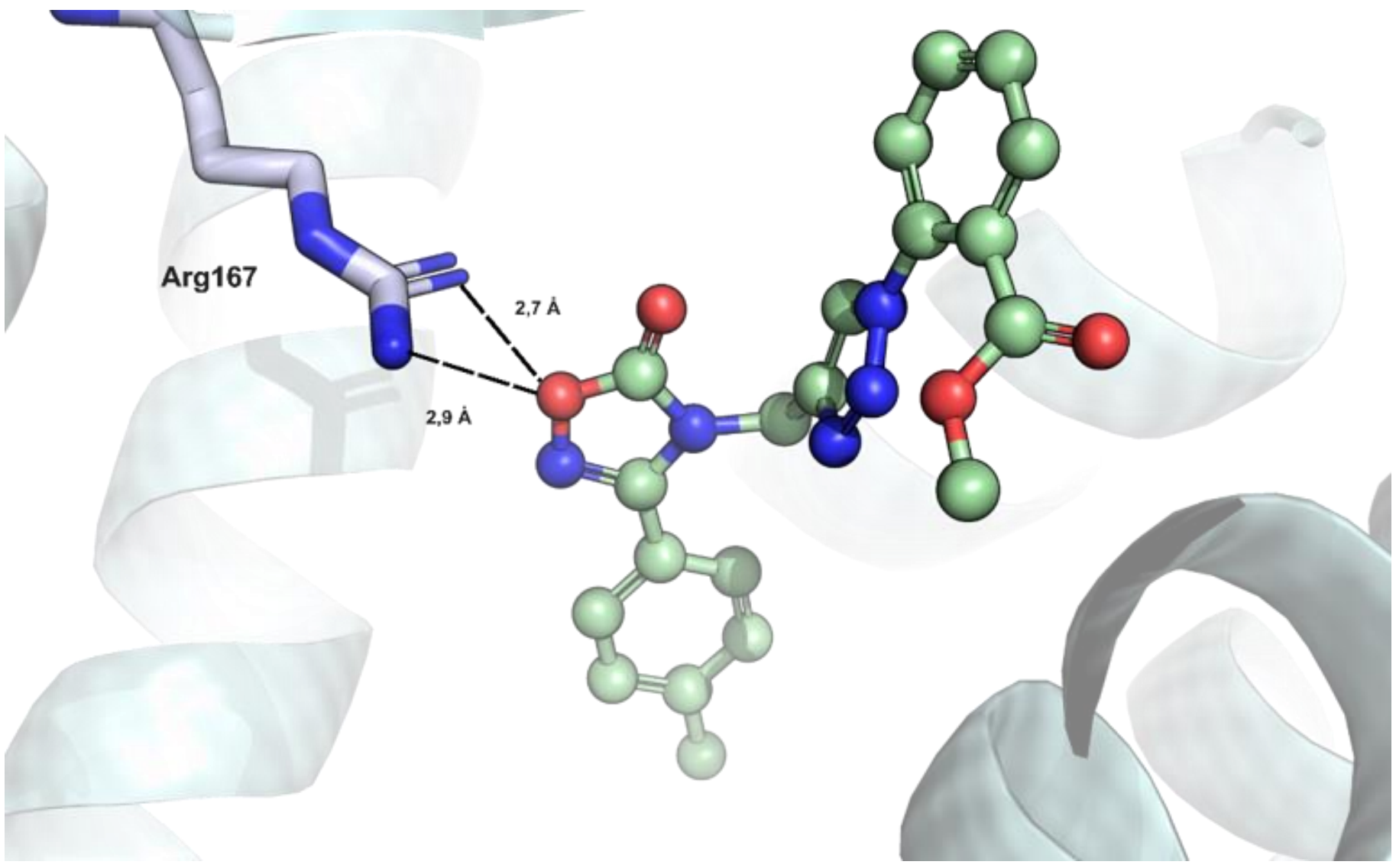

Figure S32. Interaction of compound **7b** with the AT_1R (PDB ID: 4YAY).

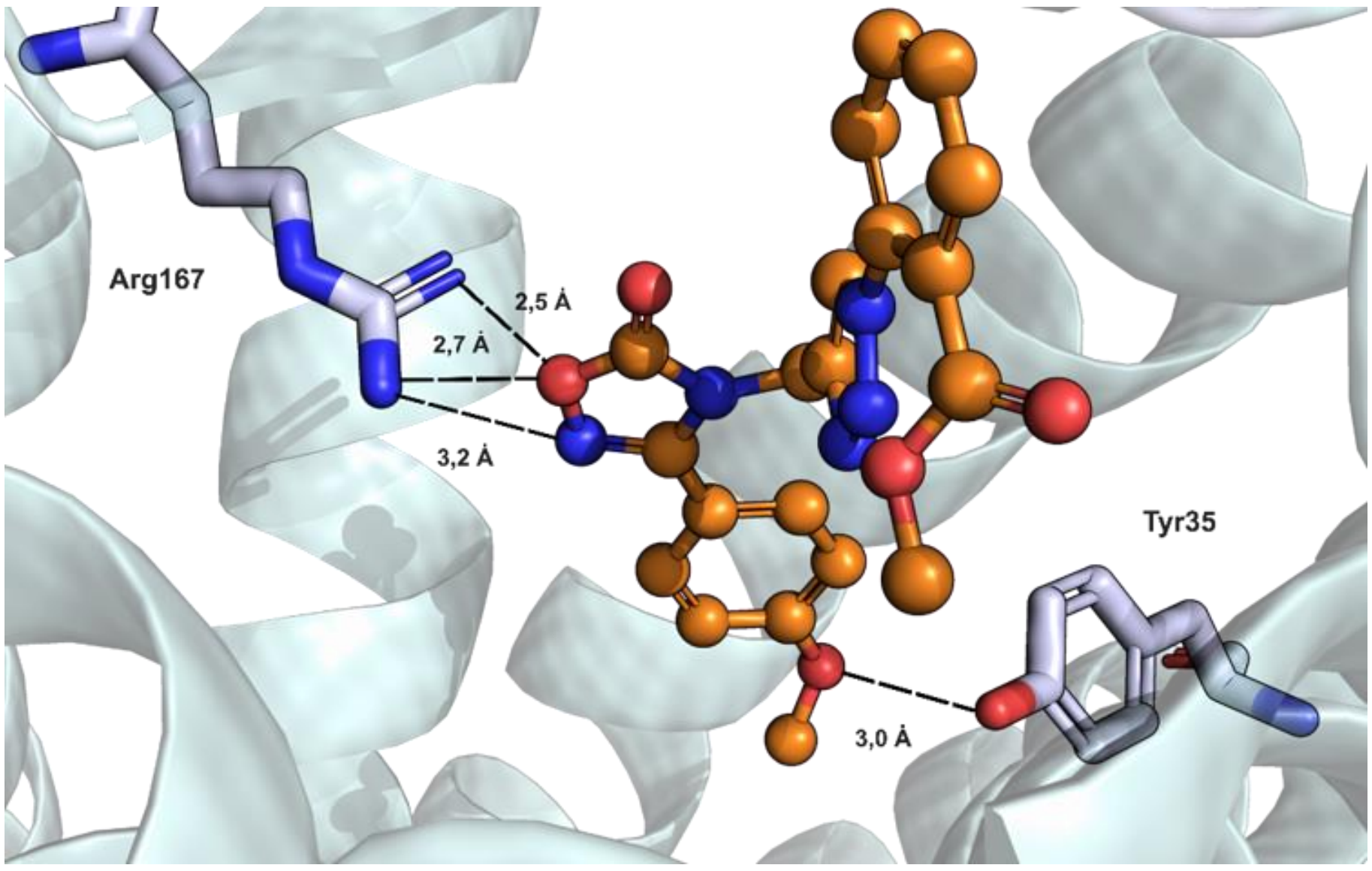

Figure S33. Interaction of compound **7c** with the AT_1R (PDB ID: 4YAY).

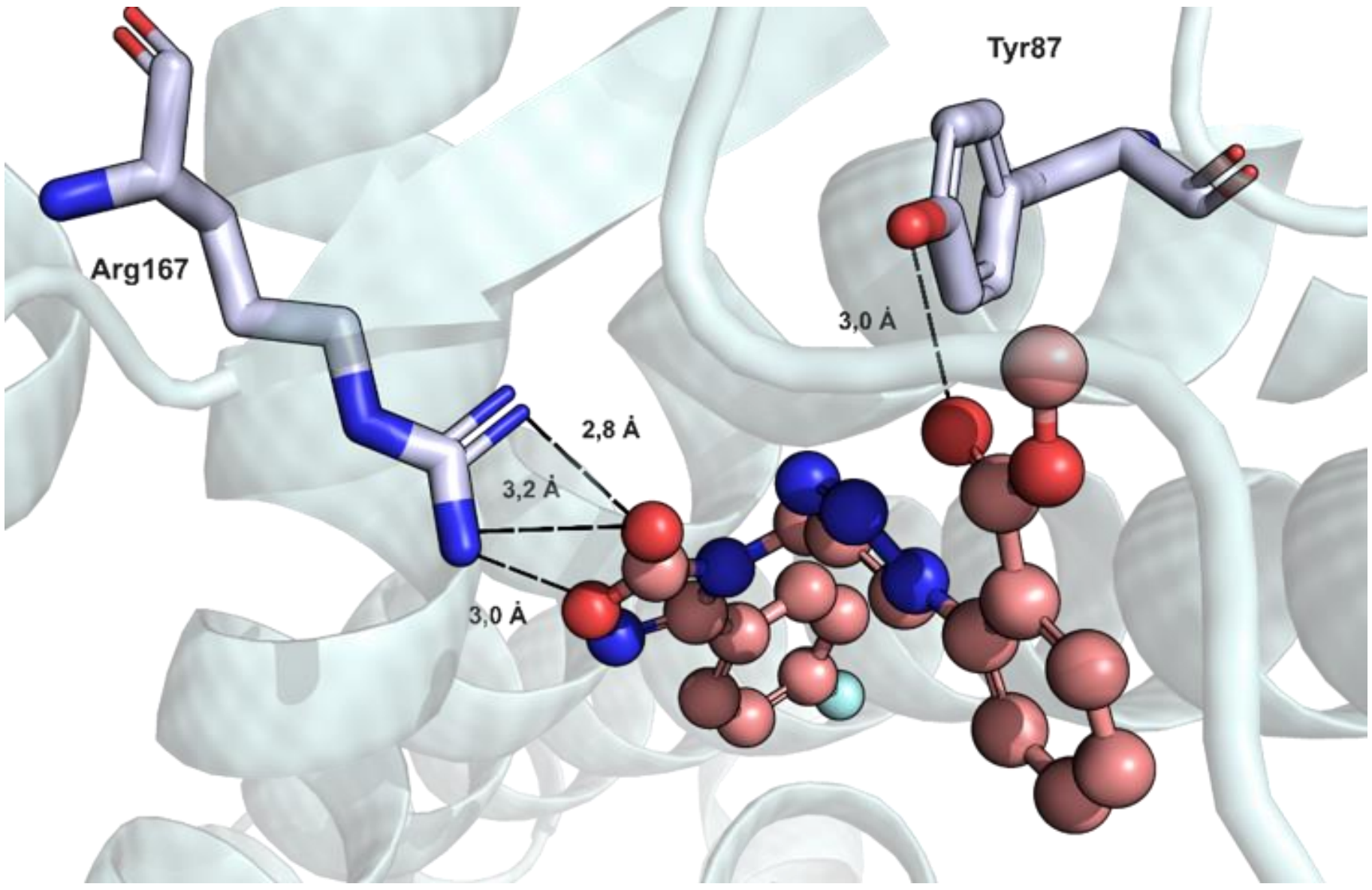

Figure S34. Interaction of compound **7d** with the AT_1R (PDB ID: 4YAY).

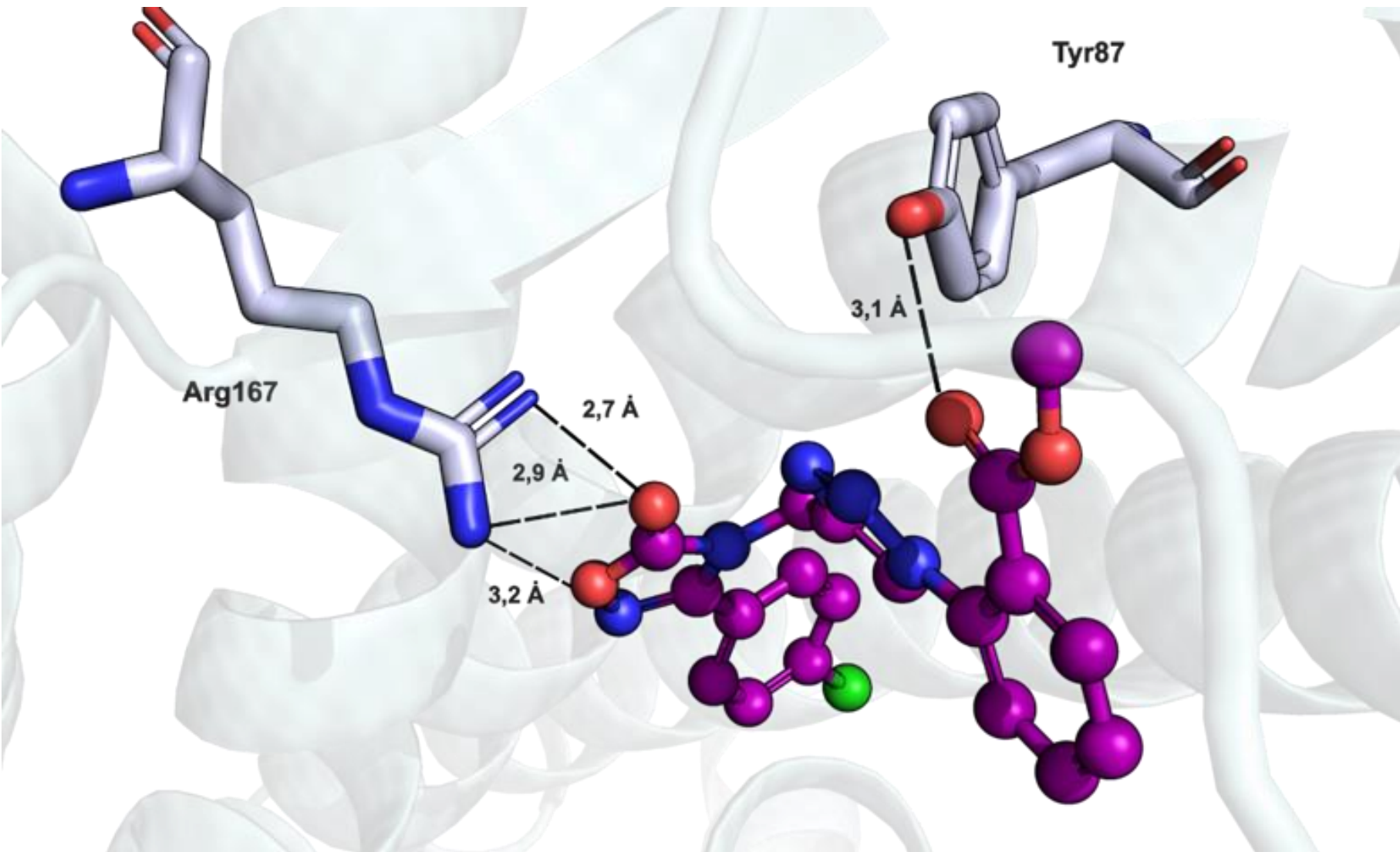

Figure S35. Interaction of compound **7e** with the AT_1R (PDB ID: 4YAY).

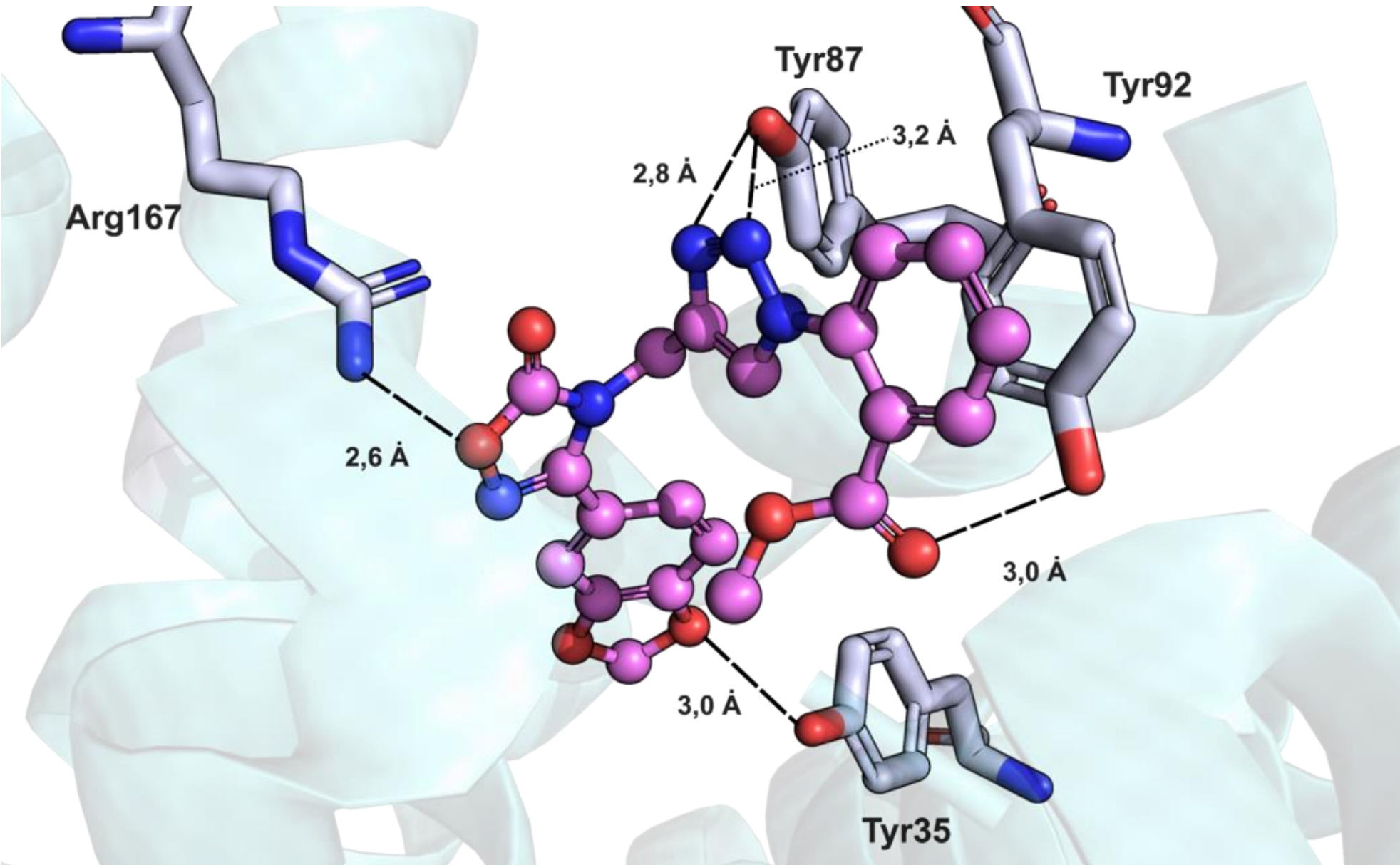

Figure S36. Interaction of compound **7f** with the AT_1R (PDB ID: 4YAY).

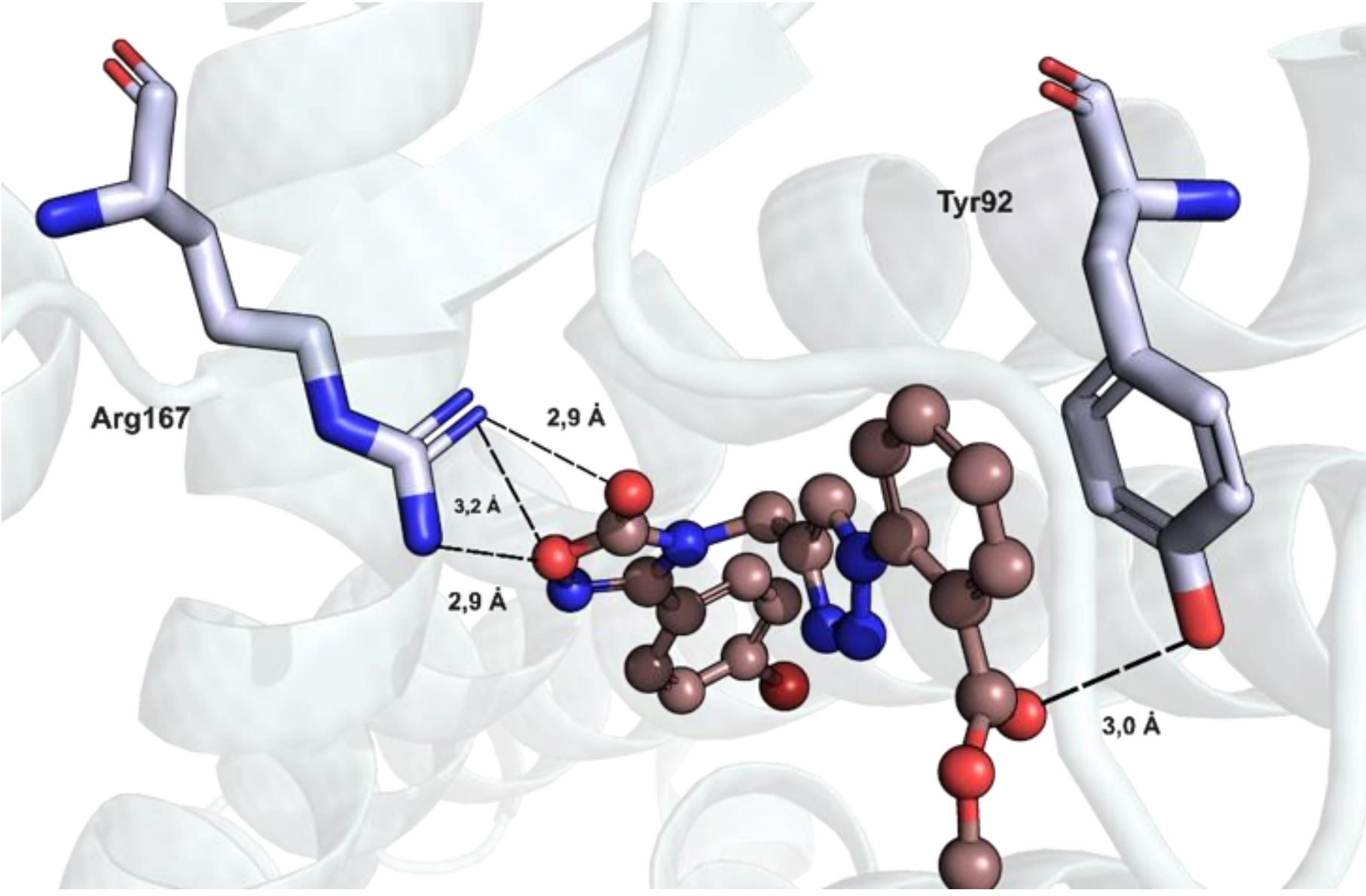

Figure S37. Interaction of compound **7g** with the AT_1R (PDB ID: 4YAY).

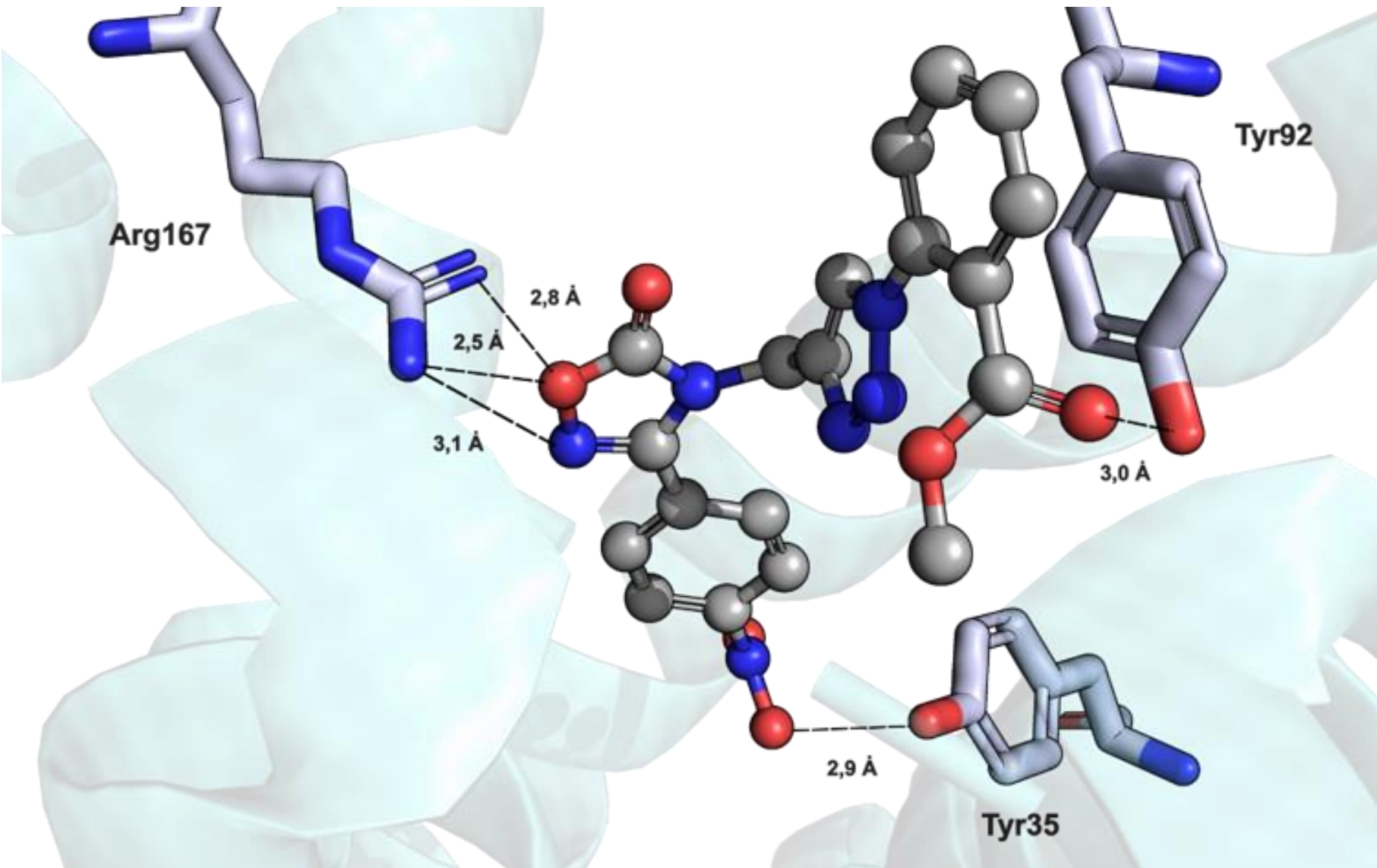

Figure S38. Interaction of compound **7h** with the AT_1R (PDB ID: 4YAY).

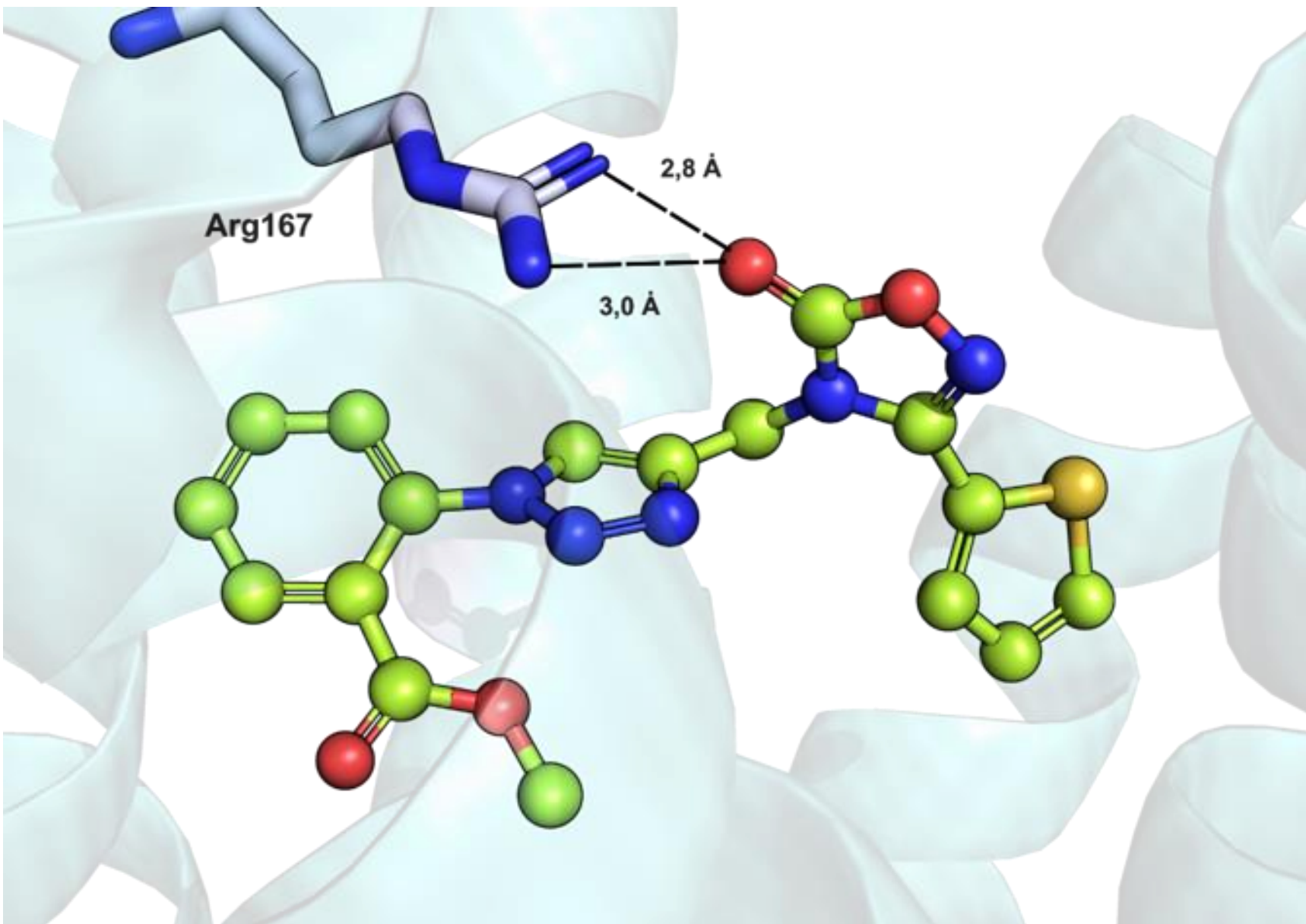

Figure S39. Interaction of compound **7i** with the AT_1R (PDB ID: 4YAY).

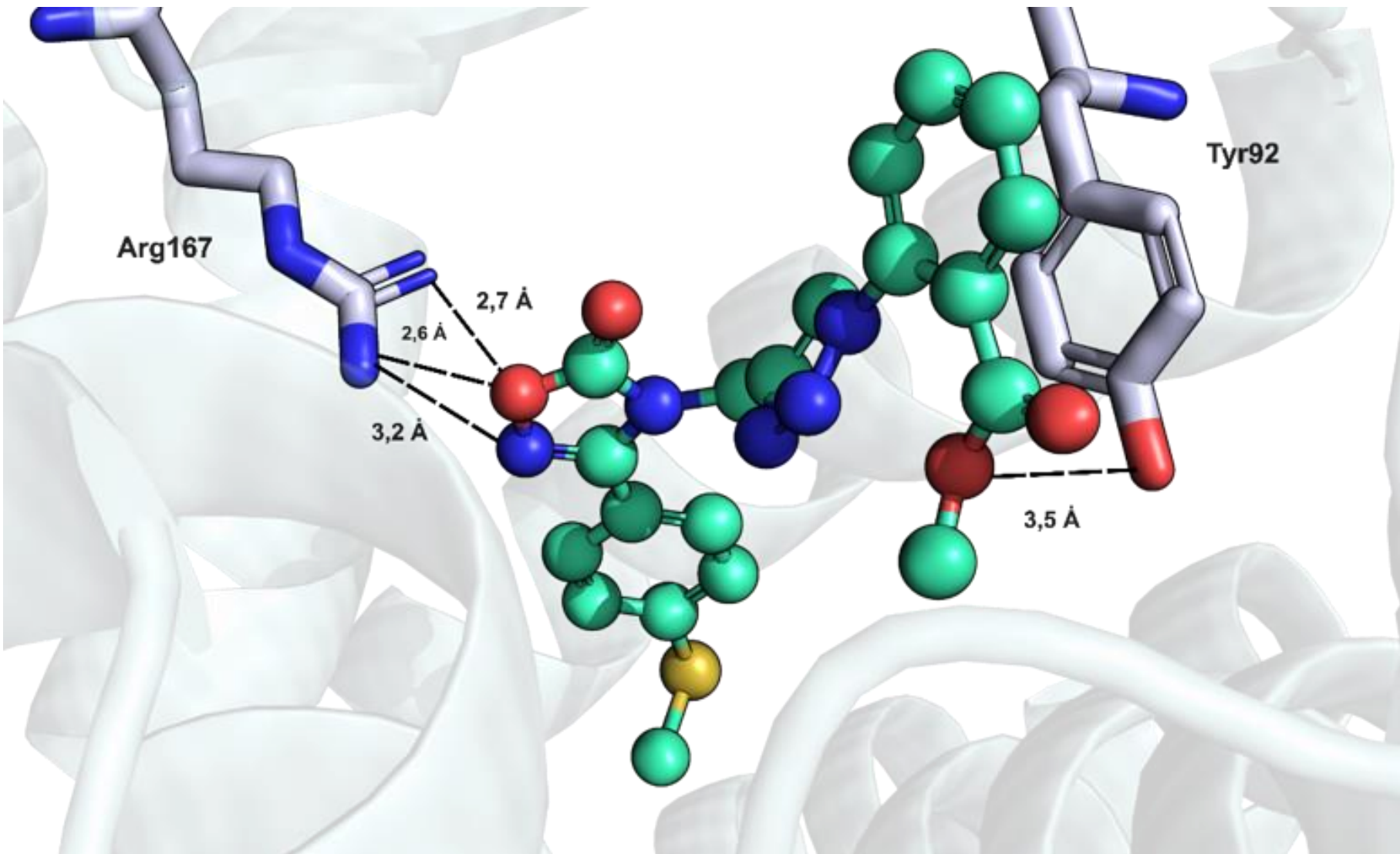

Figure S40. Interaction of compound **7j** with the AT_1R (PDB ID: 4YAY)

SwissADME Data for compounds 7a and 7f

Table S1. ADME predictions for **7a** and **7f**

	**7a**	**7f**
**SMILES**	COC(=O)c1ccccc1n1nnc(c1)Cn1c(=O)onc1c1ccccc1	COC(=O)c1ccccc1n1nnc(c1)Cn1c(=O)onc1c1ccc2c(c1)OCO2
**Formula**	$C_{19}H_{15}N_5O_4$	$C_{20}H_{15}N_5O_6$
**MW**	377.35	421.36
**#Heavy atoms**	28	31
**#Aromatic heavy atoms**	22	22
**Fraction Csp³**	0.11	0.15
**#Rotatable bonds**	6	6
**#H-bond acceptors**	7	9
**#H-bond donors**	0	0
**MR**	98.14	104.21
**TPSA**	105.04	123.50
**iLOGP**	2.55	2.86
**XLOGP3**	2.42	2.23

**WLOGP**	1.92	1.65
**MLOGP**	2.25	1.89
**Silicos-IT Log P**	1.67	1.51
**Consensus Log P**	2.16	2.03
**ESOL Log S**	-3.89	-3.99
**ESOL Solubility (mg/ml)**	0.0487	0.0435
**ESOL Solubility (mol/l)**	1.29×10^{-4}	1.03×10^{-4}
**ESOL Class**	Soluble	Soluble
**Ali Log S**	-4.27	-4.46
**Ali Solubility (mg/ml)**	0.0203	0.0147
**Ali Solubility (mol/l)**	5.39×10^{-5}	3.48×10^{-5}
**Ali Class**	Moderately soluble	Moderately soluble
**Silicos-IT LogSw**	-5.56	-5.28
**Silicos-IT Solubility**	0.00104	0.00223

**(mg/ml)**		
**Silicos-IT Solubility (mol/l)**	2.75×10^{-6}	5.28×10^{-6}
**Silicos-IT class**	Moderately soluble	Moderately soluble
**GI absorption**	High	High
**BBB permeant**	No	No
**Pgp substrate**	No	No
**CYP1A2 inhibitor**	Yes	No
**CYP2C19 inhibitor**	Yes	Yes
**CYP2C9 inhibitor**	Yes	Yes
**CYP2D6 inhibitor**	No	No
**CYP3A4 inhibitor**	No	Yes
**log Kp (cm/s)**	-6.88	-7.29
**Lipinski #violations**	0	1
**Ghose #violation**	0	0

**s**		
**Veber #violations**	0	0
**Egan #violations**	0	0
**Muegge #violations**	0	0
**Bioavailability Score**	0.55	0.55
**PAINS #alerts**	0	0
**Brenk #alerts**	0	0
**Leadlikeness #violations**	1	1
**Synthetic Accessibility**	3.55	3.68
